# Impact of waterpipe smoking on the salivary microbiome

**DOI:** 10.3389/froh.2023.1275717

**Published:** 2023-11-09

**Authors:** Nikitha Lalindri Mareena Senaratne, Chun Wie Chong, Lim Shu Yong, Ling Fong Yoke, Divya Gopinath

**Affiliations:** ^1^ School of Medicine, International Medical University, Kuala Lumpur, Malaysia; ^2^ School of Pharmacy, Monash University, Kuala Lumpur, Malaysia; ^3^ Monash University Malaysia Genomics Facility, School of Science, Monash University Malaysia, Selangor Darul Ehsan, Malaysia; ^4^ College of Dentistry, Ajman University, Ajman, United Arab Emirates; ^5^ Centre of Medical and Bio-Allied Health Sciences Research, Ajman University, Ajman, United Arab Emirates

**Keywords:** tobacco, waterpipe smoking, salivary microbiome, oral microbiota, 16S rRNA gene

## Abstract

**Background:**

While oral mirobial dysbiosis due to tobacco smoking has been studied thoroughly, there is limited data on the effect of waterpipe smoking on the oral microbiome. This study aims to compare the salivary microbiome between waterpipe smokers and non-smokers.

**Materials and methods:**

Unstimulated saliva samples were collected from 60 participants, 30 smokers and 30 non-smokers in Kuala Lumpur and Klang Valley, Malaysia. DNA extraction was performed using the Qiagen DNA mini kit, and the 16S rRNA bacterial gene was amplified and sequenced using the Illumina MiSeq platform. Sequencing reads were processed using DADA2, and the alpha and beta diversity of the bacterial community was assessed. Significantly differentiated taxa were identified using LEfSe analysis, while differentially expressed pathways were identified using MaAsLin2.

**Results:**

A significant compositional change (beta diversity) was detected between the two groups (PERMANOVA *P* < 0.05). Specifically, the levels of phylum Firmicutes and genus *Streptococcus* were elevated in smokers, whereas phylum Proteobacteria and genus *Haemophilus* were depleted compared to non-smokers. At the species level, *Streptococcus oralis, Streptococcus salivarius,* and *Streptococcus gingivalis* were enriched in smokers. We observed significant differences in the abundance of thirty-seven microbial metabolic pathways between waterpipe smokers and non-smokers. The microbial pathways enriched in smokers were those implicated in polymer degradation and amino acid metabolism.

**Conclusion:**

The taxonomic and metabolic profile of the salivary microbiome in waterpipe smokers compared to healthy controls exhibited a paradigm shift, thus, implying an alteration in the homeostatic balance of the oral cavity posing unique challenges for oral health.

## 1. Introduction

The human oral cavity harbors a diverse microbial community comprising over 700 species of bacteria or phylotypes that play a commensal role in protecting oral and systemic health (1). These species have been identified by cultivation or advancing culture-independent molecular approaches (2). Some of these bacteria attach to the mouth's soft and hard tissue surfaces, forming biofilms in a structurally organized matrix and inducing inflammatory immune responses in the host with changes in their growth rates or compositions (3). The salivary microbiota comprises bacteria shed from oral surfaces, particularly the dorsal surface of the tongue, and changes in the salivary microbial community can be vital in diagnosing and monitoring oral and systemic diseases (1, 4).

The core microbial composition within the oral cavity is similar. Still, the type of species may differ depending on genetic susceptibility, diet, antibiotic usage, hormonal factors, tobacco and alcohol exposure, and recurrent pathogenic infections of the host (5). Any disturbance to their equilibrium results in oral dysbiosis, altering oral and systemic health through several pathophysiological processes linked to disease. Dysbiosis has been implicated in oral cavity diseases such as gingivitis, periodontitis, and oral cancer (6, 7).

Amidst different types of tobacco, including chewing tobacco, e-cigarettes, and waterpipe, the association between the oral microbiome and cigarette smoking has gained increasing attention due to the significant addictive constituents of cigarette smoke, which modifies the host's immune responses (8). Smoking leads to the loss of beneficial oral species and alterations in the pathogens by interacting with numerous host cells and extracellular matrix components. It ultimately leads to the risk of disease development (9). This alteration either increases the density of the bacterial pathogens or decreases the prevalence of other bacteria (10). Waterpipes, known as shisha, hookah, argileh, or hubble bubble, depending on the region, is a popular form of smoking and have gained enormous popularity worldwide (11). Influenced by cultural traditions in the Middle East and parts of Asia, it has historically been used primarily by males. A typical waterpipe device contains the head, body, and bowl. The head includes the coal where the tobacco, commonly called *Maassel*, is heated to produce the smoke. The body consists of a stem connecting the head to a water-filled bowl. A hose lets the smoker draw out and inhale the smoke (11). Flavored tobacco comprises shredded tobacco leaves, glycerol, and other additives (10).

Current evidence suggests that the smoke from waterpipes contains toxic constituents and is associated with adverse health effects. The smoke has a similar toxicant profile as cigarettes but of a different magnitude. According to the CDC, an hour of waterpipe smoking is equivalent to smoke inhalation from 200 regular cigarettes or 90,000 ml of smoke (12). A recent study found that species like *Acinetobacter* and *Moraxella* in the subgingival fluid were only present in waterpipe users compared to non-smokers. In addition, it was also reported that *C. Albicans, P. gingivalis,* and *P. intermedia* were also higher in smokers (13).

Massively parallel sequencing technologies have helped reveal the complex nature of the oral microbial community. Understanding the healthy oral microbiome enables an exploration of the functional and metabolic alterations in disease (14). The impact of waterpipe smoking on the salivary microbiota is currently understudied. The main objective of this study is to identify the microbial composition, i.e., bacterial phylogeny and taxonomy in the saliva of waterpipe smokers relative to non-smokers using the sequencing of the conserved 16S rRNA gene (14). Its presence in all bacteria permits the identification of bacteria and differentiating between closely related species. It is the most commonly used genetic marker and is considered the gold standard for microbial community profiling (15).

## 2. Materials and methods

### 2.1. Study design and recruitment of participants

This study was approved by the International Medical University Joint Research Committee under project number BMScI-2021(01). This study was performed according to the Declaration of Helsinki guidelines in medical research involving human subjects (16), and informed consent was obtained from all the volunteers before sample collection. The study population comprised 60 healthy participants, 30 water pipe smokers, and 30 non-smokers. The study subjects were age- and gender-matched. The inclusion criteria were as follows: Males aged 18–40 years, current waterpipe smokers, did not engage in other forms of smoking, are not on any medications currently or in the past 3 months and were willing to provide informed consent. Subjects were excluded if they had any current active infections, acute illnesses, or the presence of any self-reported gum diseases. Study subjects were recruited from Kuala Lumpur and Klang Valley, Malaysia.

### 2.2. Sample collection

Subjects were given a 50 ml Falcon tube to spit 1 ml of saliva without rinsing or washing out their mouth prior. For waterpipe smokers, saliva was collected while the subjects were smoking a waterpipe. The subjects were asked to secure the caps tightly, and the samples were transported on ice and immediately frozen at -80°C. The samples were brought to the International Medical University, Malaysia, for DNA extraction and stored at -80°C.

### 2.3. Sample processing and 16s rRNA gene sequencing

Qiagen DNA mini kit (Qiagen, Gemrnay) was used to extract DNA from the salivary samples. 500 μl of the saliva was mixed with 1 ml phosphate buffered saline (PBS) and centrifuge for 1,000 rpm at 4,000 g. The supernatant was discarded, and the saliva was reconstituted in 180 ul PBS. 20 μl of proteinase K was added into the microcentrifuge tube. 200 μl of reconstituted saliva sample was added along with 200 μl of Buffer AL. The tube was then mixed by pulse-vortexing for 15 s and incubated at 56°C for 15–20 min. After spinning down the tubes, 200 μl of ethanol was added to the sample, mixed by pulse-vortexing, and spun down for 15 s. The samples were then transferred and centrifuged at 6,000 × g (8,000 rpm) for 1 min 500 μl of buffer AW1 was added and centrifuged at 6,000 × g (8,000 rpm) for 1 min. After discarding the filtrate, 500 μl of buffer AW2 was added and centrifuged at 20,000 × g (14,000 rpm) for 3 min. The filtrate was discarded and centrifuged for 1 min. 200 μl of buffer AE was added and was incubated at room temperature (15–25°C) for 1 min and then centrifuged at 6,000 × g (8,000 rpm) for 1 min. The samples were stored at -20°C until further use.

DNA concentration and sample purity (A260/A280) were tested using a Tecan microplate reader (Tecan, Switzerland). Sample integrity was assessed using agarose gel electrophoresis. The bacterial DNA was amplified using standard PCR primers targeting the 16S rRNA gene (17). The primer sequence used is as follows; Forward primer TCGTCGGCAGCGTCAGATGTGTATAAGAGACAGCCTACGGGNGGCWGCAG, Reverse primer—GTCTCGTGGGCTCGGAGATGTGTATAAGAGACAGGACTACHVGG GTATCTAATCC. After quantification by real-time PCR, equimolar amounts of qualified libraries were sequenced on the Illumina Miseq system (Illumina, San Diego) using the PE300 reagent kit (MGI, Japan) using a 2 × bp PE read configuration.

### 2.4. Bioinformatics and statistical analysis

DADA2 was used to perform quality filtering, contig merging and chimera removal (18). Briefly, the standard quality parameters were used: maxN = 0, maxEE = c(2,2), truncQ = 2, rm.phix = TRUE. The final dataset consisted of 2,908,518 sequences. Metacyc pathway abundance was estimated using Phylogenetic Investigation of Communities by Reconstruction of Unobserved States (PICRUSt2) (19).

The data was then converted into an abundance table and exported into the Phyloseq R program for further analysis. The alpha diversity was inferred based on the Shannon Diversity Index, Simpson Diversity Index, and Pielou's Evenness Index. The Shannon index is sensitive to species richness, whilst Simpson and Pielou's evenness indices are more sensitive to species evenness (20). Mann Whitney *U* test was performed to test if there was a significant difference between the smokers and the non-smokers by comparing the medians of the two groups.

The Beta diversity was inferred using Aitchinson's Distance-based Principal Component Analysis (PCA) and Permutational multivariate analysis of variance (PERMANOVA). Both alpha and beta diversity were calculated using the Microbiome R package (21). Separately, PERMANOVA was performed using the Vegan R package (22).

Significantly differentiated taxa were identified using Linear Discriminant Analysis Effect Size (LEfSe) analysis, while differentially expressed pathways were identified using MaAsLin2 (23, 24).

## 3. Results

### 3.1. Study population and samples

A total of 80 samples were collected from both smokers and non-smokers. However, 20 samples were removed due to low DNA concentration (<20 ng/µl) and quality. The final samples comprised 30 waterpipe smokers (mean age 31.1 ± 5.4) and 30 controls (mean age 21.5 ± 1.7). All samples yielded an A260/A280 ratio of 1.81–2.11. All samples were sequenced at a sequencing depth of > 100,000 reads (ranging 10,1060 to 264,760 reads). An average of 48,475 reads was retained after quality filtering and chimera removal.

### 3.2. Richness and diversity of the salivary microbiome

**Figure 1:**
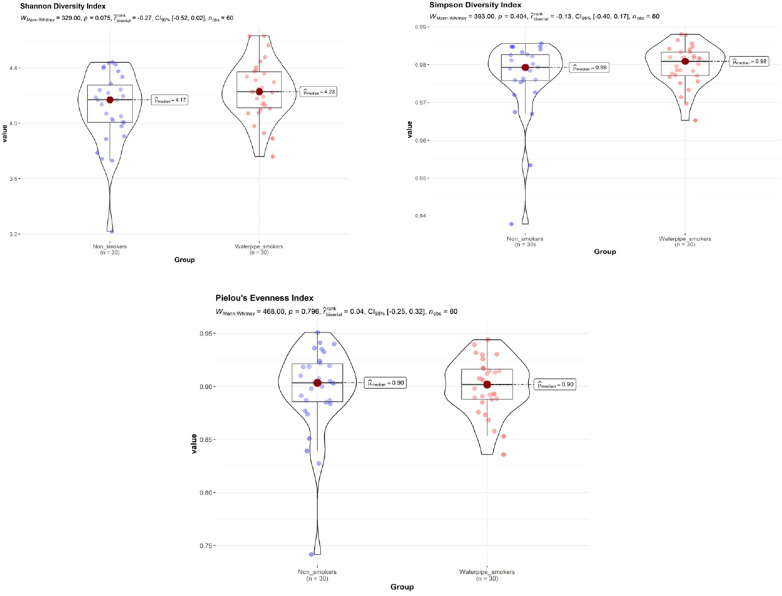
Shannon diversity index, simpson diversity index, and ‘Pielou's evenness index between smokers and non-smokers. No significant difference in Shannon diversity index, Simpson diversity index, and ‘Pielou's evenness index between smokers and non-smokers was detected (*P* > 0.05).

No significant difference in Shannon diversity index, Simpson diversity index, and ‘Pielou's evenness index between smokers and non-smokers was detected (*P* > 0.05; Figure 1), with and without adjustment of age and gender.

### 3.3. Differences in the relative abundance of oral bacteria in waterpipe smokers compared to non-smokers

Non-smokers had a higher relative abundance of *Proteobacteria*. In comparison, waterpipe smokers had increased phyla *Firmicutes* and a minor increase in *Bacteroidota*. However, *Fusobacteria* remained in similar abundance in both smokers and non-smokers. The most abundant phyla in both groups are shown in Supplementary Figure S1A.

Genera *Streptococcus* is predominant along with *Prevotella* and *Veillonella* in the saliva samples of smokers. In addition, *Porphyromonas* showed a minor increase, and *Lautropia* similarly showed a minor depletion in smokers relative to non-smokers, though not statistically significant. In the non-smoker group, *Haemophilus* and *Lautropia* were abundant compared to smokers*. Fusobacterium* and *Alloprevotella* remained of similar abundance in both groups. Overall, it is evident that *Streptococcus* was the most prevalent genus in smokers, with a significant depletion of *Haemophilus* comparatively. The ten most abundant genera shared by both groups are presented in Supplementary Figure S1B.

### 3.4. Waterpipe smoking was associated with changes in the oral microbial taxa

**Figure 2:**
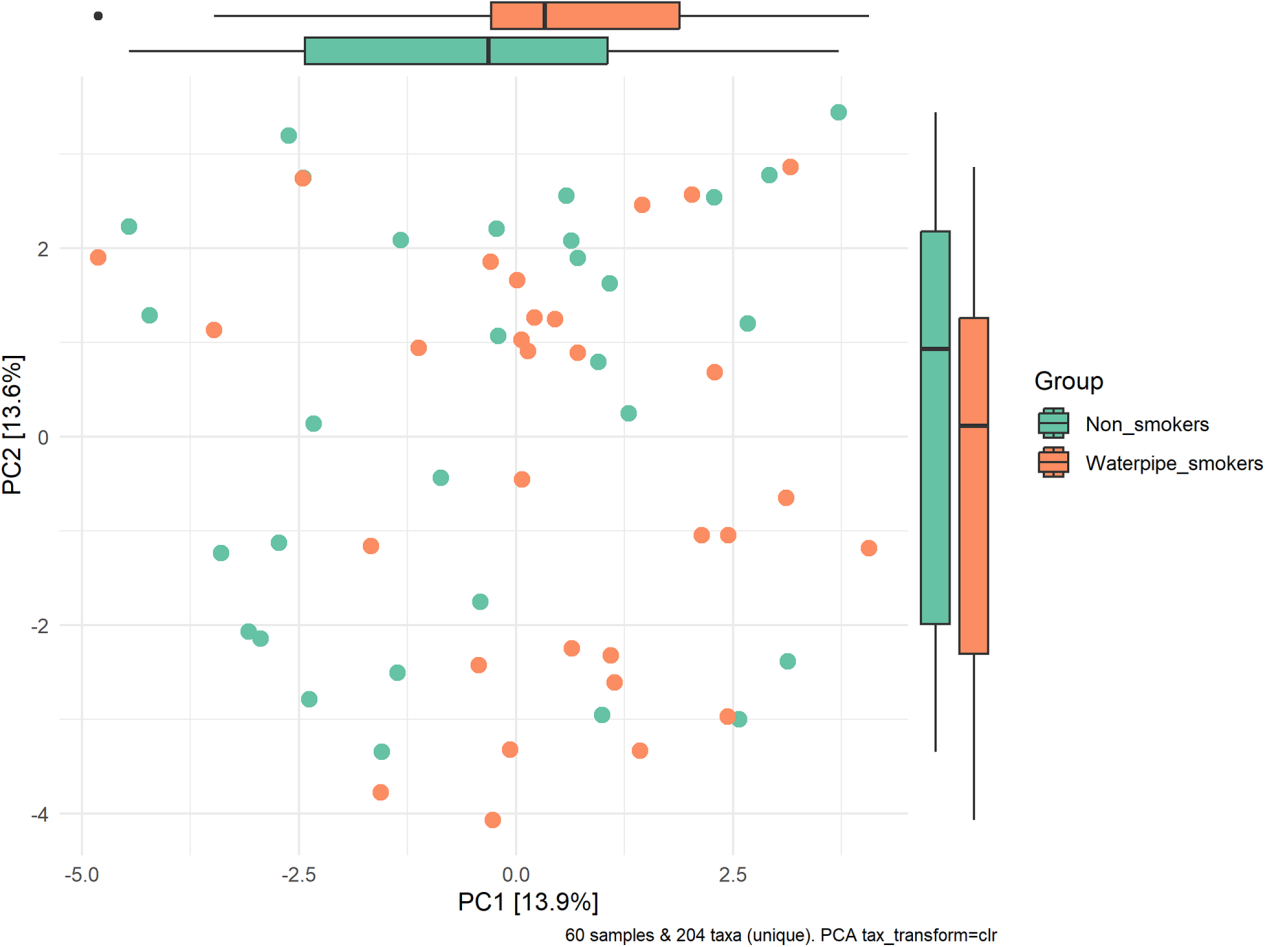
Sample distribution and beta-diversity. A significant difference in beta diversity was detected (PERMANOVA *P* = 0.001).

**Figure 3:**
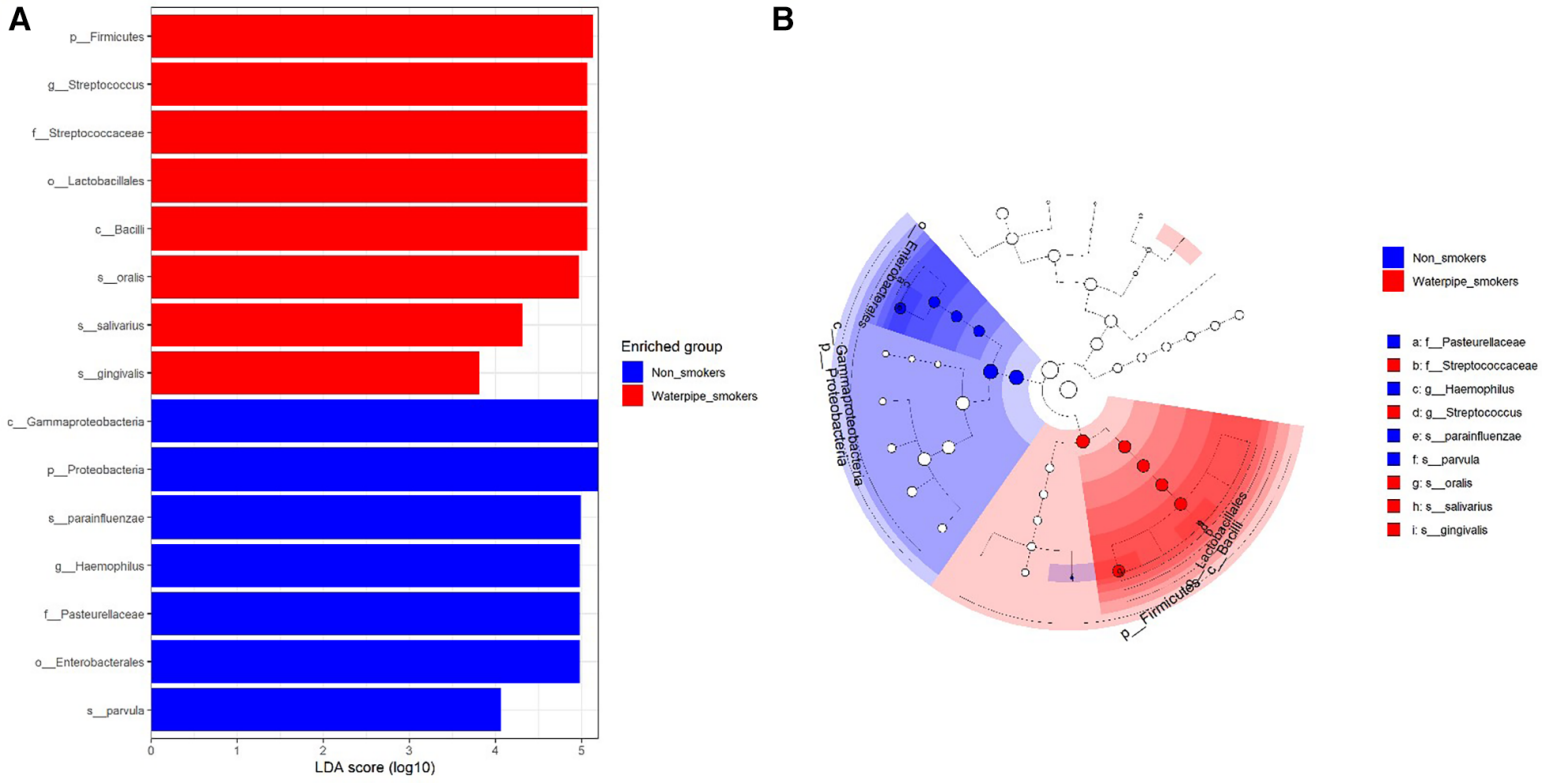
Differentially abundant taxa between smokers and non-smokers by lEfSe analyisis. 16 differentially abundant taxa between smokers and non-smokers reached significance with a log LDA score > 3.0 in the total population.

A significant difference in beta diversity was detected (PERMANOVA *P* = 0.001), and the sample distribution is illustrated in Figure 2. To further explore the significantly different bacteria among the groups, a LEfSe analysis was performed to differentiate between smokers and non-smokers. It is observed that 16 differentially abundant taxa between smokers and non-smokers reached significance with a log LDA score > 3.0 in the total population (Figure 3). At various taxa levels, *P. firmicutes (phyla), G. streptococcus (genus), F. streptococcaceae (family), O. lactobacillus (order), C. bacilli (class)* were significantly enriched in waterpipe smokers. The most enriched species in waterpipe smokers include *S. oralis, S. salivarius,* and *S. gingivalis*. *P. proteobacteria (phyla), C. gammaproteobacteria (class), G. Haemophilus (genus), F. pasteurellaceae (family), O. enterobacterales (order)* were significantly enriched in the control group, specifically *S*. *parvula and S. parainfluenzae (species).* The differences (beta diversity) among the smokers and healthy controls microbial communities were statistically significant (*p* = 0.01).

### 3.4. Waterpipe smoking was associated with changes in oral microbial metabolism

**Figure 4:**
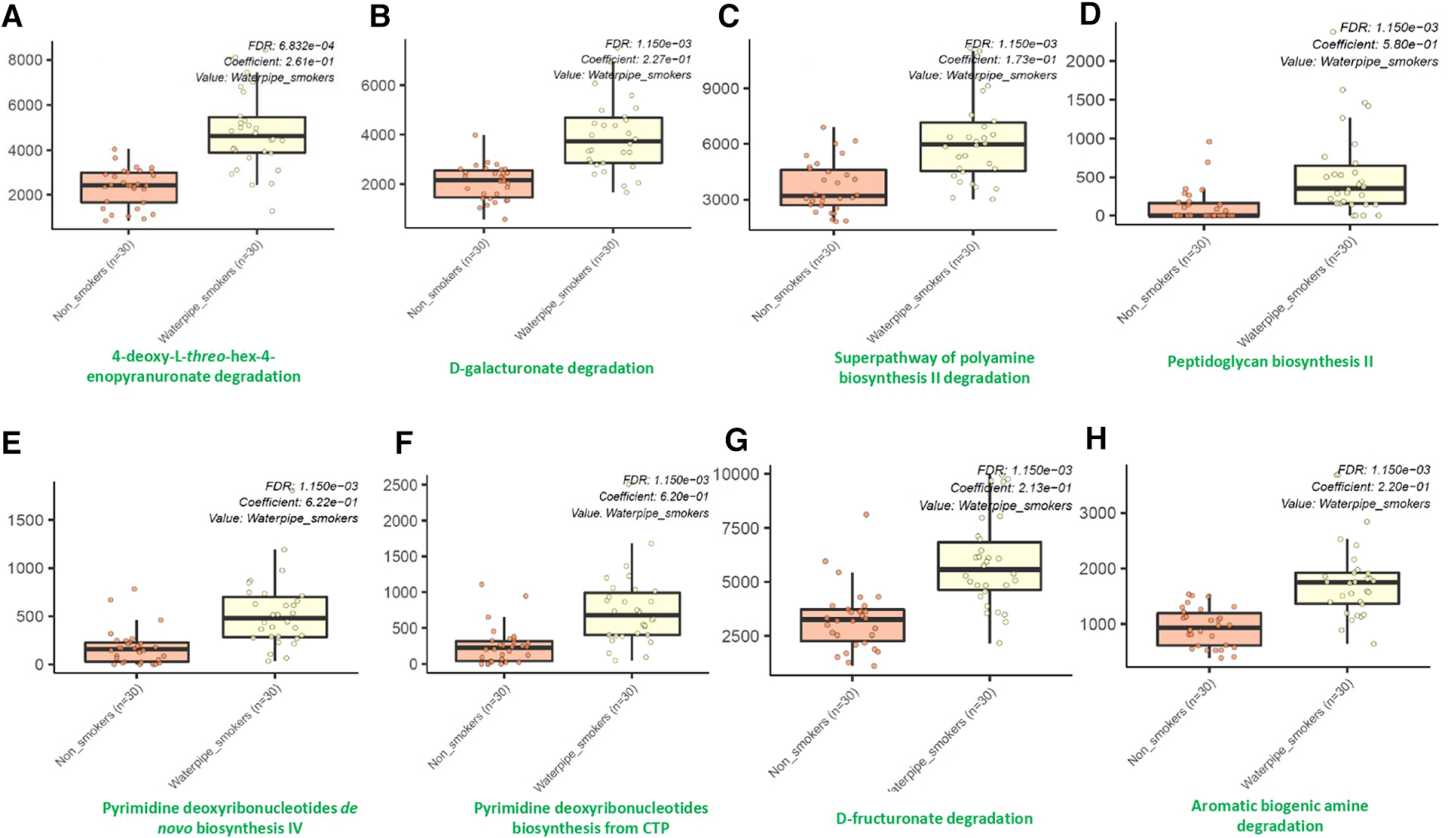
Differentially abundant metabolic pathways between smokers and non-smokers by MaAsLin2.

Using the MetaCyc pathway abundances, we observed significant differences in the abundances of thirty-seven different microbial metabolic pathways between waterpipe smokers and non-smokers. Among them, all were identified to be significantly higher in the waterpipe smokers group except for three pathways (Phoslipsyn pathway, fasyn long pathway, tetrapyrrole biosynthesis I pathway). The eight major pathways which showed the maximum differences between both groups are shown in Figure 4. Detailed information for all the pathways are provided in the Supplementary Material.

## 4. Discussion

The oral cavity is a highly complex ecological system with a dynamic relationship between the host and the oral microbiome (5). The functions of the microbial communities are a major determining factor of homeostasis that could potentially lead to dysbiosis (1). Tobacco smoke exposure is known to induce physiological and anatomical changes in the oral cavity, consequently altering the composition of the bacterial biofilms and the structure of the oral microbiome (1). However, there is still a paucity of information regarding the oral health effects of other forms of smoking. Waterpipe smoking has been around since the 15th century and is a popularly used form of tobacco smoking worldwide. However, little is known about the effects on the oral ecosystem and microbiome. To the best of our knowledge, this study provides some of the earliest experimental evidence of waterpipe smoking on the salivary microbiome and, thus, the rationale to further explore the potential mechanisms that underlie this shift.

We detected a significant taxonomic difference between the smokers and non-smokers and a lack of difference in microbial richness (alpha diversity). Our observation is perhaps unsurprising, as waterpipe smoking is known to be a definitive risk factor for oral infections and diseases. A few cigarette smoking studies reported a lack of difference in alpha diversity; this difference could be attributed to the type of tobacco used, sample size, and geographical heterogeneity (25–27). Our result showed that a significantly higher abundance of *Firmicutes* and *Streptococcus* is a feature of the waterpipe smoker, consistent with existing literature on cigarette smoking (26–28). These results were also consistent with a study performed with supragingival plaque samples of waterpipe smokers, where Al-Marzooq et al. found that phyla *Firmicutes* was the most abundant and abundance of *Proteobacteria* and *Actinobacteria* were significantly higher in waterpipe smokers. It was also found that *Bacteroidota* were significantly more common in non-smokers (28). However, in our study, though *Firmicutes* was the most abundant phylum, *Proteobacteria* was depleted, and *Bacteroidota* showed a minor increase in abundance in waterpipe smokers. In another study conducted in the UAE, genera *Porphyromona*s, *Veillonella,* and *Prevotella* were significantly less abundant in sub-gingival samples of waterpipe smokers; in contrast, we found a minor increase in the relative abundance of these species in our waterpipe smoker group (29). Valles et al. claim that a relative abundance of phyla *Cyanobacteria* and *SR1* was depleted in waterpipe smokers, whereas we found that *Proteobacteria, Haemophilus, and Lautropia* were depleted in smokers (10). This variation in results could potentially be due to the differences in sample type between these studies (30). For instance, our sample being salivary, may reflect the bacteria shed from the total oral cavity. In contrast, supra and subgingival plaque sampling would be a deeper representation of the gingival microbiome, which could be significantly affected by the periodontal status of the individual (31). Hence, these results highlight that it wouldn't be rational to assume the impacts of waterpipe smoking is similar across all microenvironments, and it may vary according to the individual niches (1, 32).

Few oral microbes falling into these differentially abundant phyla are known to be a common cause of human respiratory diseases and infections (13). Microbiota settling down in the oral micro-ecosystem is the primary source of the lung microbiome and has been linked with the development of respiratory diseases. For instance, *Firmicutes* and *Proteobacteria* were found in the respiratory microbiota of tuberculosis patients (33). *Lactobacillales* belonging to phylum *Firmicutes*, abundant in smokers, are one of the known risk factors for pleuro-pulmonary infections (34). In addition, *Streptococci* and anaerobes *Prevotella* and *Veillonella* have been associated with pneumonia infections, while tracheal aspirate specimens in chronic obstructive pulmonary disease patients show an abundance of *P. gingivalis* (35). Relative to lung cancer specifically, a greater abundance of *Bacilli* class and *Lactobacillales* order in saliva was associated with an increased risk (36).

Notably, most of the bacteria that showed increase in waterpipe smokers were facultative anaerobes, with aerobes showing a decline. This could be related to the deprivation of oral oxygen due to waterpipe smoking. Waterpipe smoking may create a depletion of an oxygen environment in the mouth and would reflect on the oxygen availability of microbes in the oral cavity, leading to alteration of the oral microbial ecology. It is established that decreased local oxygen tension caused by cigarette smoking promotes periodontal pathogens, leading to the subsequent development of periodontitis (37). Further, *S. oralis* enriched in our smokers' group is known to be a plaque-forming bacterium and can form a cohesive interaction with periodontal-pathogenic bacteria *P. gingivalis* due to Glyceraldehyde-3-Phosphate Dehydrogenase of *S. oralis* and thus can act as a bridge for colonization (38). *S. oralis* has been known to occasionally cause opportunistic infections such as bacteremia and bacterial endocarditis by cytotoxicity and enhancing the tissue-damaging effects of streptococcal H_2_O_2_. *S. oralis* and H_2_O_2_ damage the lysosomes by reducing the acidic environment, which is linked with the death process of macrophages; hence, it is implicated as an agent causing alteration in host immune responses (39). *S. salivarius*, also predominant in our smokers group, is known to cause nosocomial or iatrogenic central nervous system infections. *S. salivarius* has been detected in almost 60% of bacterial meningitis cases. Several reports describe *S. salivarius* to complicate upper respiratory tract infections, endocarditis, and neurosurgical procedures (40).

Another significant finding is the depletion of phylum *Proteobacteria* in the waterpipe smoker group, which is a consistent characteristic finding in periodontitis as well (41). Further, levels of *Proteobacteria* in the oral microbiome have been associated with insulin resistance and inflammation (42). A lower abundance of *Haemophilus,* as seen in our smoker population, has also been reported in smoker patients with rheumatoid arthritis and also in patients with oral lichen planus compared to healthy controls (43, 44). Though *Haemophilus* has been implicated in chronic inflammatory disease like chronic obstructive pulmonary disorder (COPD), it has a paradoxical impact on the gut microbiome as increased levels of *Haemophilus* enhances gut symbiosis and hence has a shown to have a protective effect against CRC (45). This paradoxical effect has been linked to NLRP3 inflammasome (45), and thus it is plausible that lower levels of *Haemophilus* could have unfavourable effects on oral microbiome. Furthermore, species that are part of the normal healthy flora, such as *Lautropia*, were also similarly found to be lower in cigarette smokers with moderate or severe periodontitis than those without disease (29).

The oral cavity is the first contact with smoke and may play an essential role in degrading toxic compounds. A key observation from published literature is that there is enriched degradation of polycyclic aromatic hydrocarbons and other constituents in the oral microbiome of cigarette smokers (46). We also observed that several microbial pathways related to the degradation of compounds were enriched in our cohort of waterpipe smokers. Waterpipe smoking generates several polycyclic aromatic hydrocarbons, carbon monoxide, and a high fraction of tiny particles that may adversely affect human health upon inhalation (47). Hence, an increase in these pathways is relatable. Furthermore, we also found an increase in polyamine synthesis pathways in our waterpipe smokers. Polyamines and their metabolites are often regarded as cancer biomarkers, and multiple malignancies have been linked to polyamine imbalance (48). For instance, cell proliferation and death in breast cancer are significantly influenced by polyamine metabolism. Additionally, there is proof that polyamines aid in the interactions of transcription factors with their particular response elements, including nuclear factor-kB, c MYC, and other receptors (48–50). Another vital pathway significantly upregulated in waterpipe smokers is the peptidoglycan biosynthesis pathway. Peptidoglycans are critical structural components for bacteria that are indispensable for virulence (51, 52). Our results only offer a snapshot of oral microbial metabolome alteration in waterpipe users. Further work is required to explore the detailed mechanism of this dysbiosis and its mechanistic implication in oral health and disease.

Our study has a few limitations. Certain confounding factors that may affect oral microbiome, including diet, alcohol consumption, oral hygiene, and systemic diseases, could not be controlled in this study due to technical hitches of field study design. Further, the oral health status of participants was self-reported and hence may not reflect the actual periodontal health status. However, as study participants were younger, it was generally not expected to significantly impact the results. Further, our samples were not subgingival or supragingival plaque, which could have a direct impact on periodontal disease; our samples being saliva, is composed of shedded microbiome from all oral surfaces. Due to the majority of younger participants, our findings are not generalizable to the older population since the microbiome varies with age. In addition, 16s rRNA sequencing provides phylogenetic information to identify the isolate down to the genus level and, in some cases, up to the species level. However, 16s rRNA sequencing remains the most accepted and popular method for studying the microbiome (51). Alternatively, further studies using metagenomic sequencing techniques would capture higher levels of diversity as its high sensitivity allows the delivery of knowledge on the taxonomic composition and the functional genes in a sample. Mainly it would detect more phyla and low abundance genera compared to 16 s rRNA sequencing (51). Despite the limitations mentioned above, our study still describes the impact of waterpipe on the oral microbiome to a great extent. Due to our research's cross-sectional nature, it is impossible to directly assess the temporal link between smoking-related exposures and oral microbial outcomes. However, seems doubtful that modification of the oral microbiome precedes smoking, as smoking is a behaviour and the oral microbiome is an observed state. Nevertheless, a longitudinal study would allow us to observe waterpipe-related tobacco exposure changes.

## 5. Conclusions

This study provides preliminary evidence on the effect of waterpipe smoking on the composition and metabolic alterations of salivary microbiota. Understanding the possible effects of waterpipe on the oral microbiota and, ultimately human health is crucial as more teenagers and young people start using waterpipe and chronically expose their oral cavity and airways to waterpipe smoke. Despite having a small sample size, this study identified that waterpipe use results in dysbiosis of the oral commensal microbial communities with notable differences in composition and metabolic functions. Further studies with larger samples need to be explored to validate these findings.

## Data Availability

The datasets presented in this study can be found in online repositories. The names of the repository/repositories and accession number(s) can be found below: https://www.ncbi.nlm.nih.gov/genbank/, BioProject ID PRJNA940686.
