# Supplementary figures and images for: Impact of waterpipe smoking on the salivary microbiome
Source: Front Oral Health. 2023 Nov 9;4:1275717. doi: 10.3389/froh.2023.1275717 (PMC10665852; doi:10.3389/froh.2023.1275717)

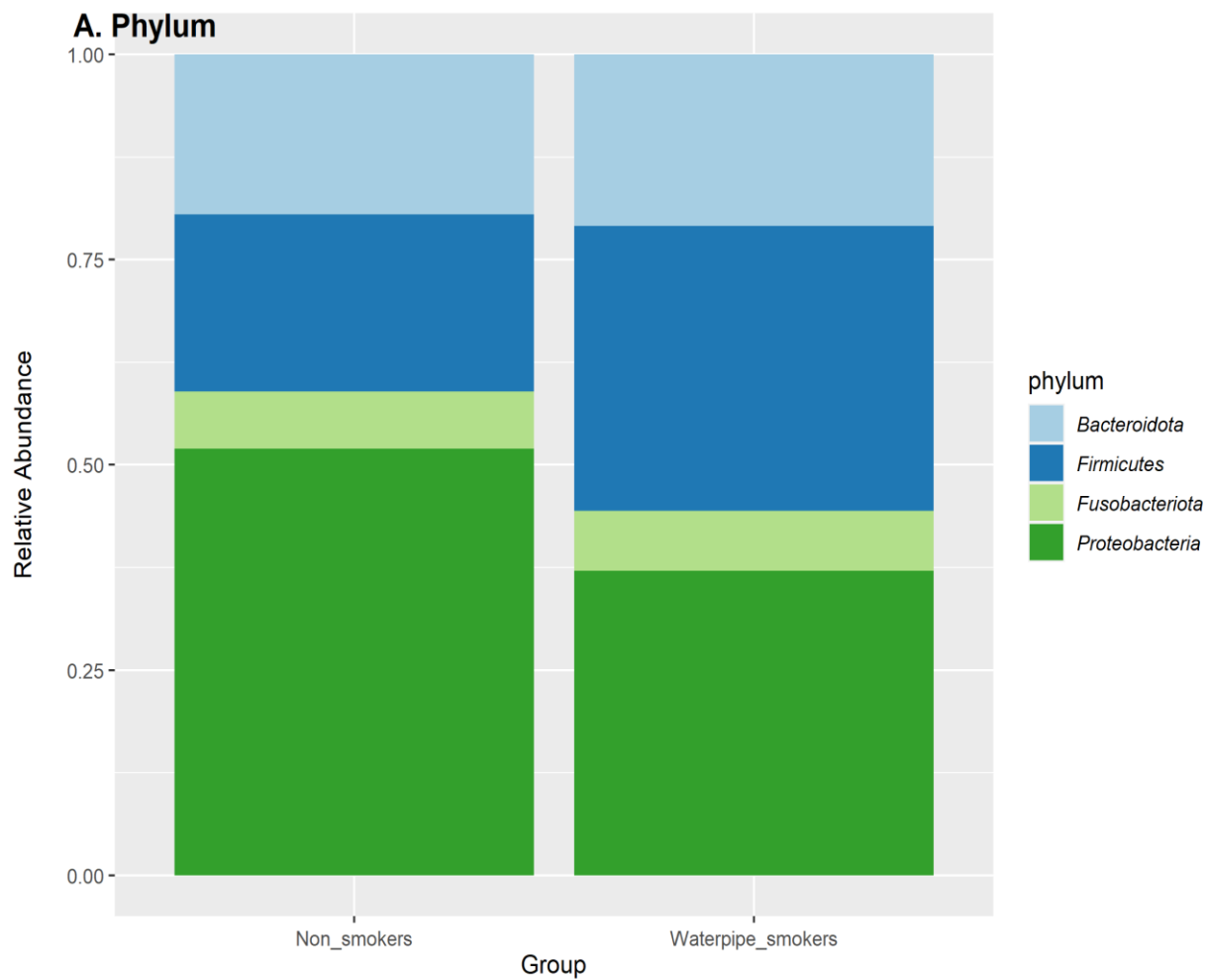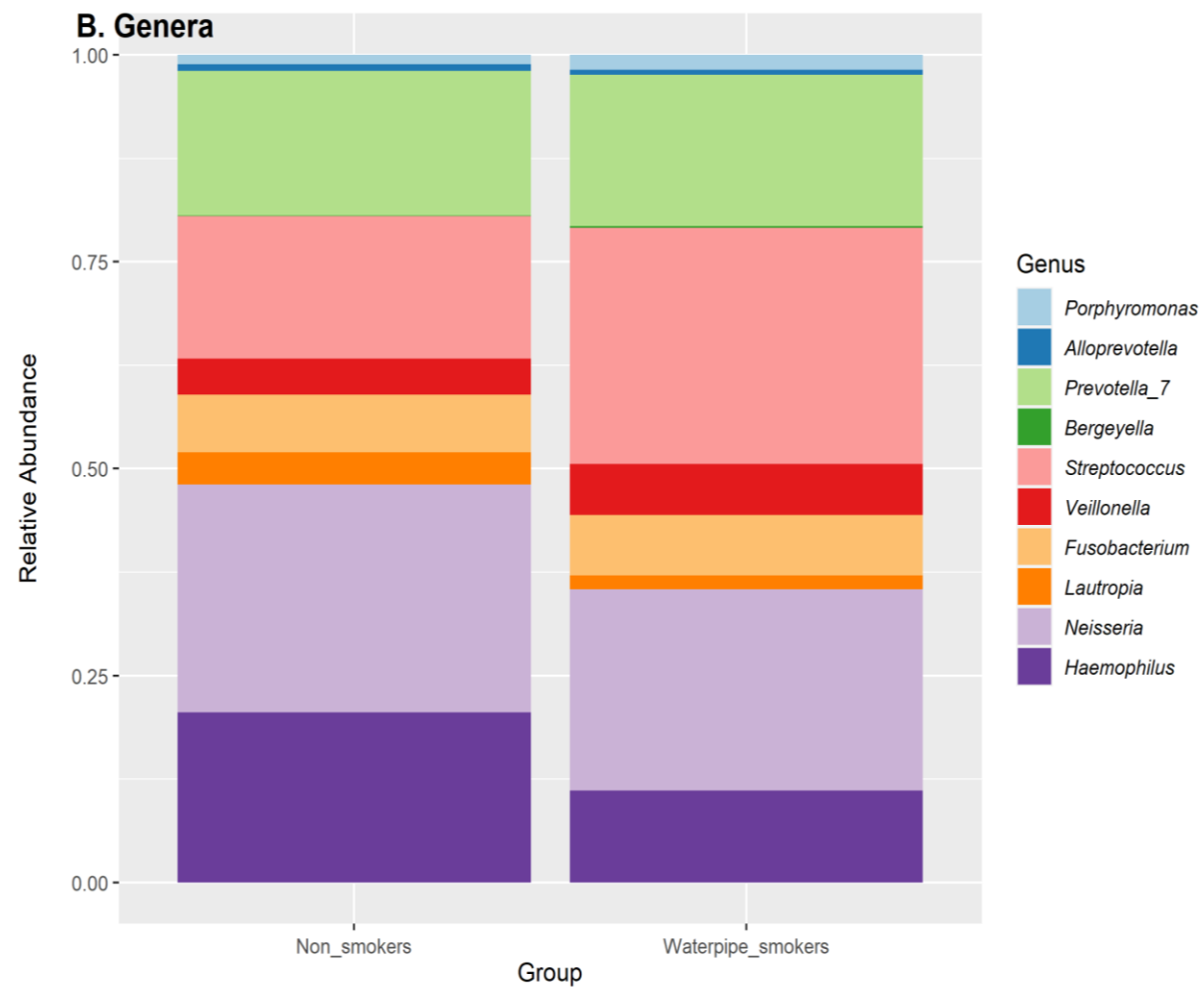

Supplement: Supplementary file 1 [file Datasheet1.pdf]
