# Supplementary material for: Impact of waterpipe smoking on the salivary microbiome
Source: Front Oral Health. 2023 Nov 9;4:1275717. doi: 10.3389/froh.2023.1275717 (PMC10665852; doi:10.3389/froh.2023.1275717)
Supplement: Supplementary file 2 [file Datasheet2.pdf]

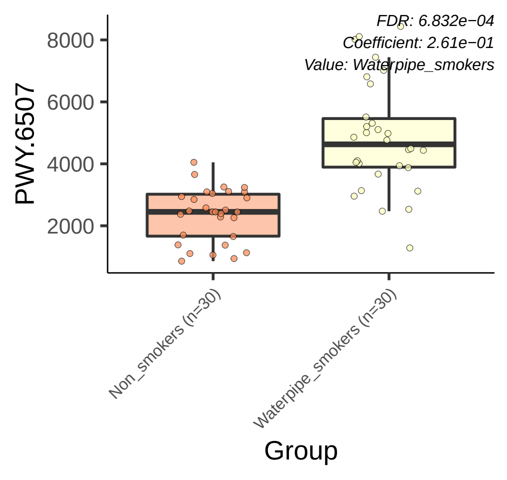

FDR: 6.832e−04
Coefficient: 2.61e−01
Value: Waterpipe_smokers
PWY.6507
8000
6000
4000
2000
Non_smokers (n=30)
Waterpipe_smokers (n=30)
Group

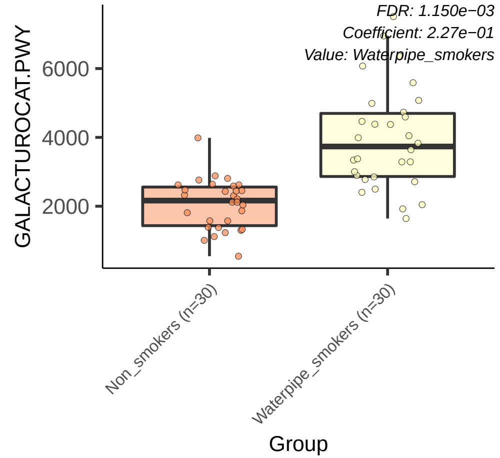
FDR: 1.150e−03
Coefficient: 2.27e−01
Value: Waterpipe_smokers
GALACTUROCAT.PWY
6000
4000
2000
Non_smokers (n=30)
Waterpipe_smokers (n=30)
Group

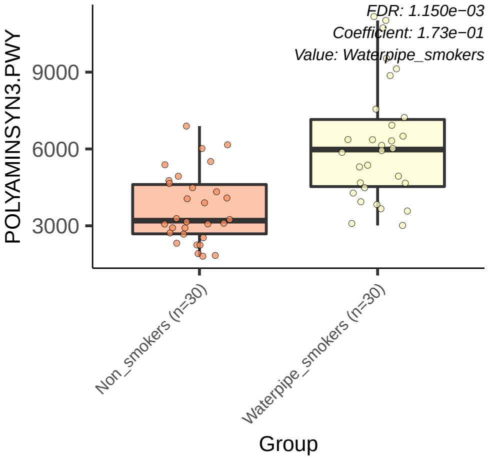
FDR: 1.150e−03
Coefficient: 1.73e−01
Value: Waterpipe_smokers
POLYAMINSYN3.PWY
9000
6000
3000
Non_smokers (n=30)
Waterpipe_smokers (n=30)
Group

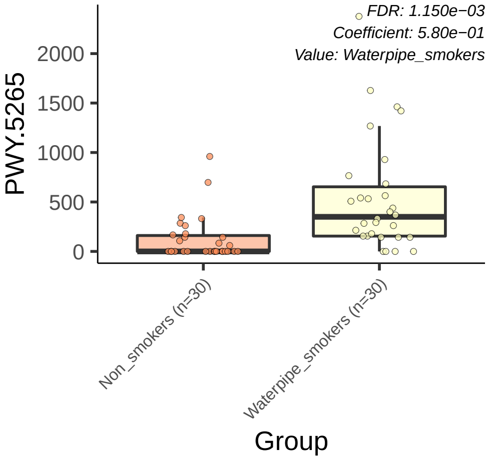

FDR: 1.150e−03
Coefficient: 5.80e−01
Value: Waterpipe_smokers
PWY.5265
2000
1500
1000
500
0
Non_smokers (n=30)
Waterpipe_smokers (n=30)
Group

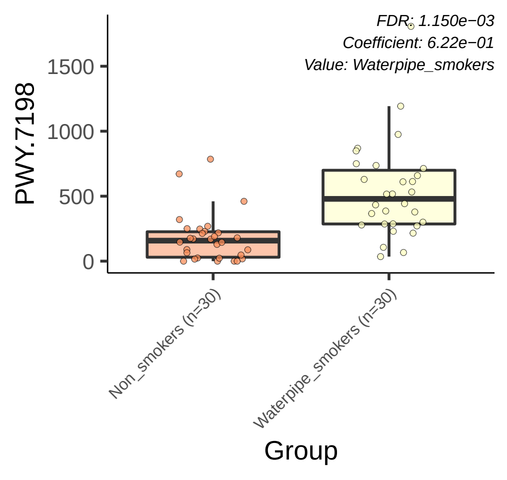

FDR: 1.150e−03
Coefficient: 6.22e−01
Value: Waterpipe_smokers
PWY.7198
1500
1000
500
0
Non_smokers (n=30)
Waterpipe_smokers (n=30)
Group

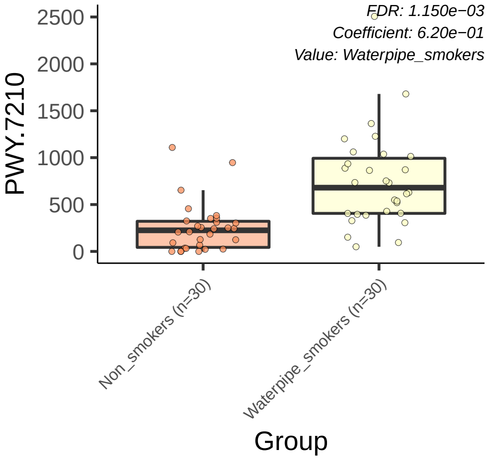

FDR: 1.150e−03
Coefficient: 6.20e−01
Value: Waterpipe_smokers
PWY.7210
2500
2000
1500
1000
500
0
Non_smokers (n=30)
Waterpipe_smokers (n=30)
Group

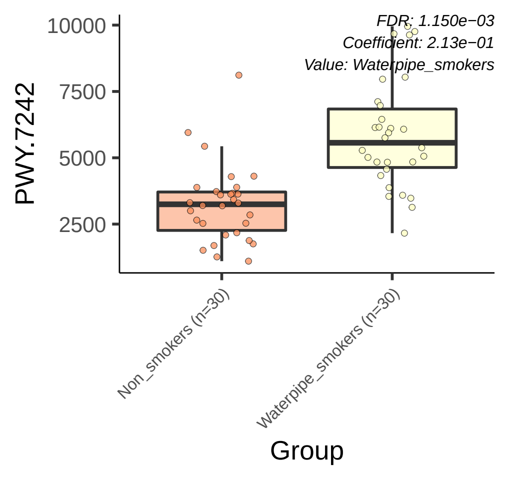

FDR: 1.150e−03
Coefficient: 2.13e−01
Value: Waterpipe_smokers
PWY.7242
10000
7500
5000
2500
Non_smokers (n=30)
Waterpipe_smokers (n=30)
Group

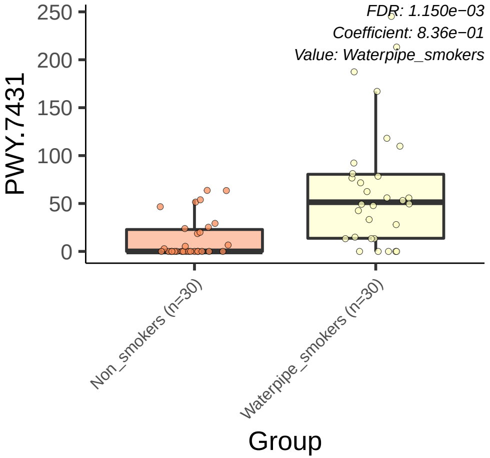

FDR: 1.150e−03
Coefficient: 8.36e−01
Value: Waterpipe_smokers
PWY.7431
250
200
150
100
50
0
Non_smokers (n=30)
Waterpipe_smokers (n=30)
Group

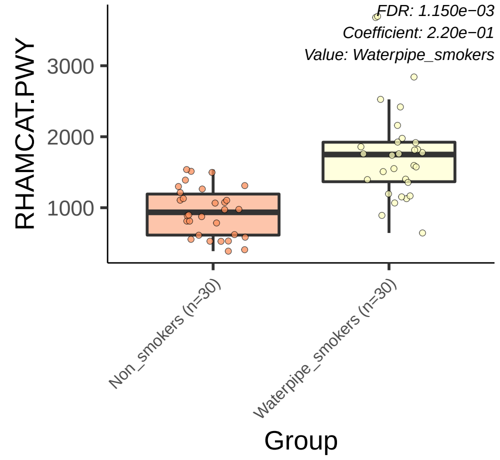

FDR: 1.150e−03
Coefficient: 2.20e−01
Value: Waterpipe_smokers
RHAMCAT.PWY
3000
2000
1000
Non_smokers (n=30)
Waterpipe_smokers (n=30)
Group

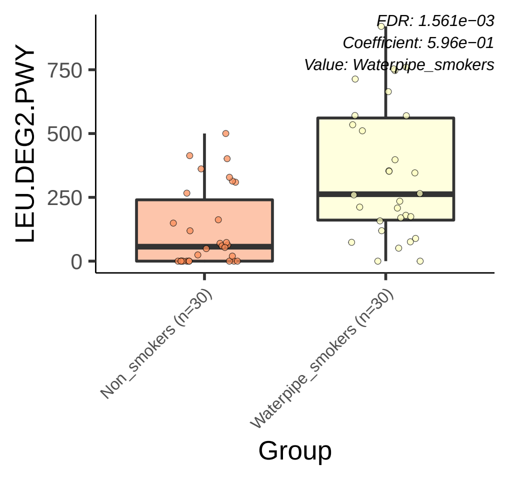

FDR: 1.561e−03
Coefficient: 5.96e−01
Value: Waterpipe_smokers
LEU.DEG2.PWY
750
500
250
0
Non_smokers (n=30)
Waterpipe_smokers (n=30)
Group

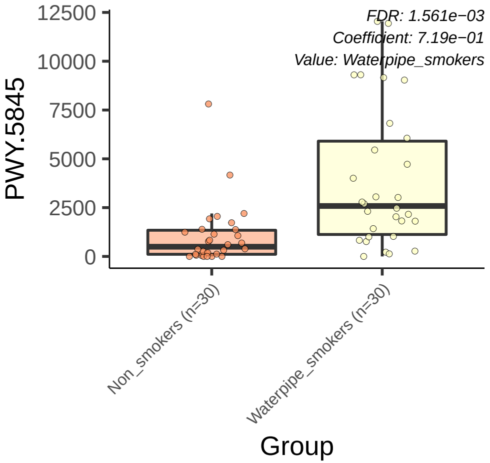

FDR: 1.561e−03
Coefficient: 7.19e−01
Value: Waterpipe_smokers
PWY.5845
12500
10000
7500
5000
2500
0
Non_smokers (n=30)
Waterpipe_smokers (n=30)
Group

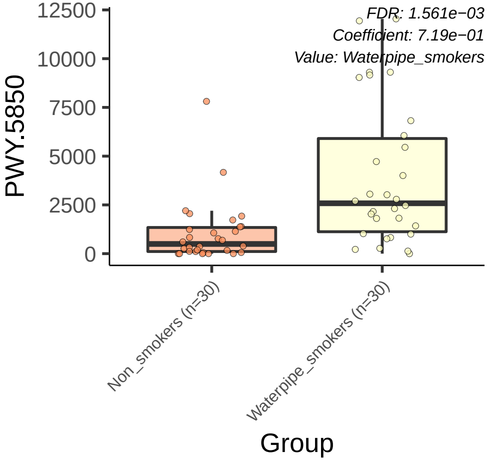

FDR: 1.561e−03
Coefficient: 7.19e−01
Value: Waterpipe_smokers
PWY.5850
12500
10000
7500
5000
2500
0
Non_smokers (n=30)
Waterpipe_smokers (n=30)
Group

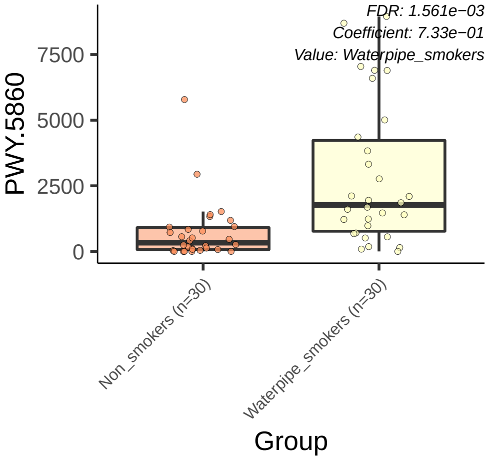

FDR: 1.561e−03
Coefficient: 7.33e−01
Value: Waterpipe_smokers
PWY.5860
7500
5000
2500
0
Non_smokers (n=30)
Waterpipe_smokers (n=30)
Group

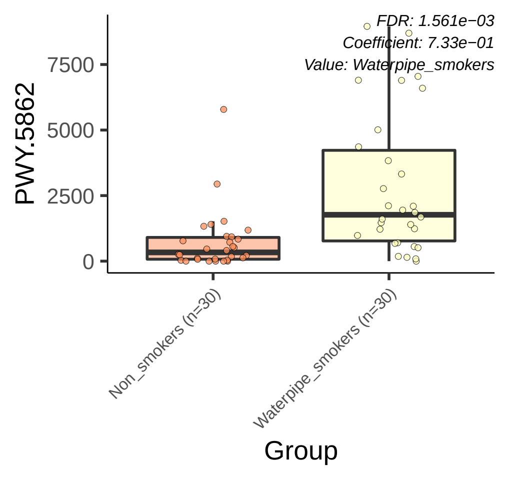

FDR: 1.561e−03
Coefficient: 7.33e−01
Value: Waterpipe_smokers
PWY.5862
7500
5000
2500
0
Non_smokers (n=30)
Waterpipe_smokers (n=30)
Group

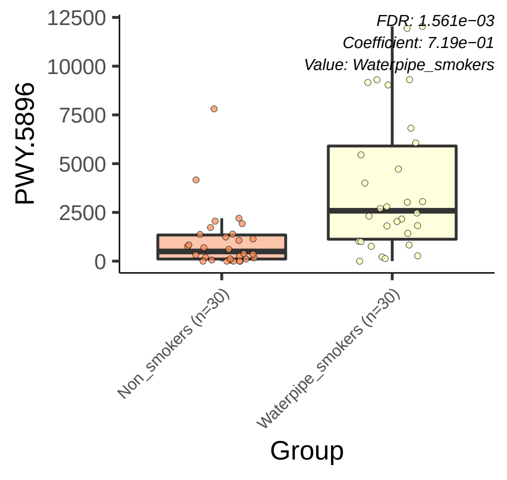

FDR: 1.561e−03
Coefficient: 7.19e−01
Value: Waterpipe_smokers
PWY.5896
12500
10000
7500
5000
2500
0
Non_smokers (n=30)
Waterpipe_smokers (n=30)
Group

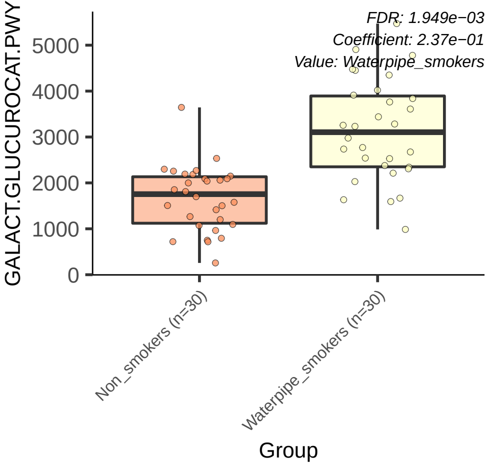

FDR: 1.949e−03
Coefficient: 2.37e−01
Value: Waterpipe_smokers
GALACT.GLUCUROCAT.PWY
0
1000
2000
3000
4000
5000
Non_smokers (n=30)
Waterpipe_smokers (n=30)
Group

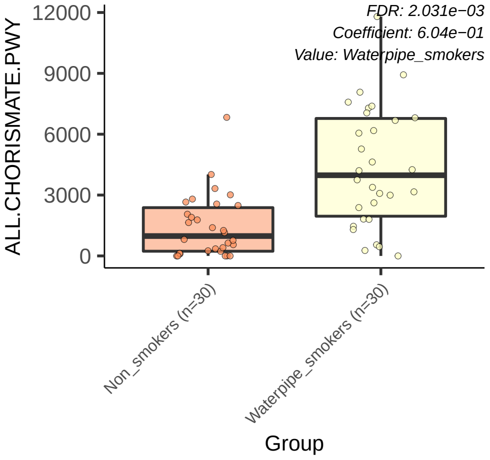

FDR: 2.031e−03
Coefficient: 6.04e−01
Value: Waterpipe_smokers
ALL.CHORISMATE.PWY
12000
9000
6000
3000
0
Non_smokers (n=30)
Waterpipe_smokers (n=30)
Group

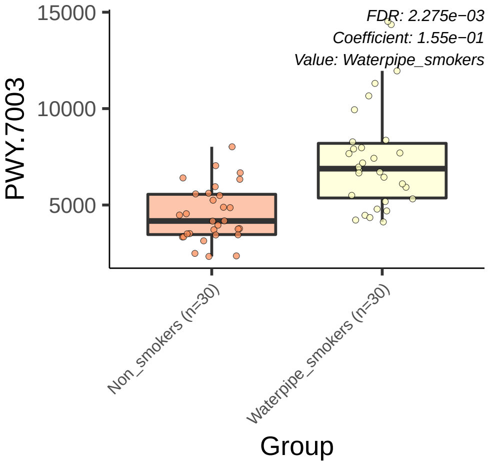
FDR: 2.275e−03
Coefficient: 1.55e−01
Value: Waterpipe_smokers
PWY.7003
15000
10000
5000
Non_smokers (n=30)
Waterpipe_smokers (n=30)
Group

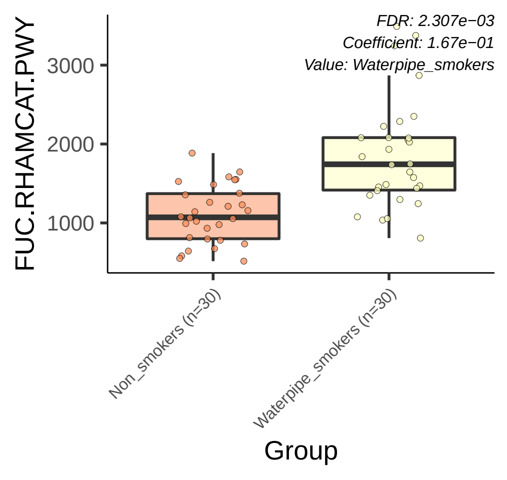
FDR: 2.307e−03
Coefficient: 1.67e−01
Value: Waterpipe_smokers
FUC.RHAMCAT.PWY
3000
2000
1000
Non_smokers (n=30)
Waterpipe_smokers (n=30)
Group

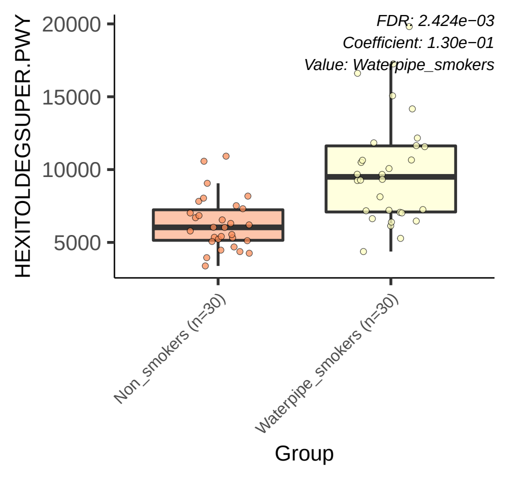

FDR: 2.424e−03
Coefficient: 1.30e−01
Value: Waterpipe_smokers
HEXITOLDEGSUPER.PWY
20000
15000
10000
5000
Non_smokers (n=30)
Waterpipe_smokers (n=30)
Group

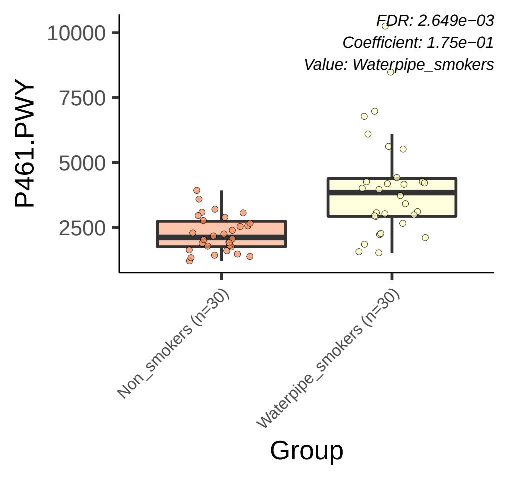

FDR: 2.649e−03
Coefficient: 1.75e−01
Value: Waterpipe_smokers
P461.PWY
10000
7500
5000
2500
Non_smokers (n=30)
Waterpipe_smokers (n=30)
Group

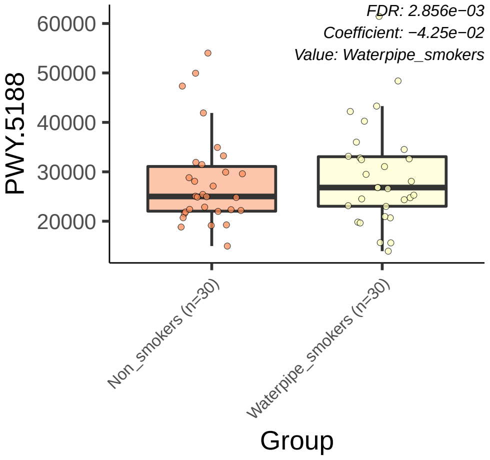

FDR: 2.856e−03
Coefficient: −4.25e−02
Value: Waterpipe_smokers
PWY.5188
60000
50000
40000
30000
20000
Non_smokers (n=30)
Waterpipe_smokers (n=30)
Group

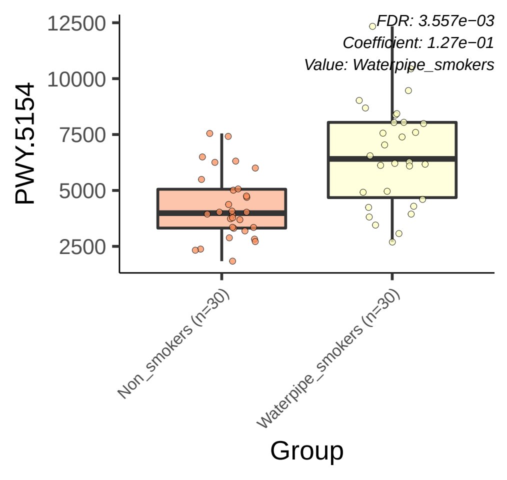
FDR: 3.557e−03
Coefficient: 1.27e−01
Value: Waterpipe_smokers
PWY.5154
12500
10000
7500
5000
2500
Non_smokers (n=30)
Waterpipe_smokers (n=30)
Group

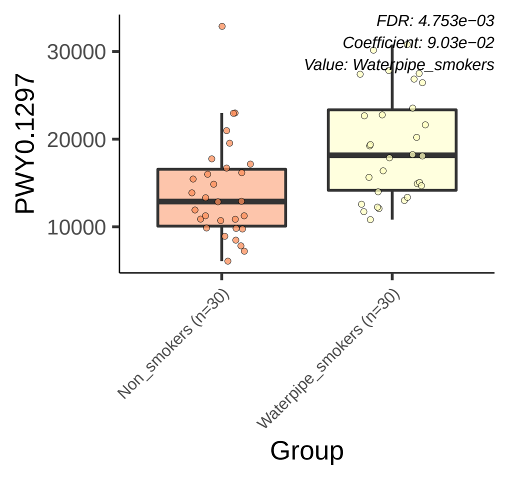
FDR: 4.753e−03
Coefficient: 9.03e−02
Value: Waterpipe_smokers
PWY0.1297
30000
20000
10000
Non_smokers (n=30)
Waterpipe_smokers (n=30)
Group

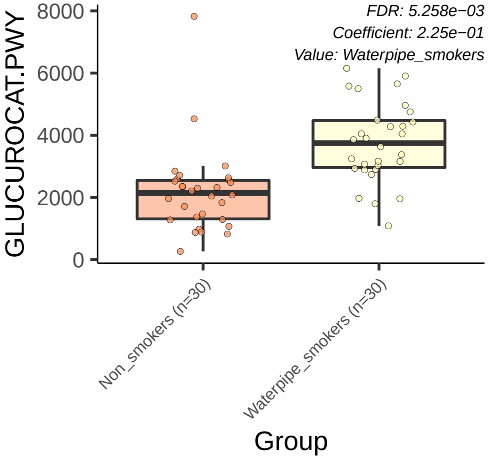

FDR: 5.258e−03
Coefficient: 2.25e−01
Value: Waterpipe_smokers
GLUCUROCAT.PWY
8000
6000
4000
2000
0
Non_smokers (n=30)
Waterpipe_smokers (n=30)
Group

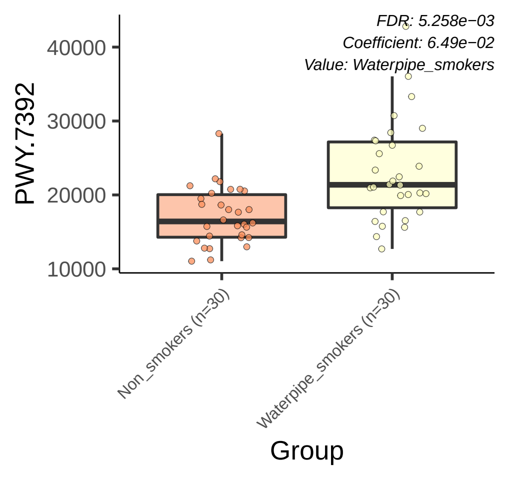

FDR: 5.258e−03
Coefficient: 6.49e−02
Value: Waterpipe_smokers
PWY.7392
40000
30000
20000
10000
Non_smokers (n=30)
Waterpipe_smokers (n=30)
Group

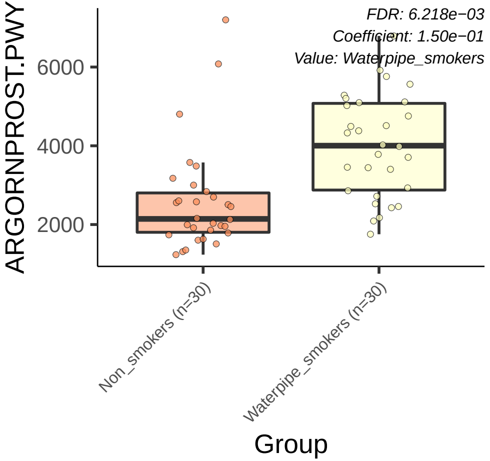

FDR: 6.218e−03
Coefficient: 1.50e−01
Value: Waterpipe_smokers
ARGORNPROST.PWY
6000
4000
2000
Non_smokers (n=30)
Waterpipe_smokers (n=30)
Group

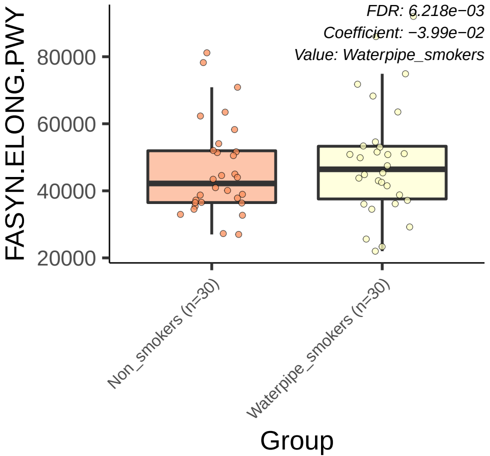

FDR: 6.218e−03
Coefficient: −3.99e−02
Value: Waterpipe_smokers
FASYN.ELONG.PWY
80000
60000
40000
20000
Non_smokers (n=30)
Waterpipe_smokers (n=30)
Group

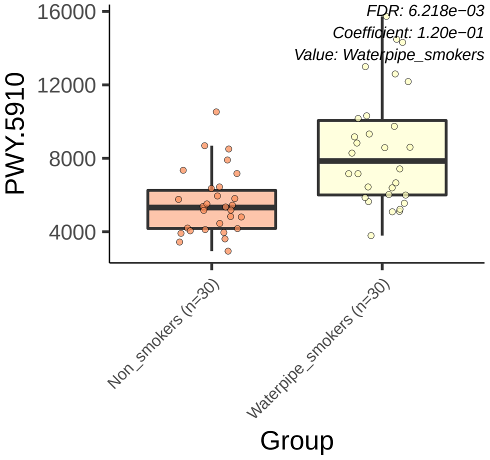

FDR: 6.218e−03
Coefficient: 1.20e−01
Value: Waterpipe_smokers
PWY.5910
16000
12000
8000
4000
Non_smokers (n=30)
Waterpipe_smokers (n=30)
Group

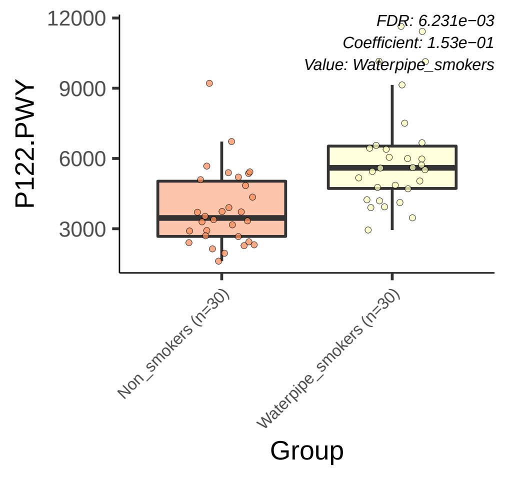
FDR: 6.231e−03
Coefficient: 1.53e−01
Value: Waterpipe_smokers
P122.PWY
12000
9000
6000
3000
Non_smokers (n=30)
Waterpipe_smokers (n=30)
Group

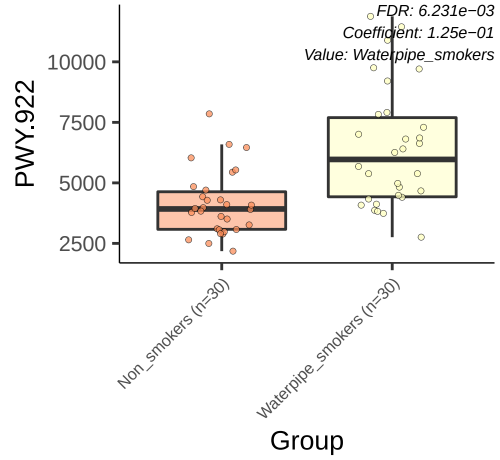

FDR: 6.231e−03
Coefficient: 1.25e−01
Value: Waterpipe_smokers
PWY.922
10000
7500
5000
2500
Non_smokers (n=30)
Waterpipe_smokers (n=30)
Group

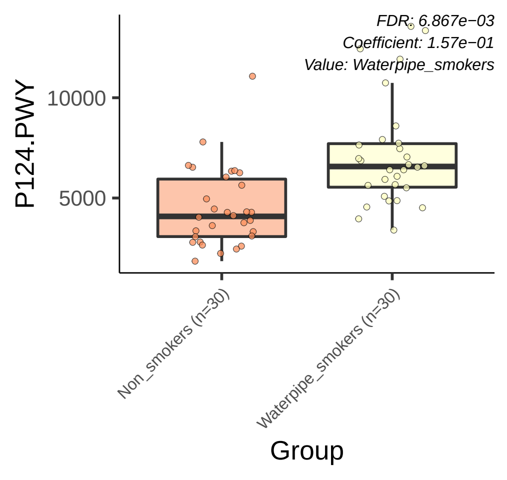
FDR: 6.867e−03
Coefficient: 1.57e−01
Value: Waterpipe_smokers
P124.PWY
10000
5000
Non_smokers (n=30)
Waterpipe_smokers (n=30)
Group

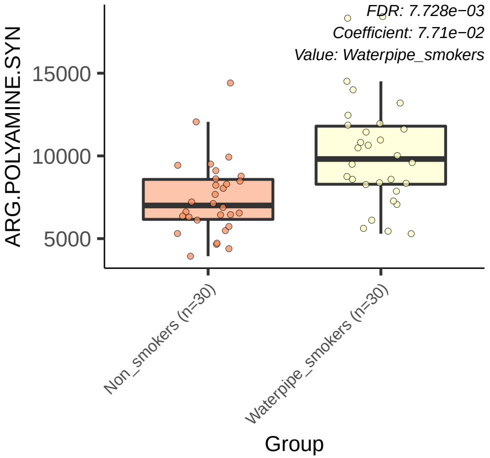
FDR: 7.728e−03
Coefficient: 7.71e−02
Value: Waterpipe_smokers
ARG.POLYAMINE.SYN
15000
10000
5000
Non_smokers (n=30)
Waterpipe_smokers (n=30)
Group

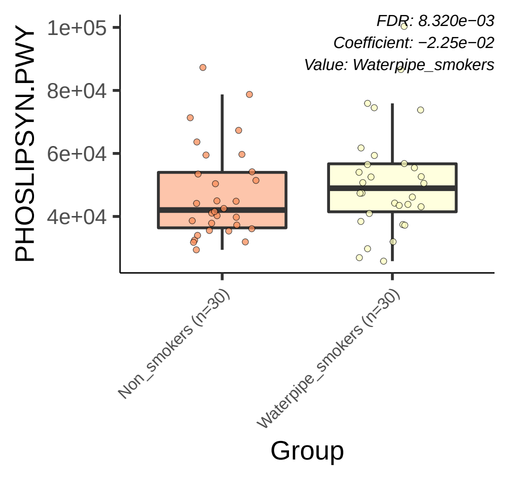

FDR: 8.320e−03
Coefficient: −2.25e−02
Value: Waterpipe_smokers
PHOSLIPSYN.PWY
1e+05
8e+04
6e+04
4e+04
Non_smokers (n=30)
Waterpipe_smokers (n=30)
Group

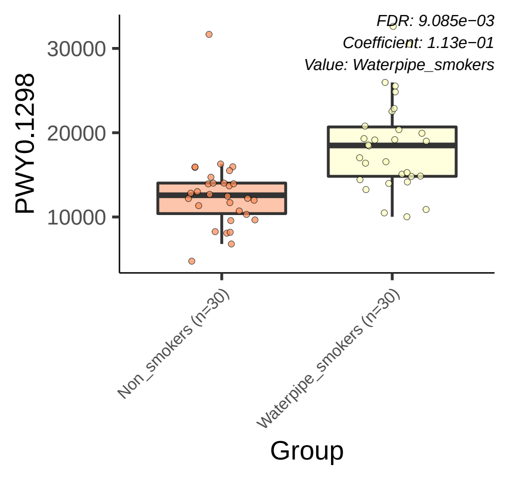
FDR: 9.085e−03
Coefficient: 1.13e−01
Value: Waterpipe_smokers
PWY0.1298
30000
20000
10000
Non_smokers (n=30)
Waterpipe_smokers (n=30)
Group

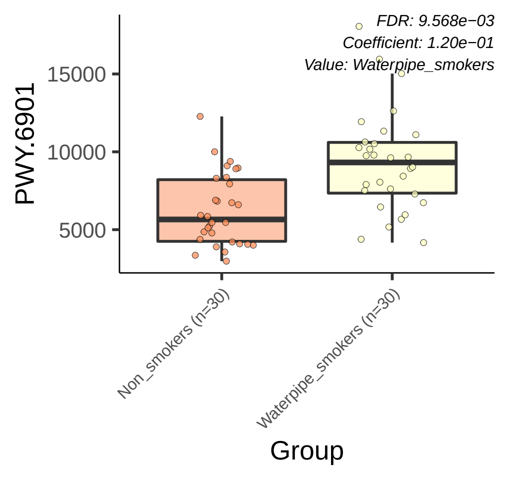
FDR: 9.568e−03
Coefficient: 1.20e−01
Value: Waterpipe_smokers
PWY.6901
15000
10000
5000
Non_smokers (n=30)
Waterpipe_smokers (n=30)
Group

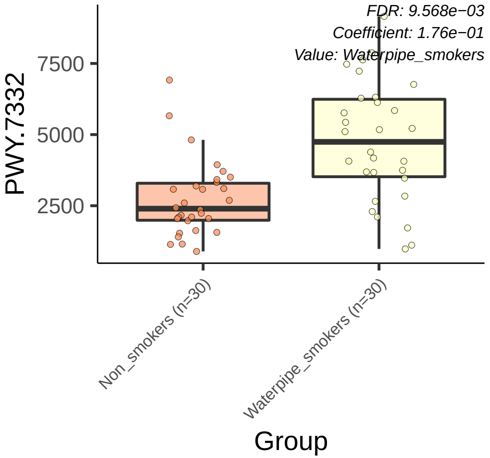
FDR: 9.568e−03
Coefficient: 1.76e−01
Value: Waterpipe_smokers
PWY.7332
7500
5000
2500
Non_smokers (n=30)
Waterpipe_smokers (n=30)
Group

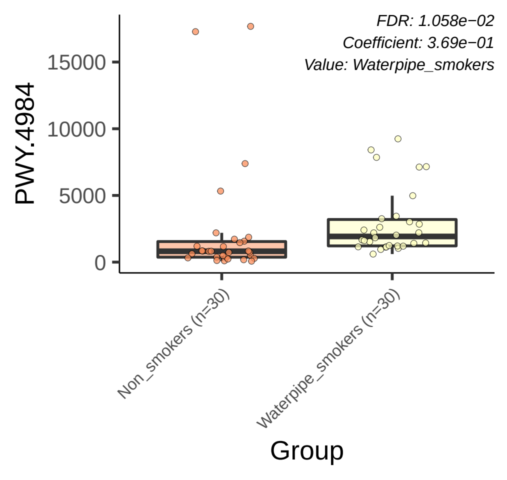
FDR: 1.058e−02
Coefficient: 3.69e−01
Value: Waterpipe_smokers
PWY.4984
15000
10000
5000
0
Non_smokers (n=30)
Waterpipe_smokers (n=30)
Group

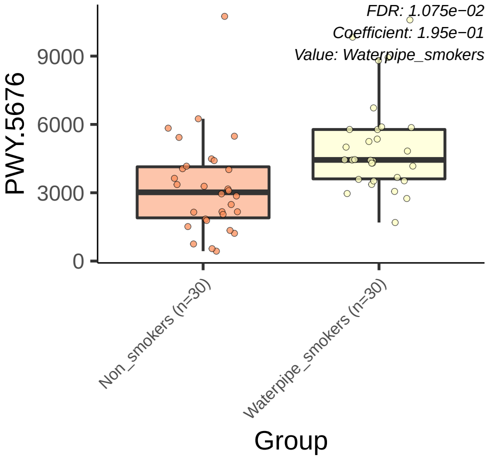

FDR: 1.075e−02
Coefficient: 1.95e−01
Value: Waterpipe_smokers
PWY.5676
9000
6000
3000
0
Non_smokers (n=30)
Waterpipe_smokers (n=30)
Group

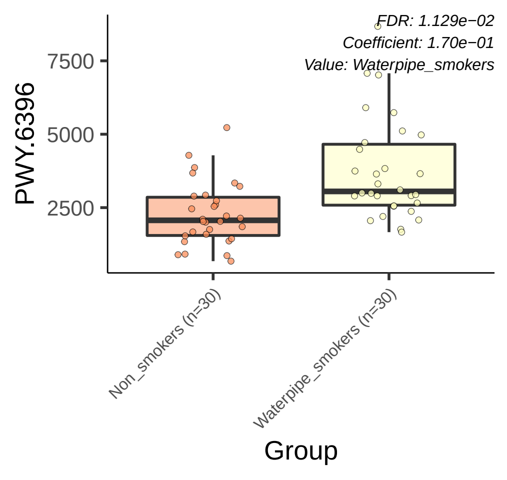
FDR: 1.129e−02
Coefficient: 1.70e−01
Value: Waterpipe_smokers
PWY.6396
7500
5000
2500
Non_smokers (n=30)
Waterpipe_smokers (n=30)
Group

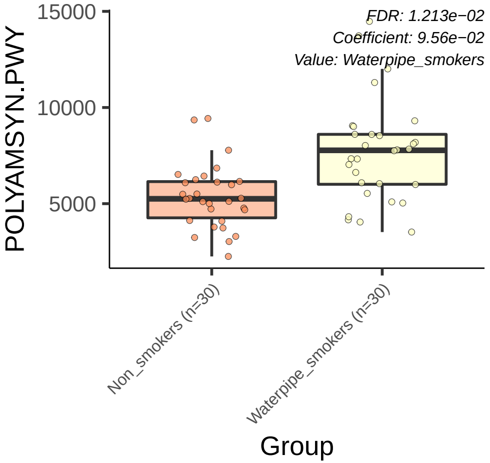

FDR: 1.213e−02
Coefficient: 9.56e−02
Value: Waterpipe_smokers
POLYAMSYN.PWY
15000
10000
5000
Non_smokers (n=30)
Waterpipe_smokers (n=30)
Group

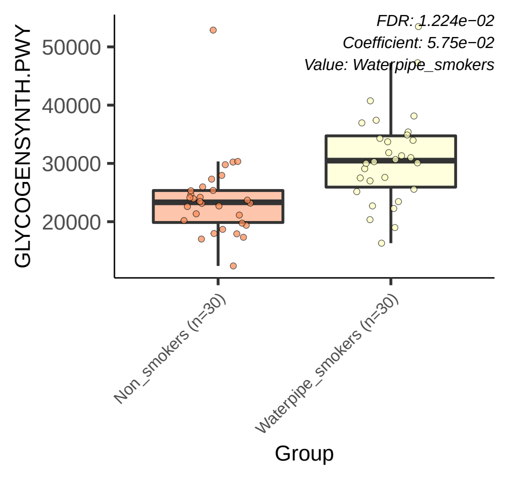
FDR: 1.224e−02
Coefficient: 5.75e−02
Value: Waterpipe_smokers
GLYCOGENSYNTH.PWY
50000
40000
30000
20000
Non_smokers (n=30)
Waterpipe_smokers (n=30)
Group

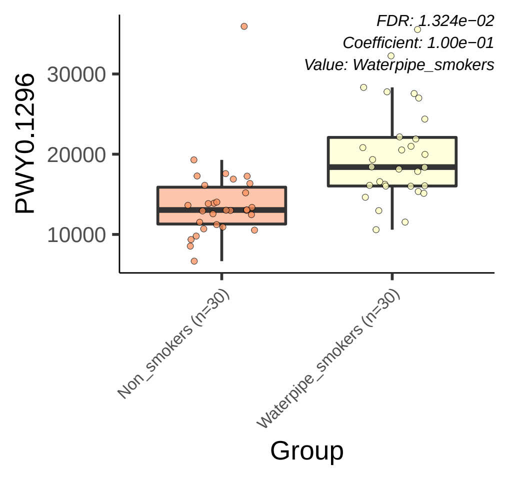
FDR: 1.324e−02
Coefficient: 1.00e−01
Value: Waterpipe_smokers
PWY0.1296
30000
20000
10000
Non_smokers (n=30)
Waterpipe_smokers (n=30)
Group

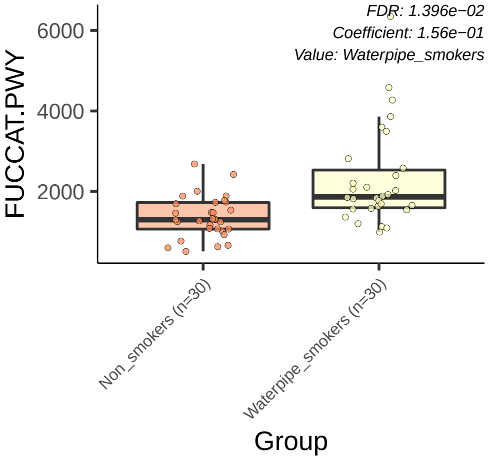
FDR: 1.396e−02
Coefficient: 1.56e−01
Value: Waterpipe_smokers
FUCCAT.PWY
6000
4000
2000
Non_smokers (n=30)
Waterpipe_smokers (n=30)
Group

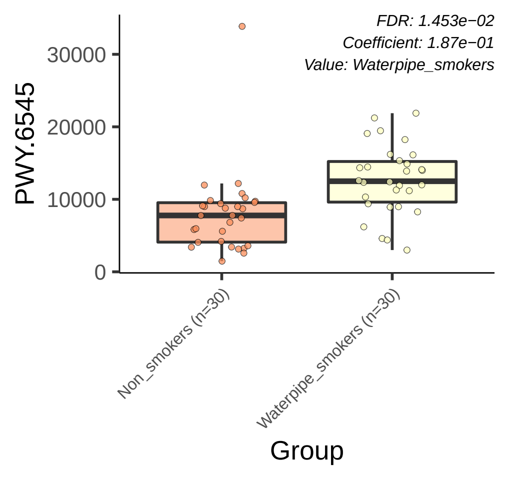
FDR: 1.453e−02
Coefficient: 1.87e−01
Value: Waterpipe_smokers
PWY.6545
30000
20000
10000
0
Non_smokers (n=30)
Waterpipe_smokers (n=30)
Group

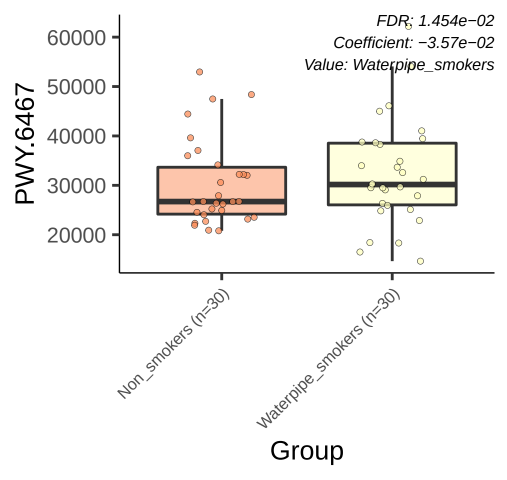

FDR: 1.454e−02
Coefficient: −3.57e−02
Value: Waterpipe_smokers
PWY.6467
60000
50000
40000
30000
20000
Non_smokers (n=30)
Waterpipe_smokers (n=30)
Group

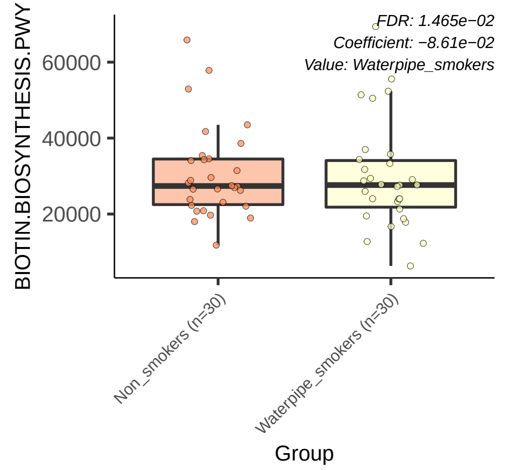

FDR: 1.465e−02
Coefficient: −8.61e−02
Value: Waterpipe_smokers
BIOTIN.BIOSYNTHESIS.PWY
60000
40000
20000
Non_smokers (n=30)
Waterpipe_smokers (n=30)
Group

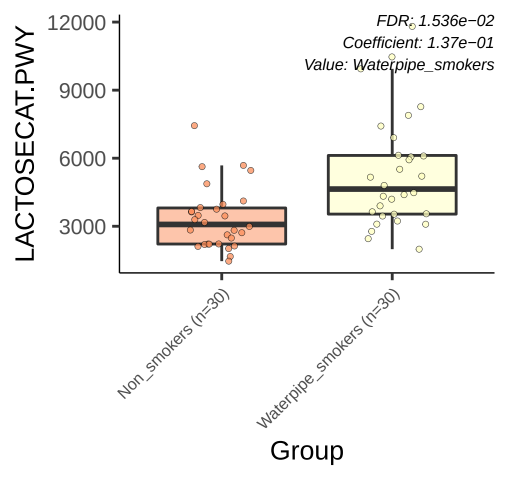
FDR: 1.536e−02
Coefficient: 1.37e−01
Value: Waterpipe_smokers
LACTOSECAT.PWY
12000
9000
6000
3000
Non_smokers (n=30)
Waterpipe_smokers (n=30)
Group

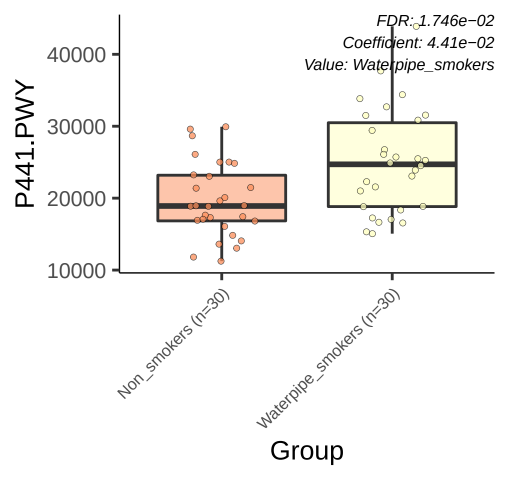
FDR: 1.746e−02
Coefficient: 4.41e−02
Value: Waterpipe_smokers
P441.PWY
40000
30000
20000
10000
Non_smokers (n=30)
Waterpipe_smokers (n=30)
Group

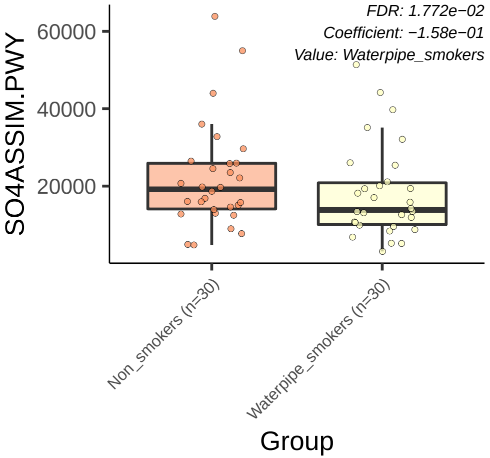
FDR: 1.772e−02
Coefficient: −1.58e−01
Value: Waterpipe_smokers
SO4ASSIM.PWY
60000
40000
20000
Non_smokers (n=30)
Waterpipe_smokers (n=30)
Group

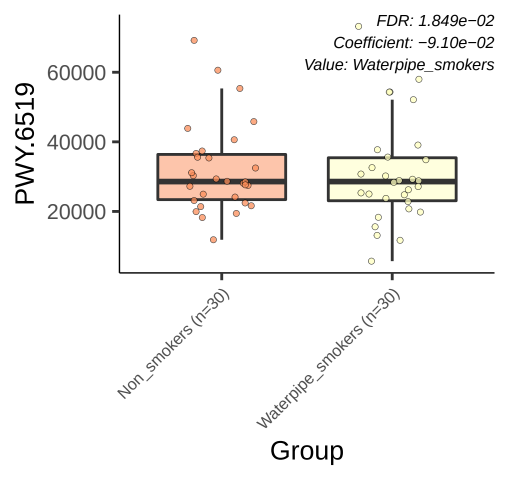

FDR: 1.849e−02
Coefficient: −9.10e−02
Value: Waterpipe_smokers
PWY.6519
60000
40000
20000
Non_smokers (n=30)
Waterpipe_smokers (n=30)
Group

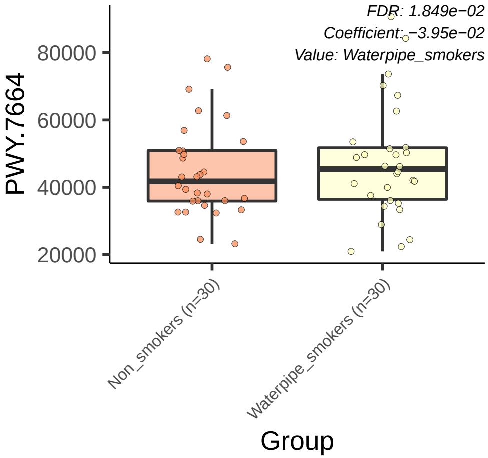

FDR: 1.849e−02
Coefficient: −3.95e−02
Value: Waterpipe_smokers
PWY.7664
80000
60000
40000
20000
Non_smokers (n=30)
Waterpipe_smokers (n=30)
Group

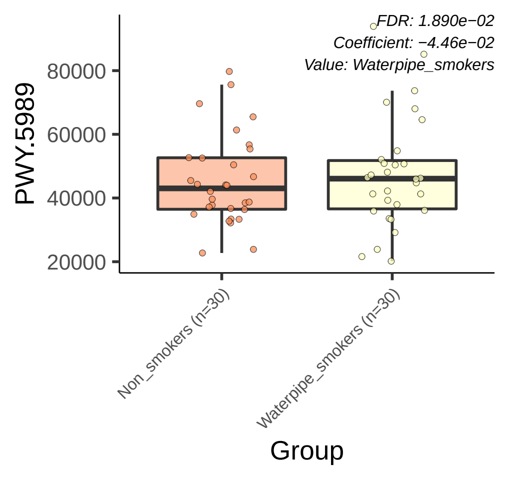

FDR: 1.890e−02
Coefficient: −4.46e−02
Value: Waterpipe_smokers
PWY.5989
80000
60000
40000
20000
Non_smokers (n=30)
Waterpipe_smokers (n=30)
Group

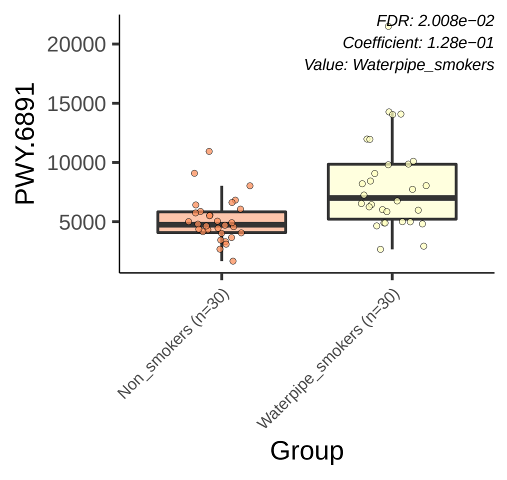

FDR: 2.008e−02
Coefficient: 1.28e−01
Value: Waterpipe_smokers
PWY.6891
20000
15000
10000
5000
Non_smokers (n=30)
Waterpipe_smokers (n=30)
Group

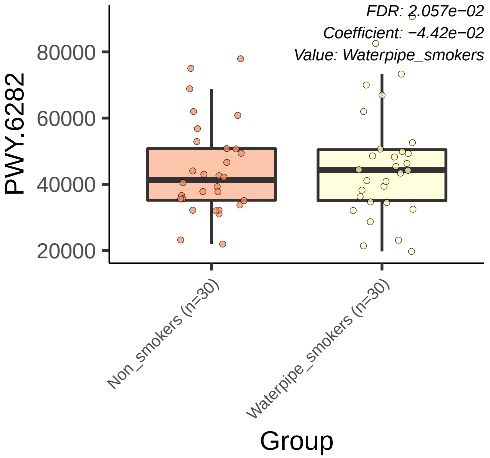
FDR: 2.057e−02
Coefficient: −4.42e−02
Value: Waterpipe_smokers
PWY.6282
80000
60000
40000
20000
Non_smokers (n=30)
Waterpipe_smokers (n=30)
Group

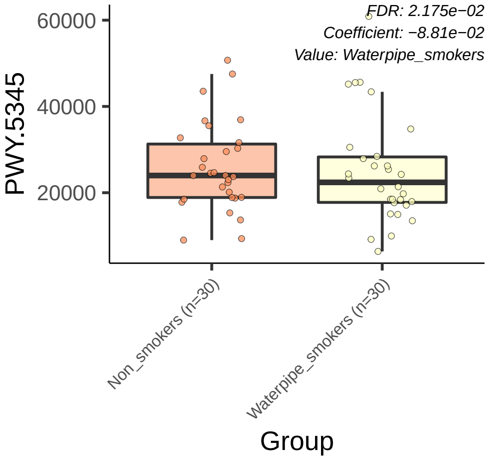

FDR: 2.175e−02
Coefficient: −8.81e−02
Value: Waterpipe_smokers
PWY.5345
60000
40000
20000
Non_smokers (n=30)
Waterpipe_smokers (n=30)
Group

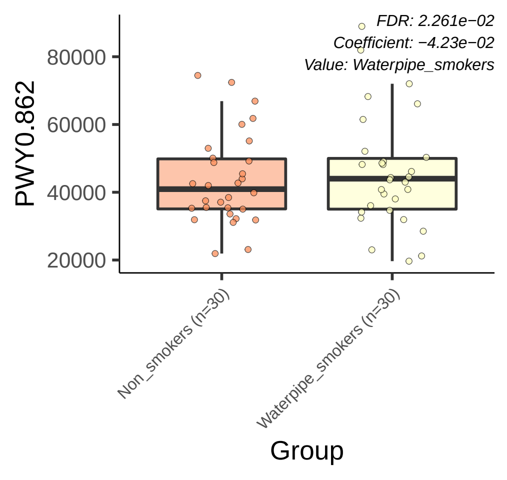
FDR: 2.261e−02
Coefficient: −4.23e−02
Value: Waterpipe_smokers
PWY0.862
80000
60000
40000
20000
Non_smokers (n=30)
Waterpipe_smokers (n=30)
Group

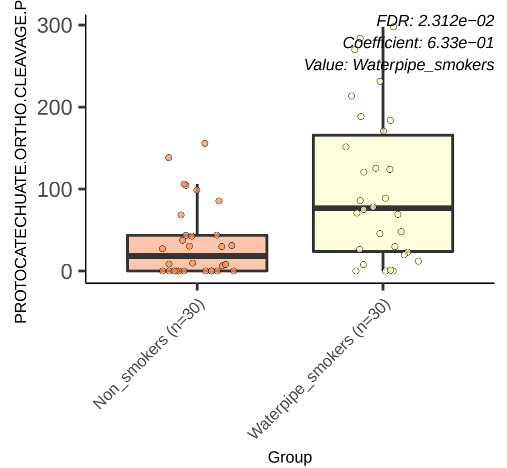

FDR: 2.312e−02
Coefficient: 6.33e−01
Value: Waterpipe_smokers
PROTOCATECHUATE.ORTHO.CLEAVAGE.P
0
100
200
300
Non_smokers (n=30)
Waterpipe_smokers (n=30)
Group

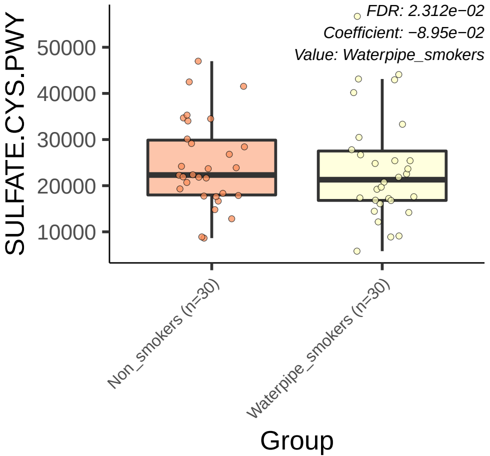
FDR: 2.312e−02
Coefficient: −8.95e−02
Value: Waterpipe_smokers
SULFATE.CYS.PWY
50000
40000
30000
20000
10000
Non_smokers (n=30)
Waterpipe_smokers (n=30)
Group

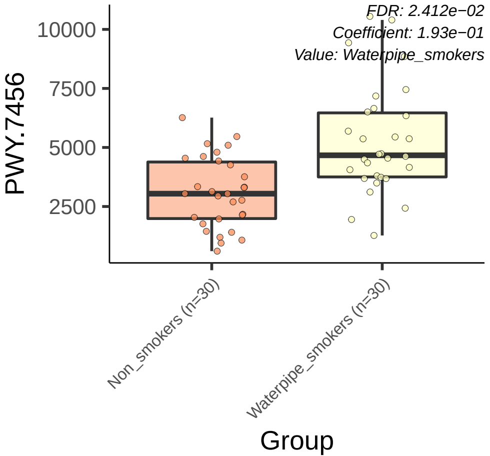

FDR: 2.412e−02
Coefficient: 1.93e−01
Value: Waterpipe_smokers
PWY.7456
10000
7500
5000
2500
Non_smokers (n=30)
Waterpipe_smokers (n=30)
Group

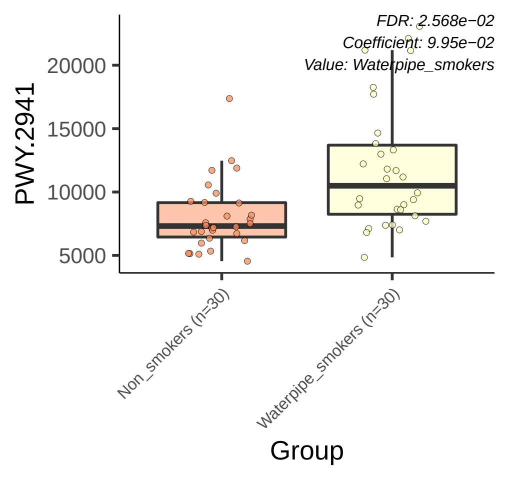

FDR: 2.568e−02
Coefficient: 9.95e−02
Value: Waterpipe_smokers
PWY.2941
20000
15000
10000
5000
Non_smokers (n=30)
Waterpipe_smokers (n=30)
Group

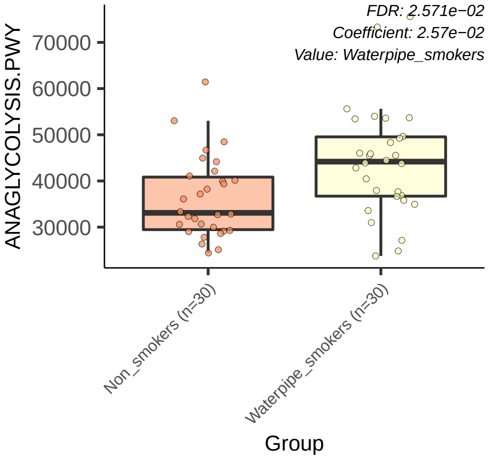
FDR: 2.571e−02
Coefficient: 2.57e−02
Value: Waterpipe_smokers
ANAGLYCOLYSIS.PWY
70000
60000
50000
40000
30000
Non_smokers (n=30)
Waterpipe_smokers (n=30)
Group

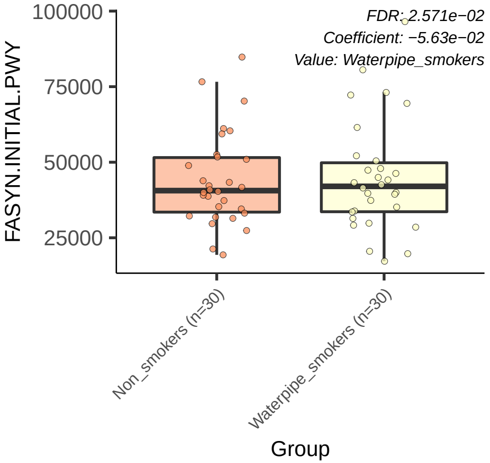
FDR: 2.571e−02
Coefficient: −5.63e−02
Value: Waterpipe_smokers
FASYN.INITIAL.PWY
100000
75000
50000
25000
Non_smokers (n=30)
Waterpipe_smokers (n=30)
Group

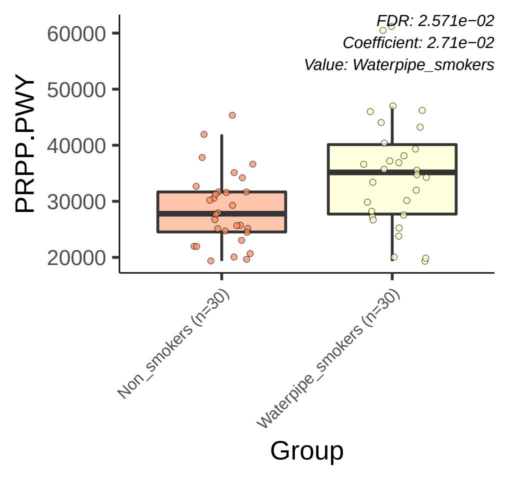
FDR: 2.571e−02
Coefficient: 2.71e−02
Value: Waterpipe_smokers
PRPP.PWY
60000
50000
40000
30000
20000
Non_smokers (n=30)
Waterpipe_smokers (n=30)
Group

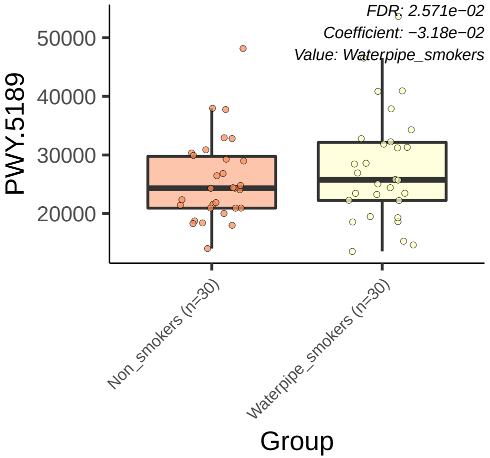
FDR: 2.571e−02
Coefficient: −3.18e−02
Value: Waterpipe_smokers
PWY.5189
50000
40000
30000
20000
Non_smokers (n=30)
Waterpipe_smokers (n=30)
Group

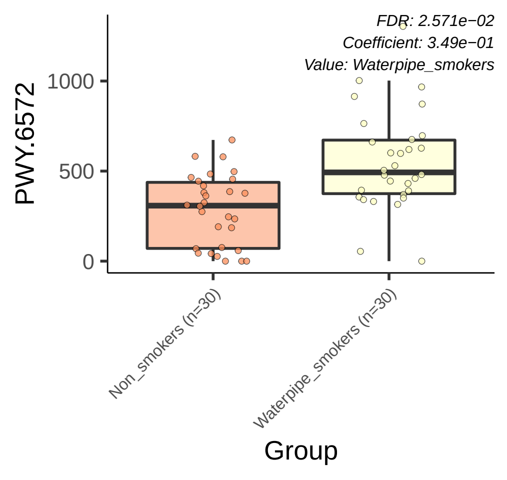
FDR: 2.571e−02
Coefficient: 3.49e−01
Value: Waterpipe_smokers
PWY.6572
1000
500
0
Non_smokers (n=30)
Waterpipe_smokers (n=30)
Group

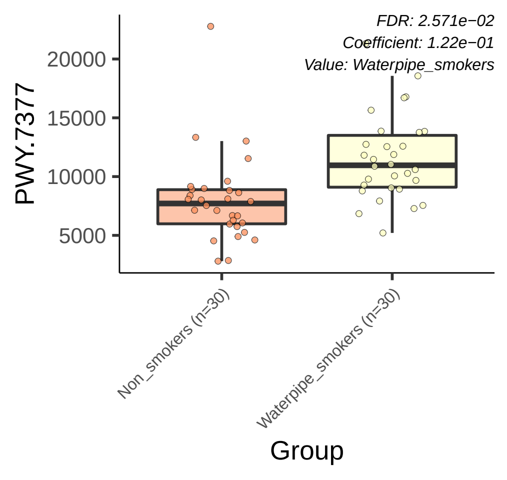

FDR: 2.571e−02
Coefficient: 1.22e−01
Value: Waterpipe_smokers
PWY.7377
20000
15000
10000
5000
Non_smokers (n=30)
Waterpipe_smokers (n=30)
Group

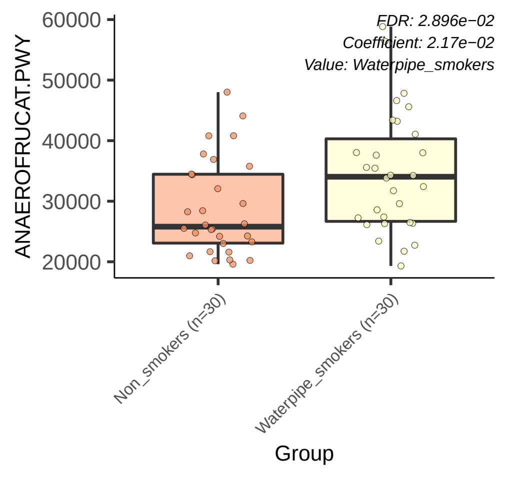
FDR: 2.896e−02
Coefficient: 2.17e−02
Value: Waterpipe_smokers
ANAEROFRUCAT.PWY
60000
50000
40000
30000
20000
Non_smokers (n=30)
Waterpipe_smokers (n=30)
Group

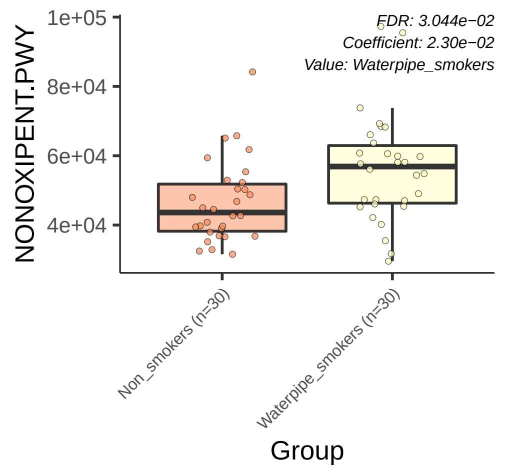

FDR: 3.044e−02
Coefficient: 2.30e−02
Value: Waterpipe_smokers
NONOXIPENT.PWY
1e+05
8e+04
6e+04
4e+04
Non_smokers (n=30)
Waterpipe_smokers (n=30)
Group

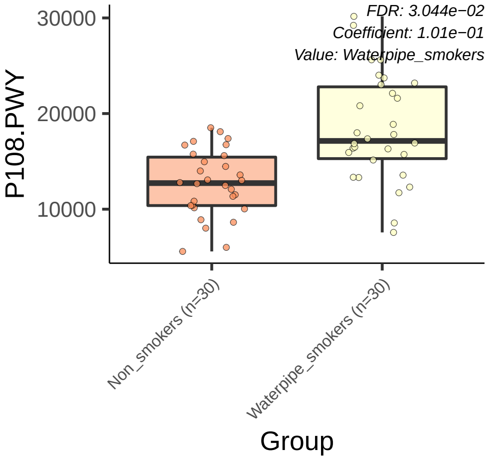
FDR: 3.044e−02
Coefficient: 1.01e−01
Value: Waterpipe_smokers
P108.PWY
30000
20000
10000
Non_smokers (n=30)
Waterpipe_smokers (n=30)
Group

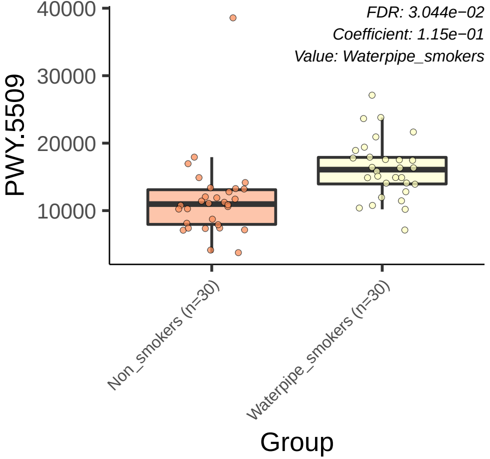
FDR: 3.044e−02
Coefficient: 1.15e−01
Value: Waterpipe_smokers
PWY.5509
40000
30000
20000
10000
Non_smokers (n=30)
Waterpipe_smokers (n=30)
Group

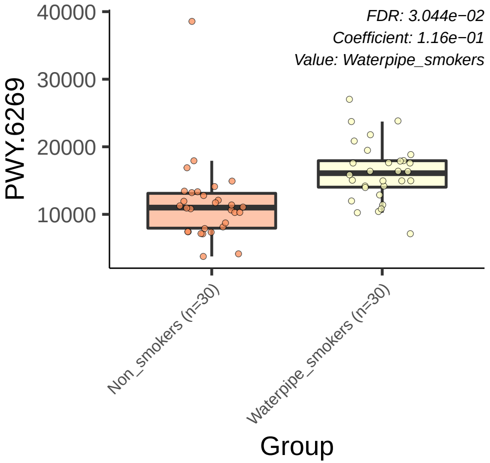
FDR: 3.044e−02
Coefficient: 1.16e−01
Value: Waterpipe_smokers
PWY.6269
40000
30000
20000
10000
Non_smokers (n=30)
Waterpipe_smokers (n=30)
Group

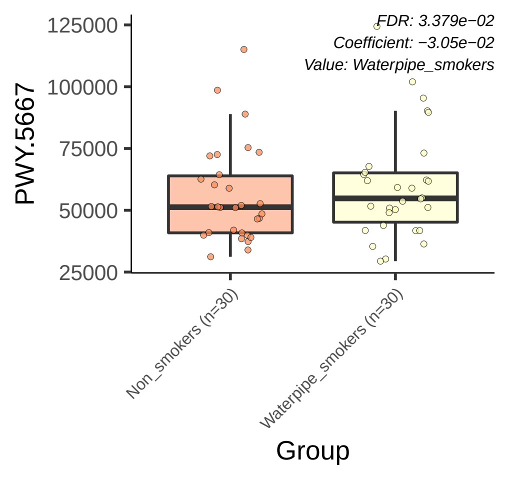

FDR: 3.379e−02
Coefficient: −3.05e−02
Value: Waterpipe_smokers
PWY.5667
125000
100000
75000
50000
25000
Non_smokers (n=30)
Waterpipe_smokers (n=30)
Group

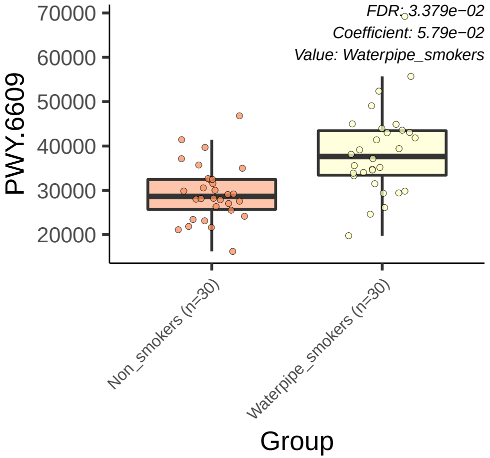

FDR: 3.379e−02
Coefficient: 5.79e−02
Value: Waterpipe_smokers
PWY.6609
70000
60000
50000
40000
30000
20000
Non_smokers (n=30)
Waterpipe_smokers (n=30)
Group

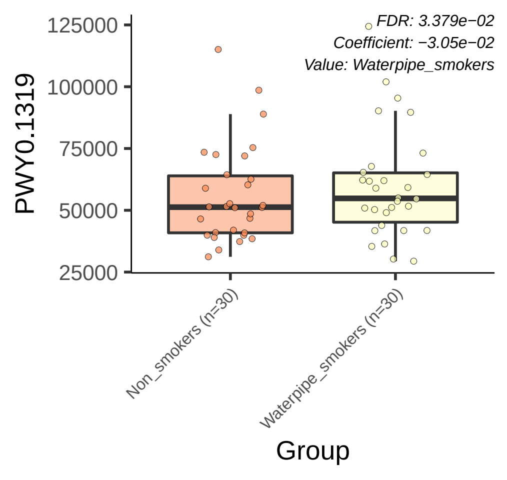

FDR: 3.379e−02
Coefficient: −3.05e−02
Value: Waterpipe_smokers
PWY0.1319
125000
100000
75000
50000
25000
Non_smokers (n=30)
Waterpipe_smokers (n=30)
Group

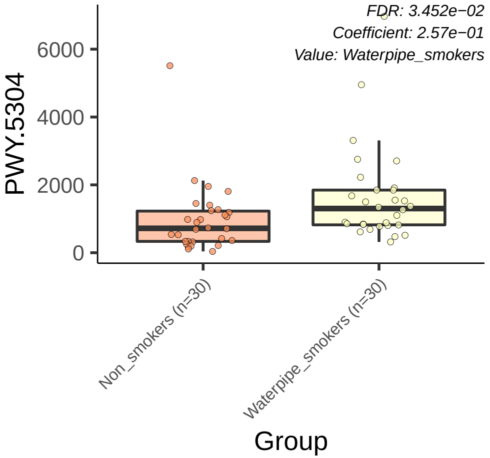

FDR: 3.452e−02
Coefficient: 2.57e−01
Value: Waterpipe_smokers
PWY.5304
0
2000
4000
6000
Non_smokers (n=30)
Waterpipe_smokers (n=30)
Group

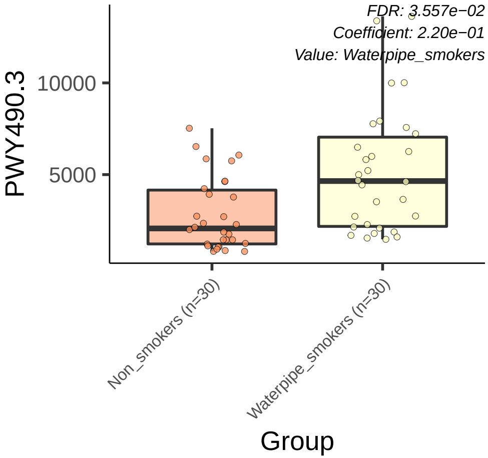

FDR: 3.557e−02
Coefficient: 2.20e−01
Value: Waterpipe_smokers
PWY490.3
10000
5000
Non_smokers (n=30)
Waterpipe_smokers (n=30)
Group

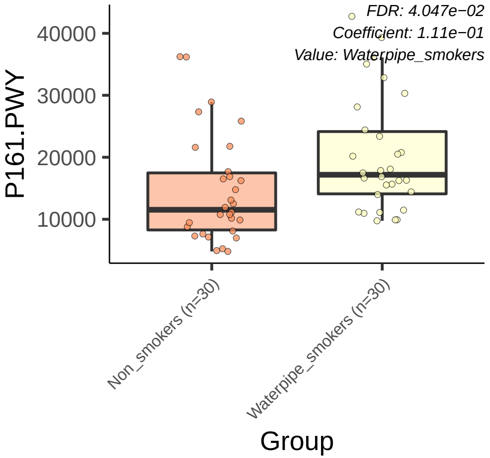
FDR: 4.047e−02
Coefficient: 1.11e−01
Value: Waterpipe_smokers
P161.PWY
40000
30000
20000
10000
Non_smokers (n=30)
Waterpipe_smokers (n=30)
Group

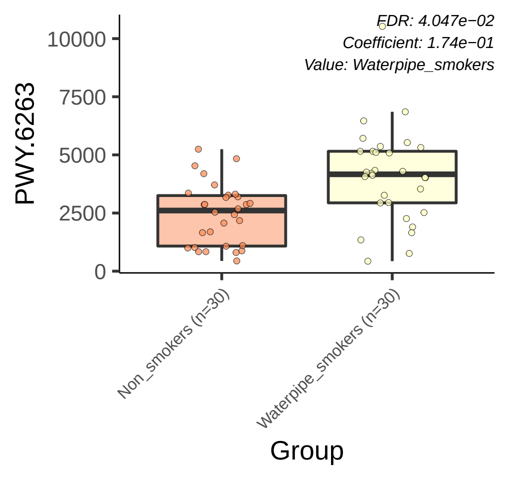
FDR: 4.047e−02
Coefficient: 1.74e−01
Value: Waterpipe_smokers
PWY.6263
10000
7500
5000
2500
0
Non_smokers (n=30)
Waterpipe_smokers (n=30)
Group

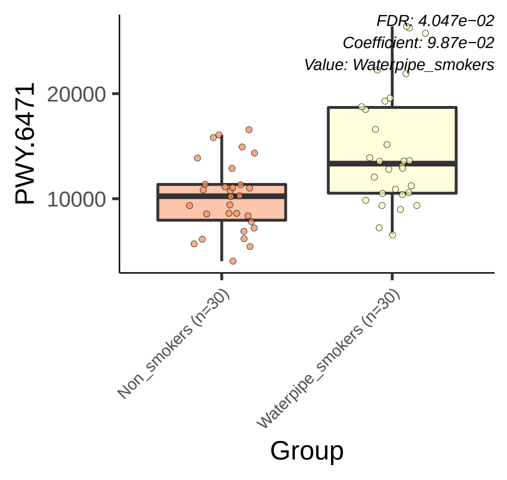

FDR: 4.047e−02
Coefficient: 9.87e−02
Value: Waterpipe_smokers
PWY.6471
20000
10000
Non_smokers (n=30)
Waterpipe_smokers (n=30)
Group

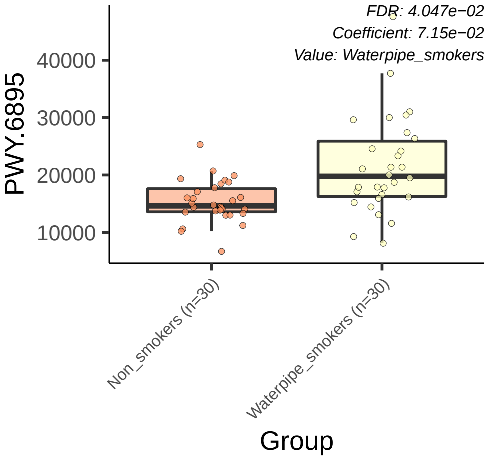

FDR: 4.047e−02
Coefficient: 7.15e−02
Value: Waterpipe_smokers
PWY.6895
40000
30000
20000
10000
Non_smokers (n=30)
Waterpipe_smokers (n=30)
Group

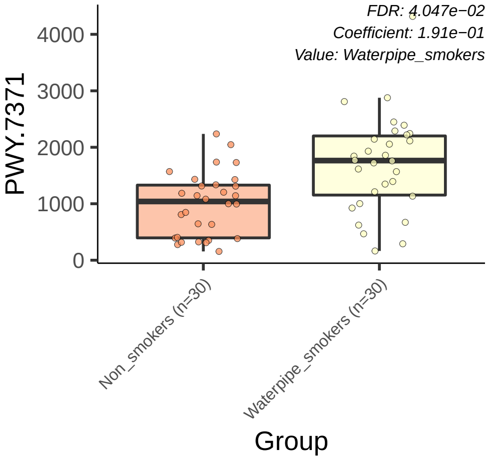

FDR: 4.047e−02
Coefficient: 1.91e−01
Value: Waterpipe_smokers
PWY.7371
4000
3000
2000
1000
0
Non_smokers (n=30)
Waterpipe_smokers (n=30)
Group

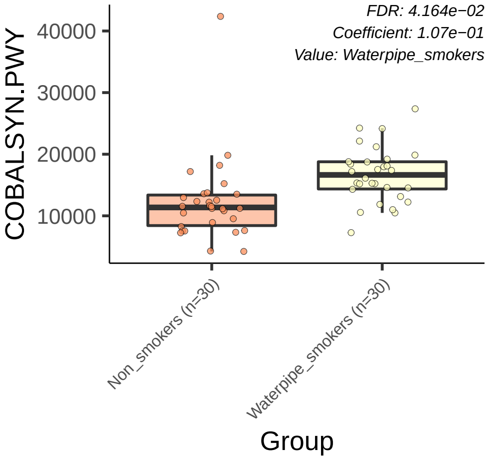

FDR: 4.164e−02
Coefficient: 1.07e−01
Value: Waterpipe_smokers
COBALSYN.PWY
40000
30000
20000
10000
Non_smokers (n=30)
Waterpipe_smokers (n=30)
Group

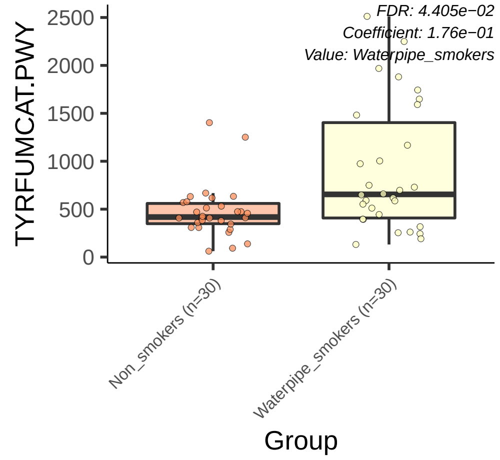

FDR: 4.405e−02
Coefficient: 1.76e−01
Value: Waterpipe_smokers
TYRFUMCAT.PWY
2500
2000
1500
1000
500
0
Non_smokers (n=30)
Waterpipe_smokers (n=30)
Group

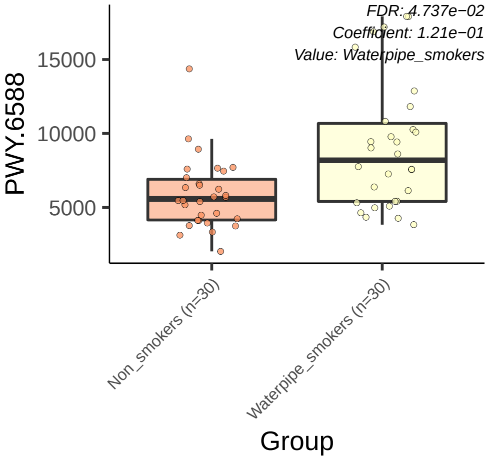

FDR: 4.737e−02
Coefficient: 1.21e−01
Value: Waterpipe_smokers
PWY.6588
15000
10000
5000
Non_smokers (n=30)
Waterpipe_smokers (n=30)
Group

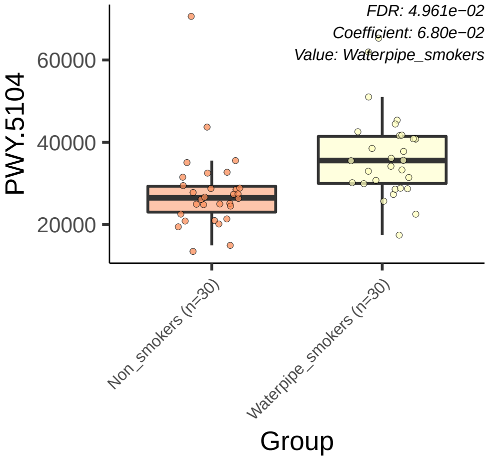

FDR: 4.961e−02
Coefficient: 6.80e−02
Value: Waterpipe_smokers
PWY.5104
60000
40000
20000
Non_smokers (n=30)
Waterpipe_smokers (n=30)
Group

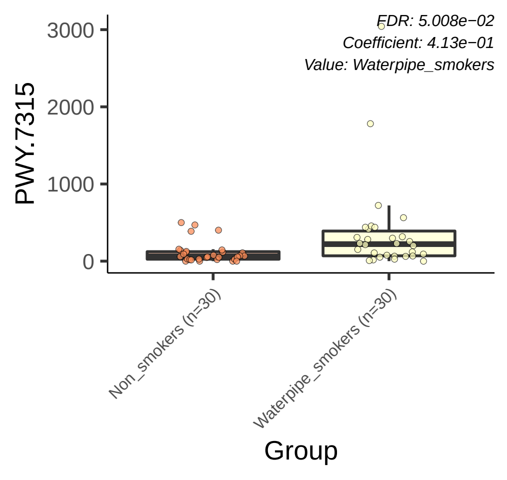

FDR: 5.008e−02
Coefficient: 4.13e−01
Value: Waterpipe_smokers
PWY.7315
3000
2000
1000
0
Non_smokers (n=30)
Waterpipe_smokers (n=30)
Group

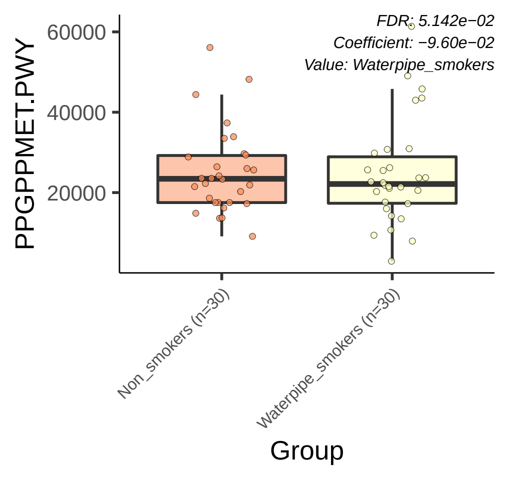

FDR: 5.142e−02
Coefficient: −9.60e−02
Value: Waterpipe_smokers
PPGPPMET.PWY
60000
40000
20000
Non_smokers (n=30)
Waterpipe_smokers (n=30)
Group

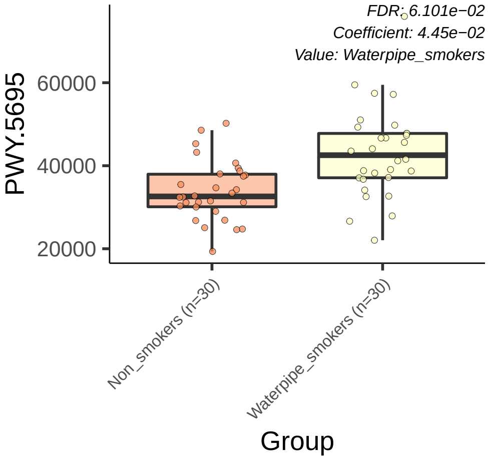
FDR: 6.101e−02
Coefficient: 4.45e−02
Value: Waterpipe_smokers
PWY.5695
60000
40000
20000
Non_smokers (n=30)
Waterpipe_smokers (n=30)
Group

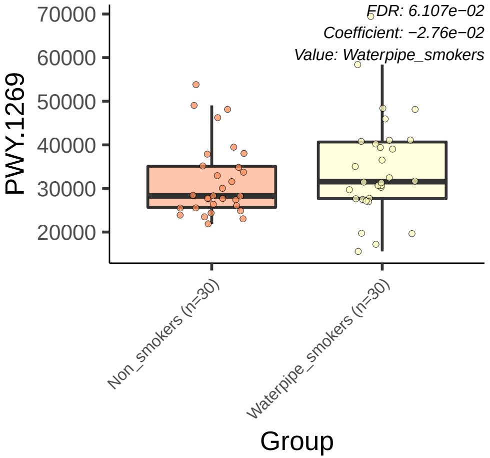

FDR: 6.107e−02
Coefficient: −2.76e−02
Value: Waterpipe_smokers
PWY.1269
70000
60000
50000
40000
30000
20000
Non_smokers (n=30)
Waterpipe_smokers (n=30)
Group

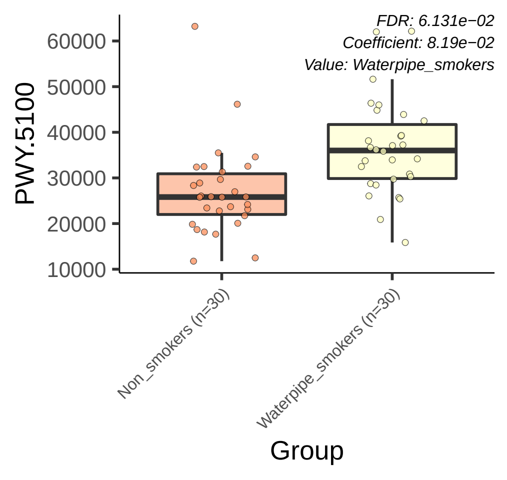
FDR: 6.131e−02
Coefficient: 8.19e−02
Value: Waterpipe_smokers
PWY.5100
60000
50000
40000
30000
20000
10000
Non_smokers (n=30)
Waterpipe_smokers (n=30)
Group

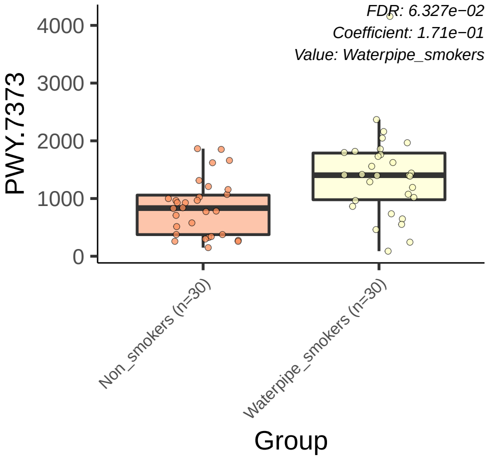

FDR: 6.327e−02
Coefficient: 1.71e−01
Value: Waterpipe_smokers
PWY.7373
4000
3000
2000
1000
0
Non_smokers (n=30)
Waterpipe_smokers (n=30)
Group

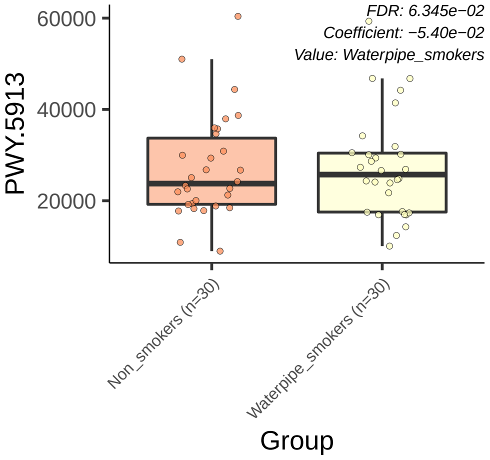

FDR: 6.345e−02
Coefficient: −5.40e−02
Value: Waterpipe_smokers
PWY.5913
60000
40000
20000
Non_smokers (n=30)
Waterpipe_smokers (n=30)
Group

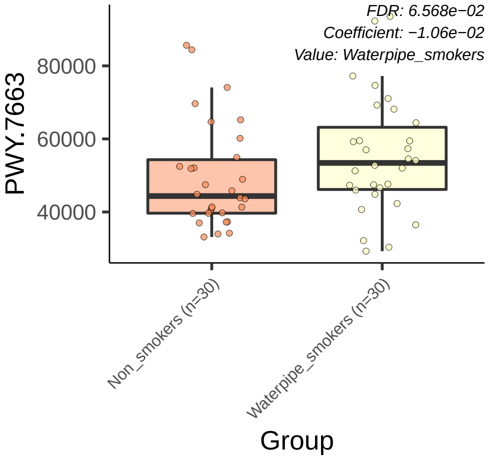

FDR: 6.568e−02
Coefficient: −1.06e−02
Value: Waterpipe_smokers
PWY.7663
80000
60000
40000
Non_smokers (n=30)
Waterpipe_smokers (n=30)
Group

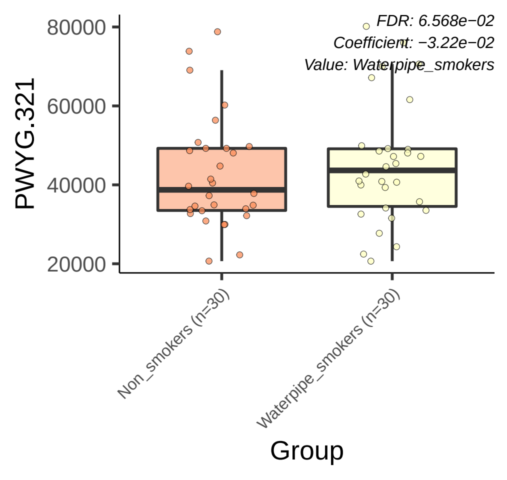

FDR: 6.568e−02
Coefficient: −3.22e−02
Value: Waterpipe_smokers
PWYG.321
80000
60000
40000
20000
Non_smokers (n=30)
Waterpipe_smokers (n=30)
Group

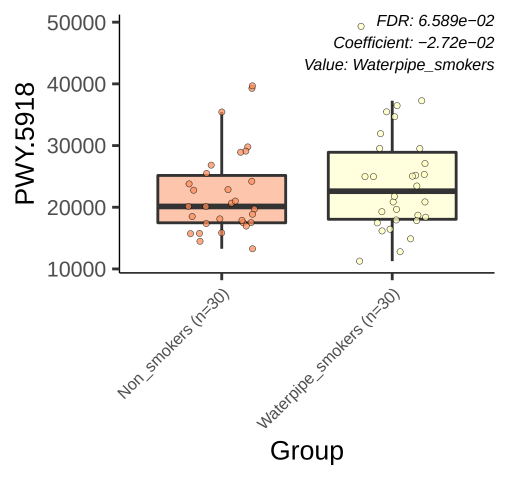

FDR: 6.589e−02
Coefficient: −2.72e−02
Value: Waterpipe_smokers
PWY.5918
50000
40000
30000
20000
10000
Non_smokers (n=30)
Waterpipe_smokers (n=30)
Group

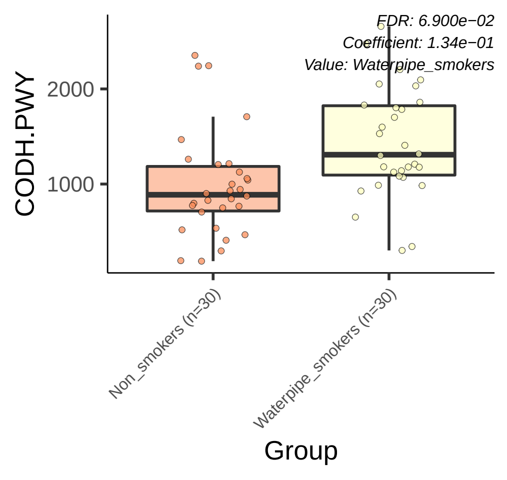

FDR: 6.900e−02
Coefficient: 1.34e−01
Value: Waterpipe_smokers
CODH.PWY
2000
1000
Non_smokers (n=30)
Waterpipe_smokers (n=30)
Group

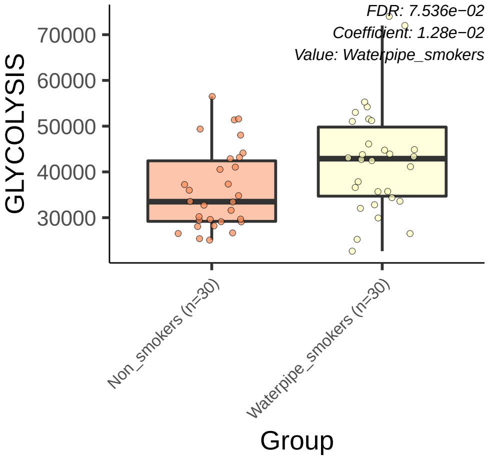
FDR: 7.536e−02
Coefficient: 1.28e−02
Value: Waterpipe_smokers
GLYCOLYSIS
70000
60000
50000
40000
30000
Non_smokers (n=30)
Waterpipe_smokers (n=30)
Group

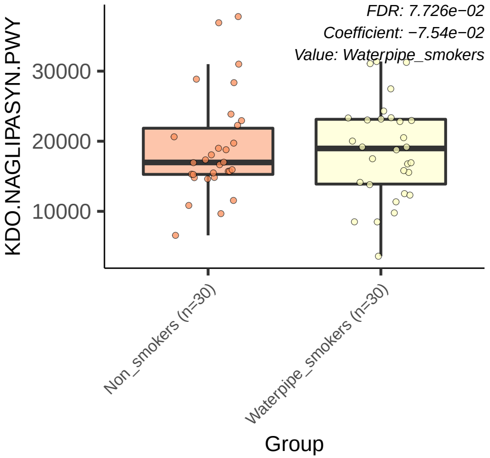

FDR: 7.726e−02
Coefficient: −7.54e−02
Value: Waterpipe_smokers
KDO.NAGLIPASYN.PWY
30000
20000
10000
Non_smokers (n=30)
Waterpipe_smokers (n=30)
Group

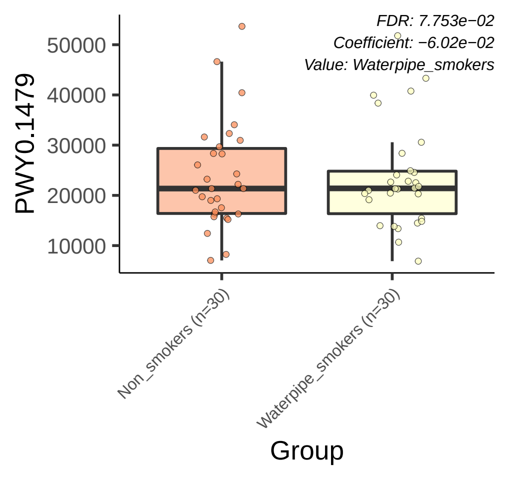
FDR: 7.753e−02
Coefficient: −6.02e−02
Value: Waterpipe_smokers
PWY0.1479
50000
40000
30000
20000
10000
Non_smokers (n=30)
Waterpipe_smokers (n=30)
Group

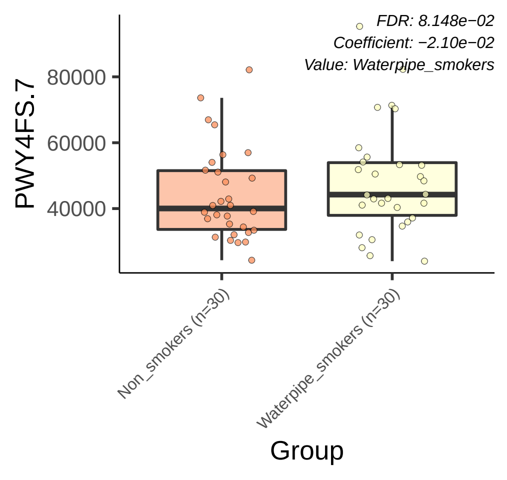
FDR: 8.148e−02
Coefficient: −2.10e−02
Value: Waterpipe_smokers
PWY4FS.7
80000
60000
40000
Non_smokers (n=30)
Waterpipe_smokers (n=30)
Group

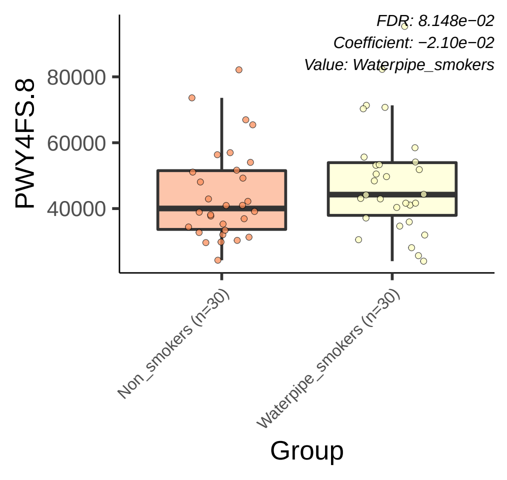

FDR: 8.148e−02
Coefficient: −2.10e−02
Value: Waterpipe_smokers
PWY4FS.8
80000
60000
40000
Non_smokers (n=30)
Waterpipe_smokers (n=30)
Group

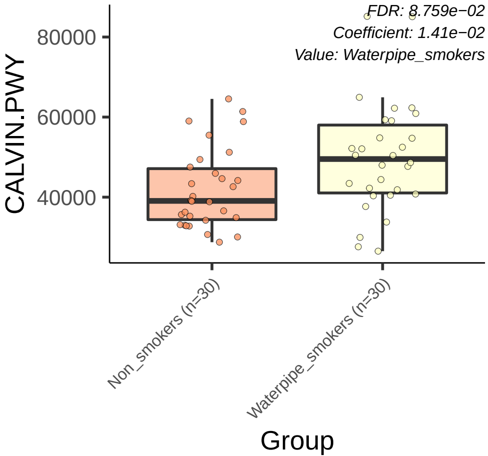

FDR: 8.759e−02
Coefficient: 1.41e−02
Value: Waterpipe_smokers
CALVIN.PWY
80000
60000
40000
Non_smokers (n=30)
Waterpipe_smokers (n=30)
Group

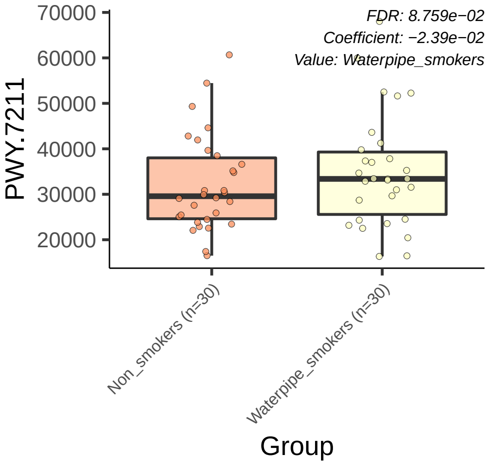

FDR: 8.759e−02
Coefficient: −2.39e−02
Value: Waterpipe_smokers
PWY.7211
70000
60000
50000
40000
30000
20000
Non_smokers (n=30)
Waterpipe_smokers (n=30)
Group

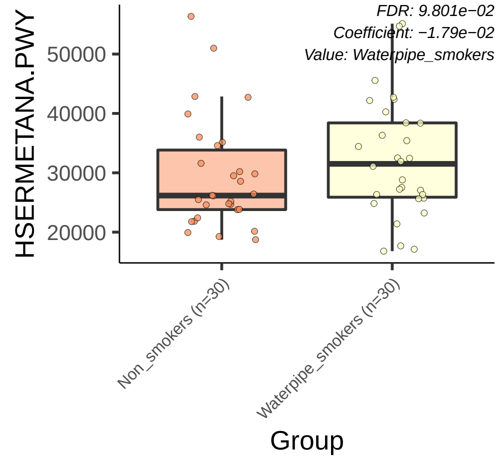

FDR: 9.801e−02
Coefficient: −1.79e−02
Value: Waterpipe_smokers
HSERMETANA.PWY
50000
40000
30000
20000
Non_smokers (n=30)
Waterpipe_smokers (n=30)
Group

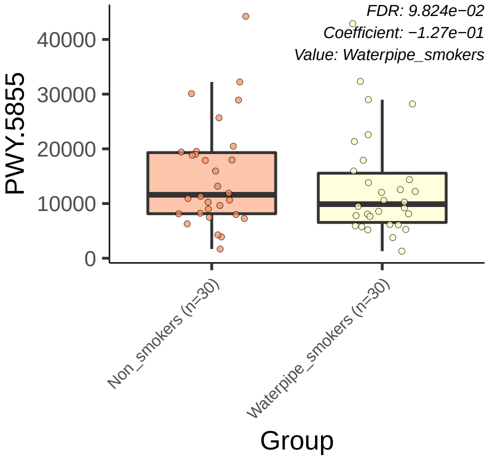
FDR: 9.824e−02
Coefficient: −1.27e−01
Value: Waterpipe_smokers
PWY.5855
40000
30000
20000
10000
0
Non_smokers (n=30)
Waterpipe_smokers (n=30)
Group

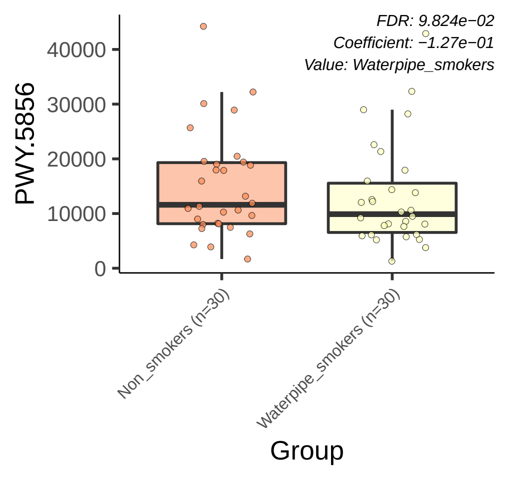

FDR: 9.824e−02
Coefficient: −1.27e−01
Value: Waterpipe_smokers
PWY.5856
40000
30000
20000
10000
0
Non_smokers (n=30)
Waterpipe_smokers (n=30)
Group

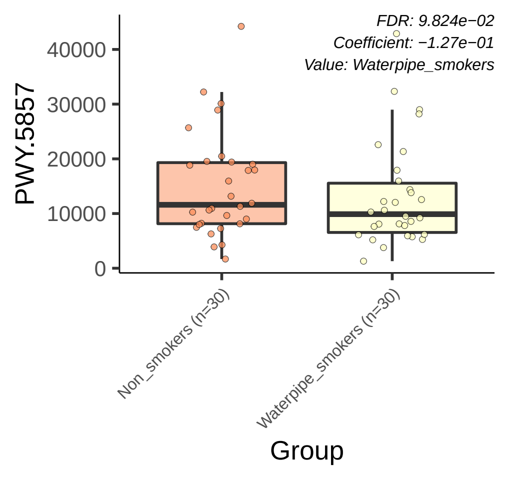

FDR: 9.824e−02
Coefficient: −1.27e−01
Value: Waterpipe_smokers
PWY.5857
40000
30000
20000
10000
0
Non_smokers (n=30)
Waterpipe_smokers (n=30)
Group

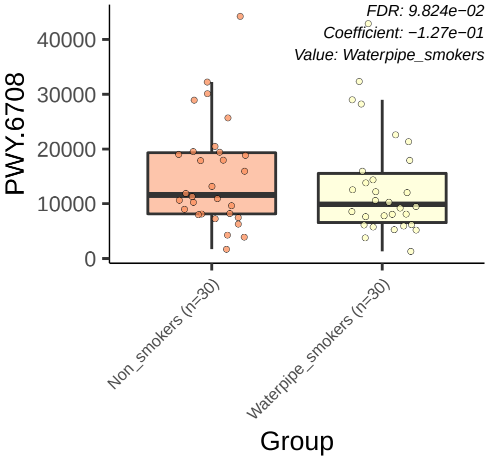
FDR: 9.824e−02
Coefficient: −1.27e−01
Value: Waterpipe_smokers
PWY.6708
40000
30000
20000
10000
0
Non_smokers (n=30)
Waterpipe_smokers (n=30)
Group

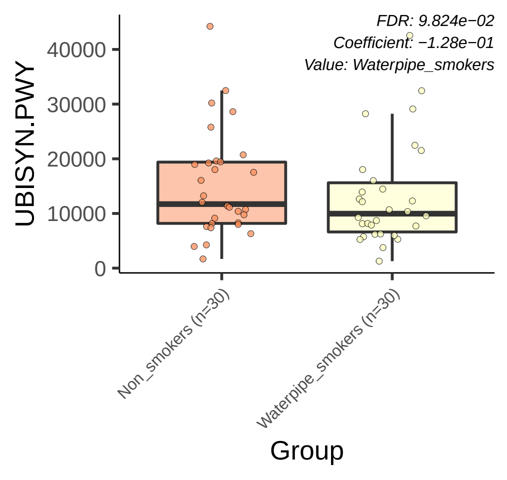
FDR: 9.824e−02
Coefficient: −1.28e−01
Value: Waterpipe_smokers
UBISYN.PWY
40000
30000
20000
10000
0
Non_smokers (n=30)
Waterpipe_smokers (n=30)
Group

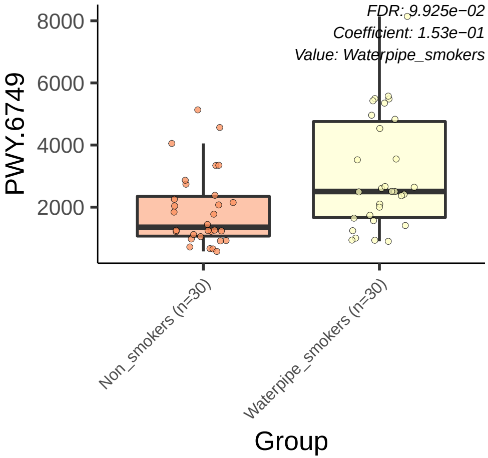
FDR: 9.925e−02
Coefficient: 1.53e−01
Value: Waterpipe_smokers
8000
6000
4000
2000
PWY.6749
Non_smokers (n=30)
Waterpipe_smokers (n=30)
Group

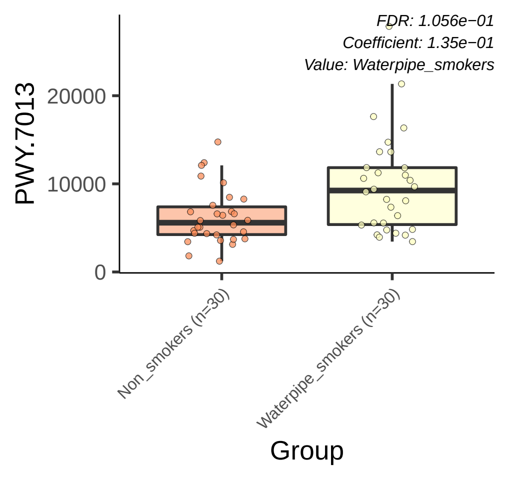

FDR: 1.056e−01
Coefficient: 1.35e−01
Value: Waterpipe_smokers
PWY.7013
20000
10000
0
Non_smokers (n=30)
Waterpipe_smokers (n=30)
Group

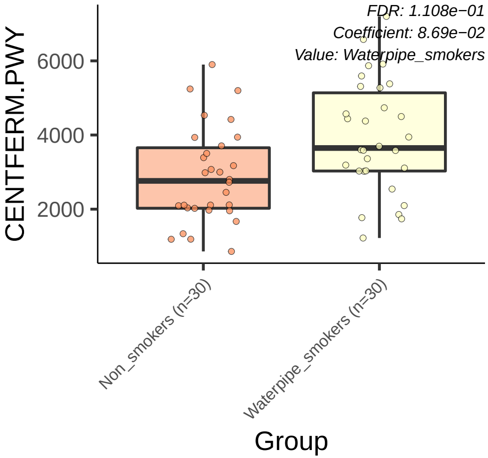
FDR: 1.108e−01
Coefficient: 8.69e−02
Value: Waterpipe_smokers
CENTFERM.PWY
6000
4000
2000
Non_smokers (n=30)
Waterpipe_smokers (n=30)
Group

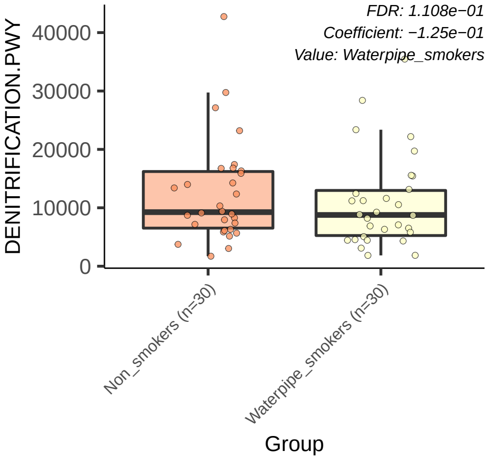

FDR: 1.108e−01
Coefficient: −1.25e−01
Value: Waterpipe_smokers
DENITRIFICATION.PWY
40000
30000
20000
10000
0
Non_smokers (n=30)
Waterpipe_smokers (n=30)
Group

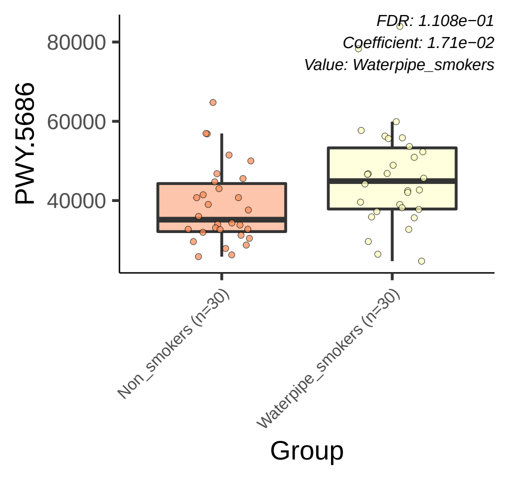

FDR: 1.108e−01
Coefficient: 1.71e−02
Value: Waterpipe_smokers
PWY.5686
80000
60000
40000
Non_smokers (n=30)
Waterpipe_smokers (n=30)
Group

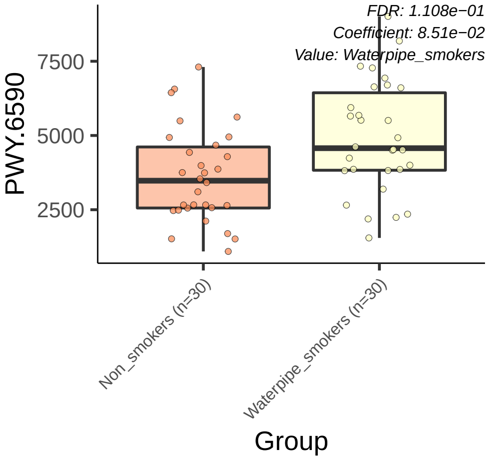
FDR: 1.108e−01
Coefficient: 8.51e−02
Value: Waterpipe_smokers
PWY.6590
7500
5000
2500
Non_smokers (n=30)
Waterpipe_smokers (n=30)
Group

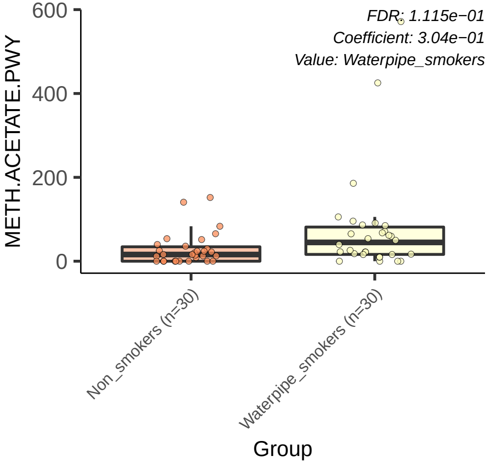

FDR: 1.115e−01
Coefficient: 3.04e−01
Value: Waterpipe_smokers
METH.ACETATE.PWY
600
400
200
0
Non_smokers (n=30)
Waterpipe_smokers (n=30)
Group

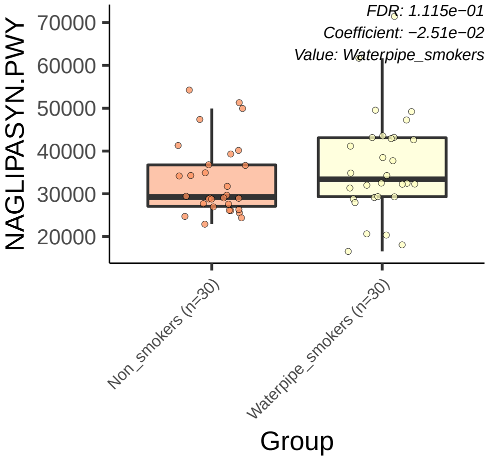
FDR: 1.115e−01
Coefficient: −2.51e−02
Value: Waterpipe_smokers
NAGLIPASYN.PWY
70000
60000
50000
40000
30000
20000
Non_smokers (n=30)
Waterpipe_smokers (n=30)
Group

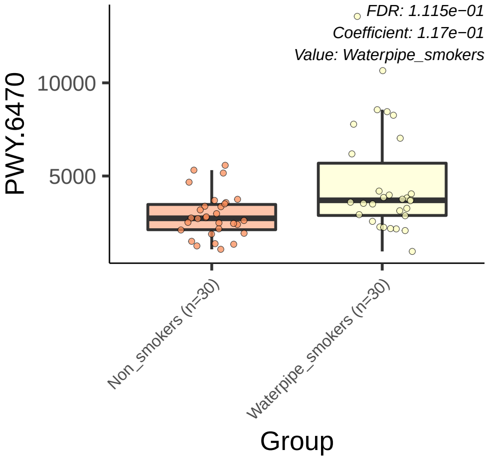
FDR: 1.115e−01
Coefficient: 1.17e−01
Value: Waterpipe_smokers
PWY.6470
10000
5000
Non_smokers (n=30)
Waterpipe_smokers (n=30)
Group

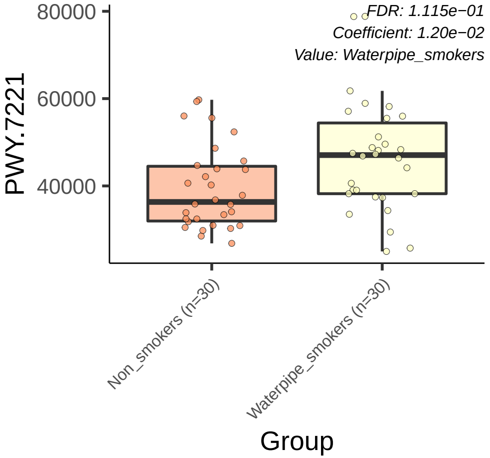
FDR: 1.115e−01
Coefficient: 1.20e−02
Value: Waterpipe_smokers
PWY.7221
80000
60000
40000
Non_smokers (n=30)
Waterpipe_smokers (n=30)
Group

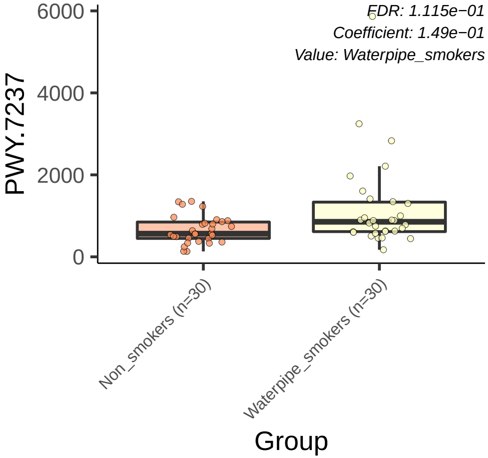

FDR: 1.115e−01
Coefficient: 1.49e−01
Value: Waterpipe_smokers
PWY.7237
6000
4000
2000
0
Non_smokers (n=30)
Waterpipe_smokers (n=30)
Group

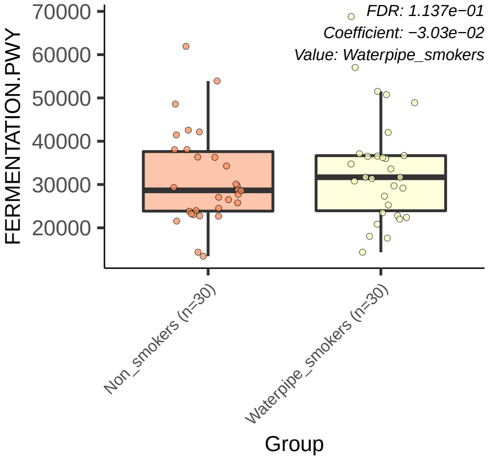
FDR: 1.137e−01
Coefficient: −3.03e−02
Value: Waterpipe_smokers
FERMENTATION.PWY
70000
60000
50000
40000
30000
20000
Non_smokers (n=30)
Waterpipe_smokers (n=30)
Group

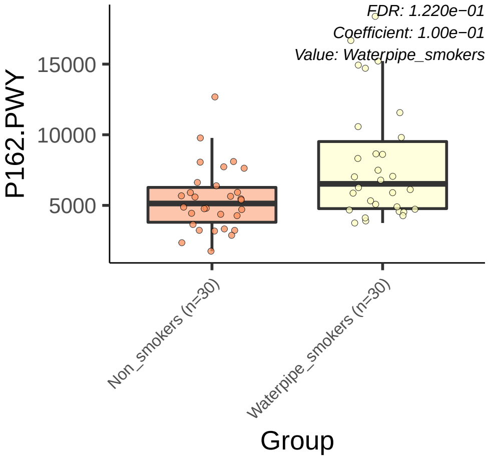
FDR: 1.220e−01
Coefficient: 1.00e−01
Value: Waterpipe_smokers
P162.PWY
15000
10000
5000
Non_smokers (n=30)
Waterpipe_smokers (n=30)
Group

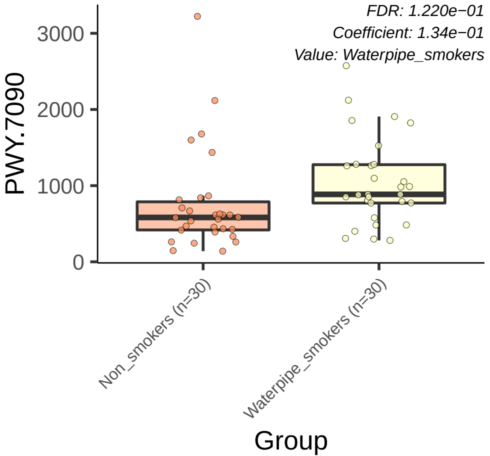

FDR: 1.220e−01
Coefficient: 1.34e−01
Value: Waterpipe_smokers
PWY.7090
3000
2000
1000
0
Non_smokers (n=30)
Waterpipe_smokers (n=30)
Group

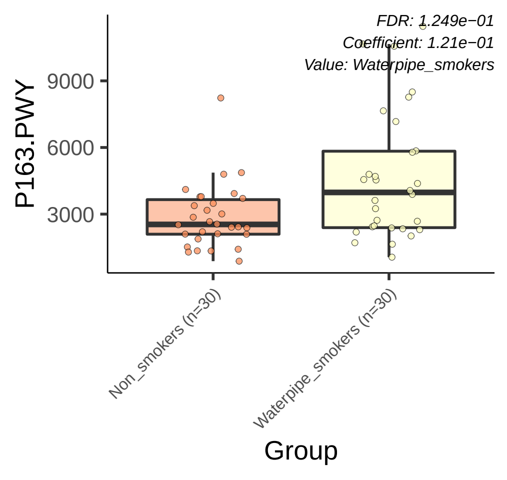
FDR: 1.249e−01
Coefficient: 1.21e−01
Value: Waterpipe_smokers
P163.PWY
9000
6000
3000
Non_smokers (n=30)
Waterpipe_smokers (n=30)
Group

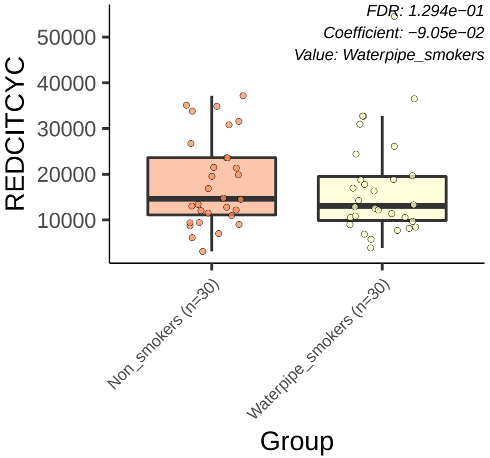
FDR: 1.294e−01
Coefficient: −9.05e−02
Value: Waterpipe_smokers
REDCITCYC
50000
40000
30000
20000
10000
Non_smokers (n=30)
Waterpipe_smokers (n=30)
Group

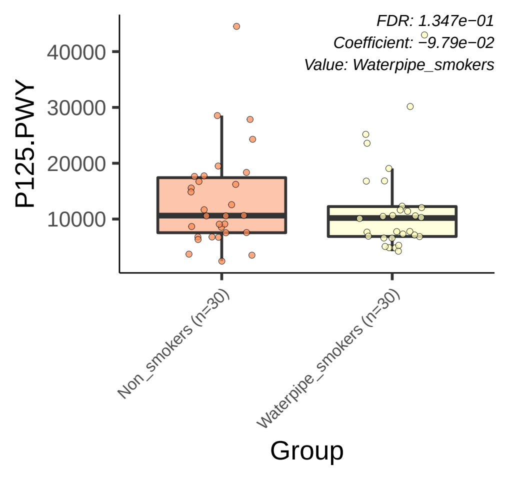

FDR: 1.347e−01
Coefficient: −9.79e−02
Value: Waterpipe_smokers
P125.PWY
40000
30000
20000
10000
Non_smokers (n=30)
Waterpipe_smokers (n=30)
Group

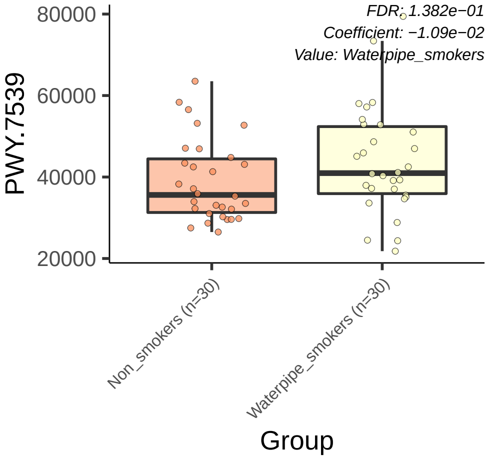

FDR: 1.382e−01
Coefficient: −1.09e−02
Value: Waterpipe_smokers
PWY.7539
80000
60000
40000
20000
Non_smokers (n=30)
Waterpipe_smokers (n=30)
Group

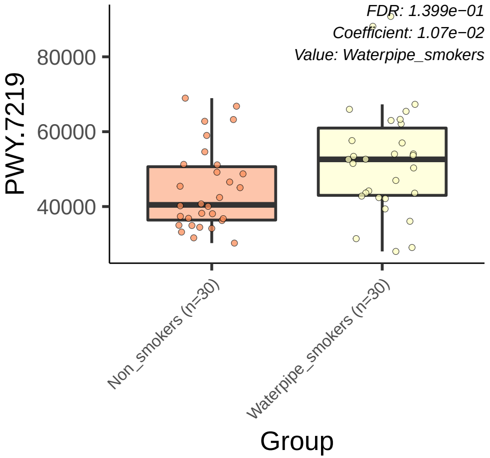
FDR: 1.399e−01
Coefficient: 1.07e−02
Value: Waterpipe_smokers
80000
60000
40000
PWY.7219
Non_smokers (n=30)
Waterpipe_smokers (n=30)
Group

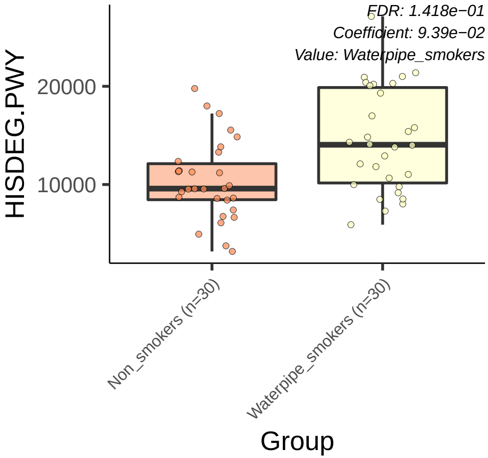

FDR: 1.418e−01
Coefficient: 9.39e−02
Value: Waterpipe_smokers
HISDEG.PWY
20000
10000
Non_smokers (n=30)
Waterpipe_smokers (n=30)
Group

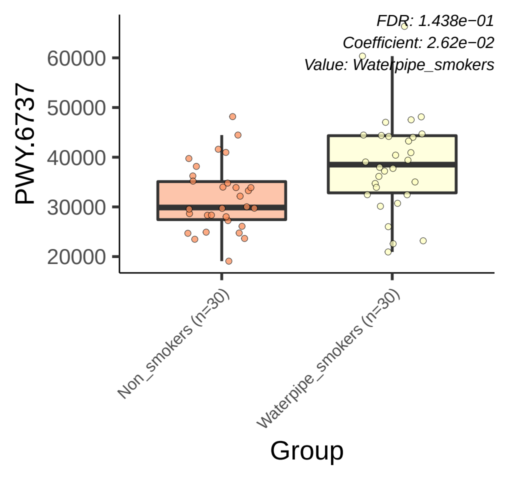
FDR: 1.438e−01
Coefficient: 2.62e−02
Value: Waterpipe_smokers
PWY.6737
60000
50000
40000
30000
20000
Non_smokers (n=30)
Waterpipe_smokers (n=30)
Group

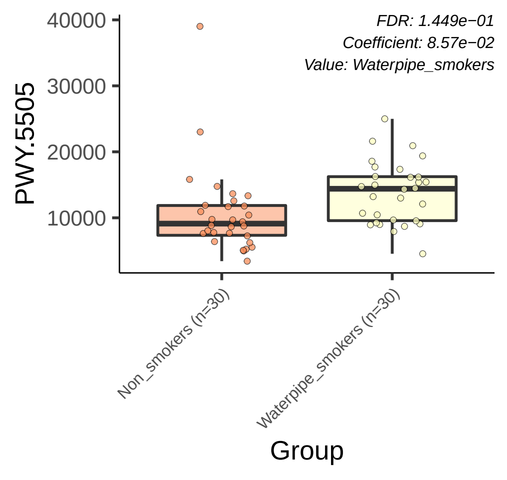

FDR: 1.449e−01
Coefficient: 8.57e−02
Value: Waterpipe_smokers
PWY.5505
40000
30000
20000
10000
Non_smokers (n=30)
Waterpipe_smokers (n=30)
Group

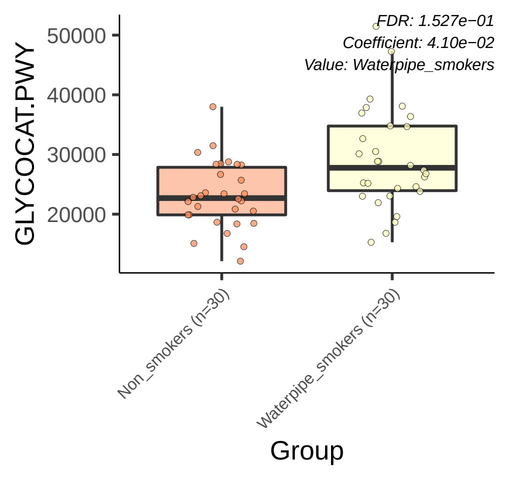
FDR: 1.527e−01
Coefficient: 4.10e−02
Value: Waterpipe_smokers
GLYCOCAT.PWY
50000
40000
30000
20000
Non_smokers (n=30)
Waterpipe_smokers (n=30)
Group

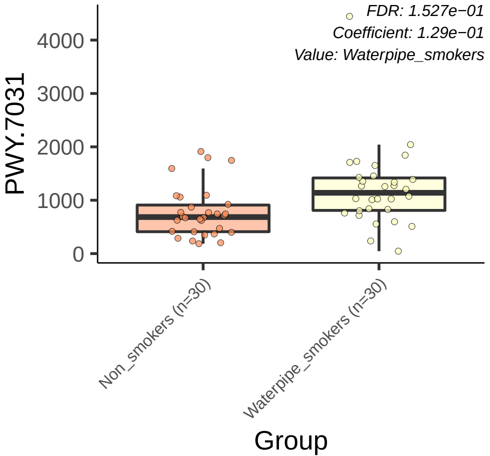

FDR: 1.527e−01
Coefficient: 1.29e−01
Value: Waterpipe_smokers
PWY.7031
4000
3000
2000
1000
0
Non_smokers (n=30)
Waterpipe_smokers (n=30)
Group

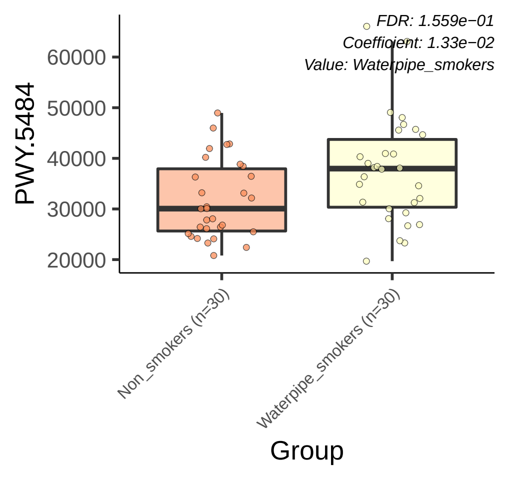
FDR: 1.559e−01
Coefficient: 1.33e−02
Value: Waterpipe_smokers
PWY.5484
60000
50000
40000
30000
20000
Non_smokers (n=30)
Waterpipe_smokers (n=30)
Group

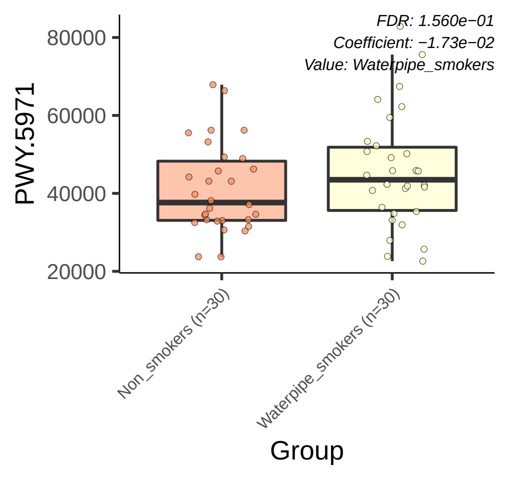
FDR: 1.560e−01
Coefficient: −1.73e−02
Value: Waterpipe_smokers
PWY.5971
80000
60000
40000
20000
Non_smokers (n=30)
Waterpipe_smokers (n=30)
Group

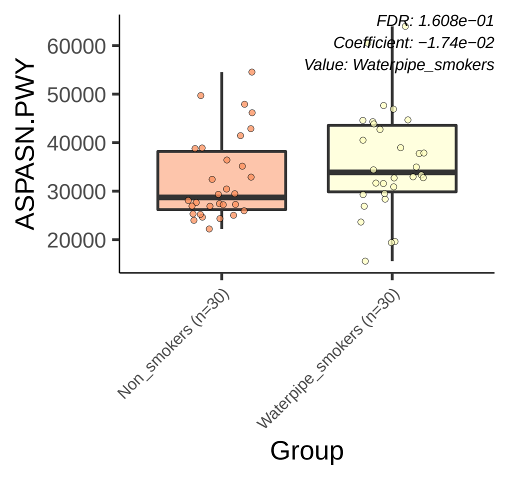

FDR: 1.608e−01
Coefficient: −1.74e−02
Value: Waterpipe_smokers
ASPASN.PWY
60000
50000
40000
30000
20000
Non_smokers (n=30)
Waterpipe_smokers (n=30)
Group

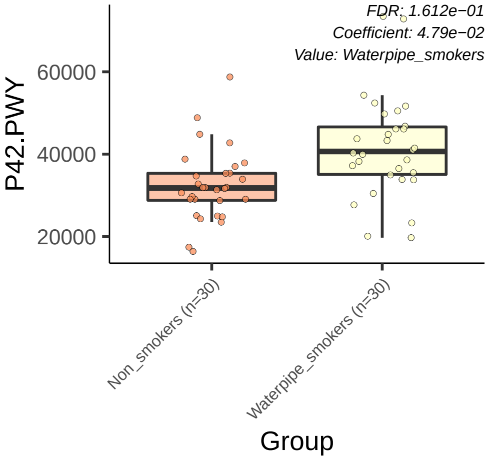

FDR: 1.612e−01
Coefficient: 4.79e−02
Value: Waterpipe_smokers
P42.PWY
60000
40000
20000
Non_smokers (n=30)
Waterpipe_smokers (n=30)
Group

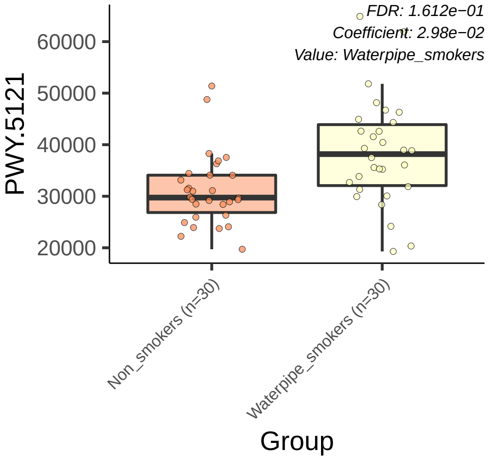

FDR: 1.612e−01
Coefficient: 2.98e−02
Value: Waterpipe_smokers
PWY.5121
60000
50000
40000
30000
20000
Non_smokers (n=30)
Waterpipe_smokers (n=30)
Group

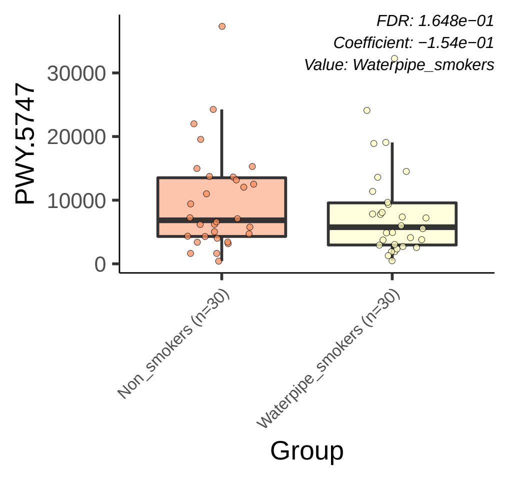
FDR: 1.648e−01
Coefficient: −1.54e−01
Value: Waterpipe_smokers
PWY.5747
30000
20000
10000
0
Non_smokers (n=30)
Waterpipe_smokers (n=30)
Group

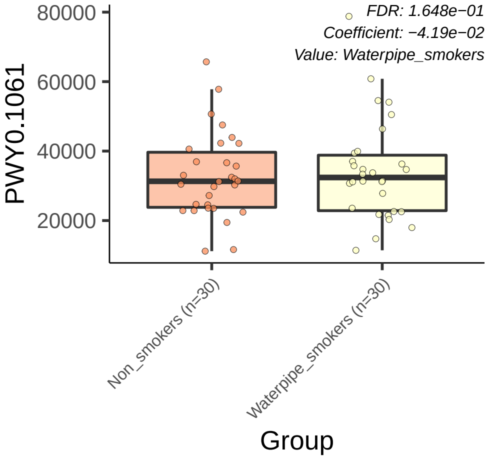

FDR: 1.648e−01
Coefficient: −4.19e−02
Value: Waterpipe_smokers
PWY0.1061
80000
60000
40000
20000
Non_smokers (n=30)
Waterpipe_smokers (n=30)
Group

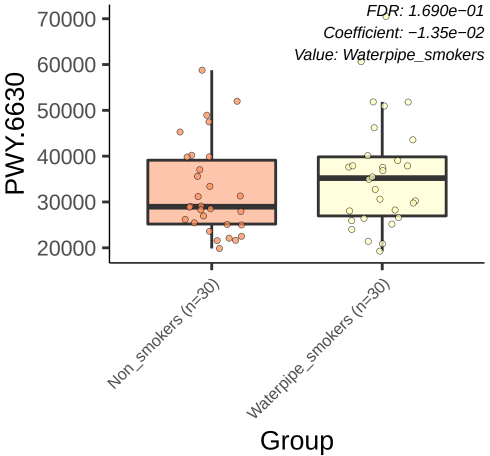
FDR: 1.690e−01
Coefficient: −1.35e−02
Value: Waterpipe_smokers
PWY.6630
70000
60000
50000
40000
30000
20000
Non_smokers (n=30)
Waterpipe_smokers (n=30)
Group

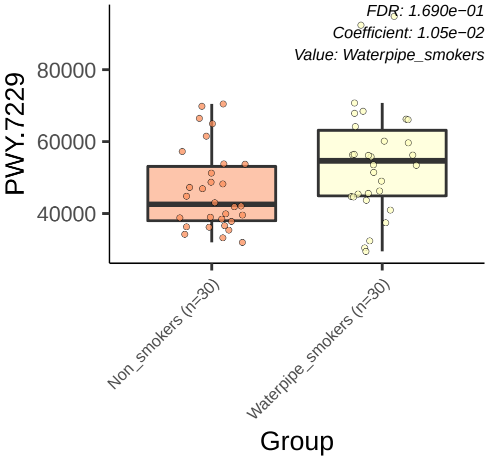
FDR: 1.690e−01
Coefficient: 1.05e−02
Value: Waterpipe_smokers
80000
60000
40000
PWY.7229
Non_smokers (n=30)
Waterpipe_smokers (n=30)
Group

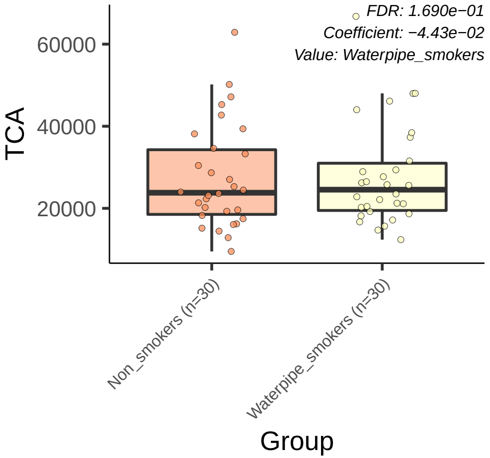
FDR: 1.690e−01
Coefficient: −4.43e−02
Value: Waterpipe_smokers
TCA
60000
40000
20000
Non_smokers (n=30)
Waterpipe_smokers (n=30)
Group

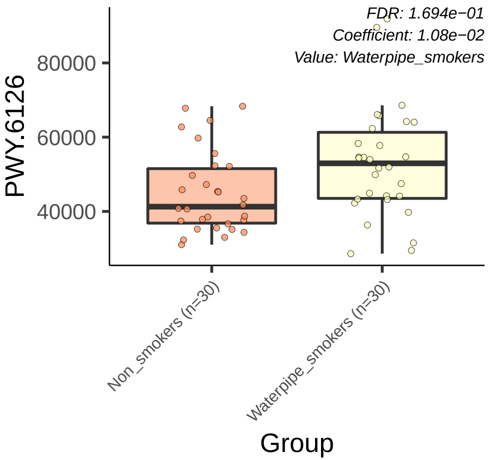
FDR: 1.694e−01
Coefficient: 1.08e−02
Value: Waterpipe_smokers
PWY.6126
80000
60000
40000
Non_smokers (n=30)
Waterpipe_smokers (n=30)
Group

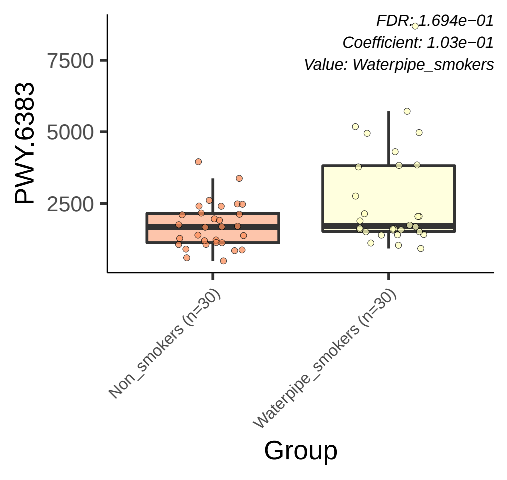

FDR: 1.694e−01
Coefficient: 1.03e−01
Value: Waterpipe_smokers
PWY.6383
7500
5000
2500
Non_smokers (n=30)
Waterpipe_smokers (n=30)
Group

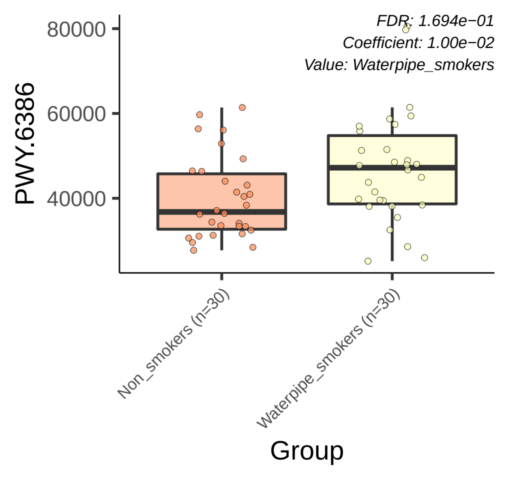

FDR: 1.694e−01
Coefficient: 1.00e−02
Value: Waterpipe_smokers
80000
60000
40000
PWY.6386
Non_smokers (n=30)
Waterpipe_smokers (n=30)
Group

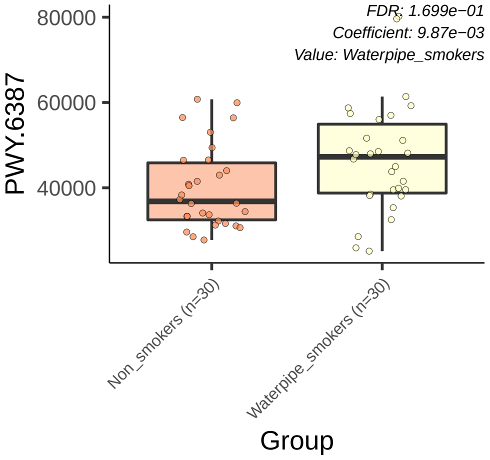

FDR: 1.699e−01
Coefficient: 9.87e−03
Value: Waterpipe_smokers
PWY.6387
80000
60000
40000
Non_smokers (n=30)
Waterpipe_smokers (n=30)
Group

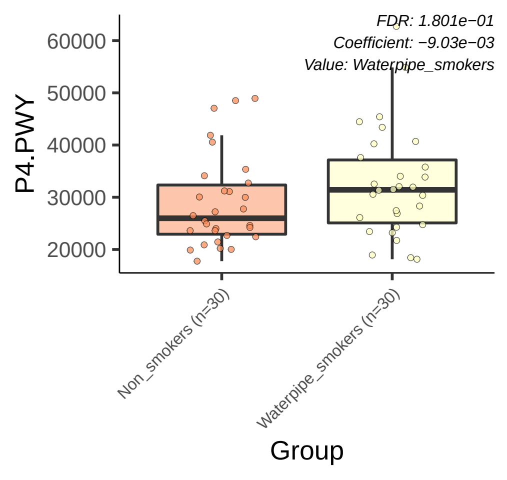

FDR: 1.801e−01
Coefficient: −9.03e−03
Value: Waterpipe_smokers
P4.PWY
60000
50000
40000
30000
20000
Non_smokers (n=30)
Waterpipe_smokers (n=30)
Group

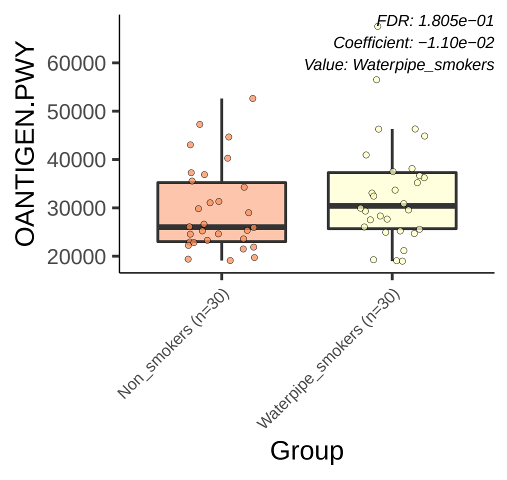
FDR: 1.805e−01
Coefficient: −1.10e−02
Value: Waterpipe_smokers
OANTIGEN.PWY
60000
50000
40000
30000
20000
Non_smokers (n=30)
Waterpipe_smokers (n=30)
Group

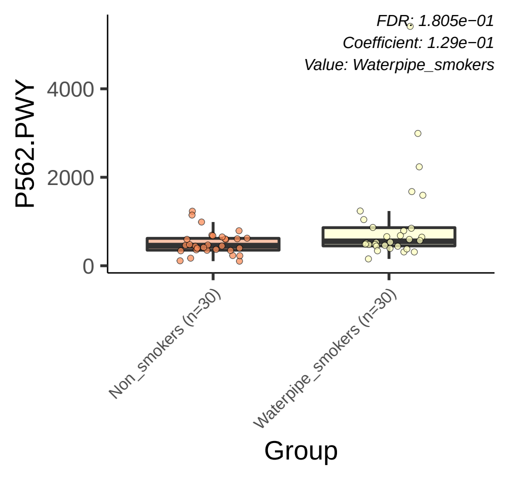
FDR: 1.805e−01
Coefficient: 1.29e−01
Value: Waterpipe_smokers
P562.PWY
4000
2000
0
Non_smokers (n=30)
Waterpipe_smokers (n=30)
Group

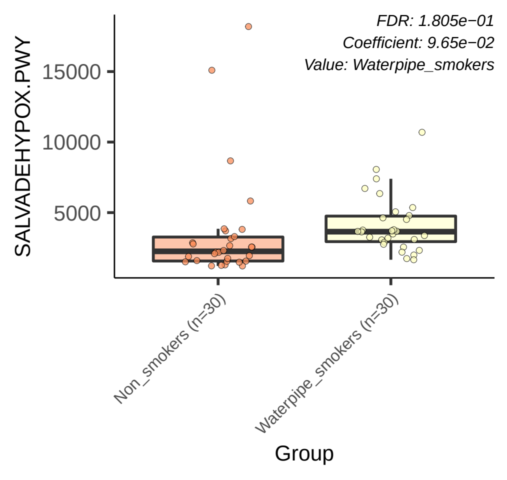

FDR: 1.805e−01
Coefficient: 9.65e−02
Value: Waterpipe_smokers
SALVADEHYPOX.PWY
15000
10000
5000
Non_smokers (n=30)
Waterpipe_smokers (n=30)
Group

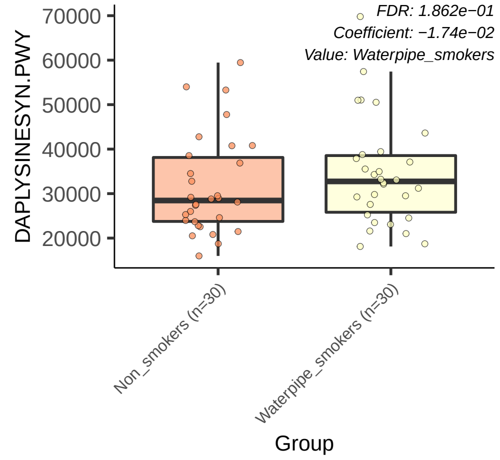

FDR: 1.862e−01
Coefficient: −1.74e−02
Value: Waterpipe_smokers
DAPLYSINESYN.PWY
70000
60000
50000
40000
30000
20000
Non_smokers (n=30)
Waterpipe_smokers (n=30)
Group

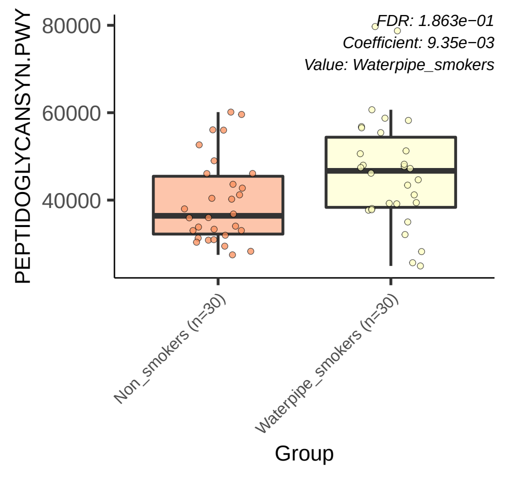
FDR: 1.863e−01
Coefficient: 9.35e−03
Value: Waterpipe_smokers
PEPTIDOGLYCANSYN.PWY
80000
60000
40000
Non_smokers (n=30)
Waterpipe_smokers (n=30)
Group

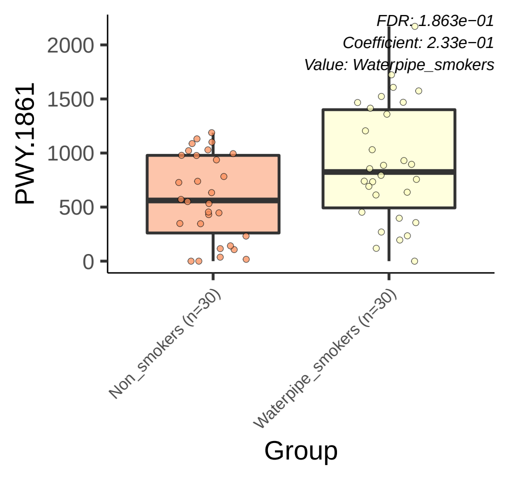
FDR: 1.863e−01
Coefficient: 2.33e−01
Value: Waterpipe_smokers
PWY.1861
2000
1500
1000
500
0
Non_smokers (n=30)
Waterpipe_smokers (n=30)
Group

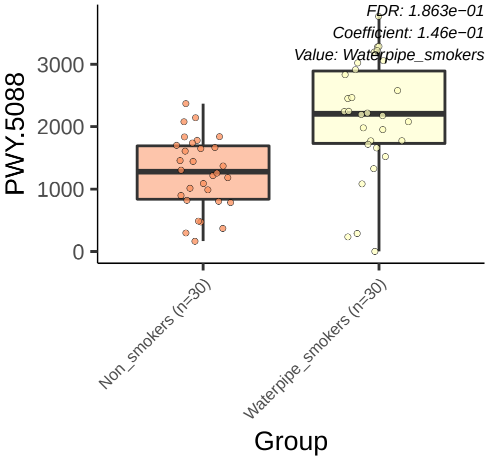
FDR: 1.863e−01
Coefficient: 1.46e−01
Value: Waterpipe_smokers
PWY.5088
3000
2000
1000
0
Non_smokers (n=30)
Waterpipe_smokers (n=30)
Group

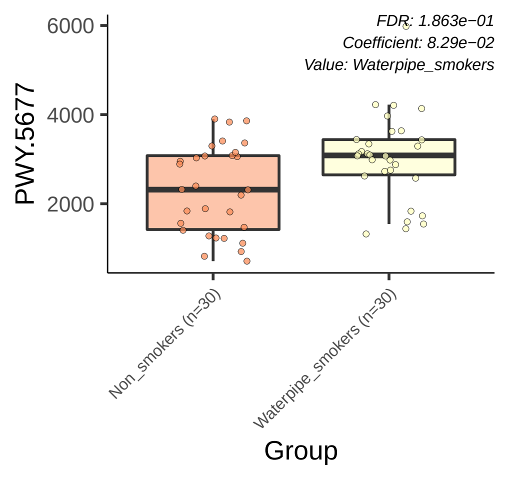
FDR: 1.863e−01
Coefficient: 8.29e−02
Value: Waterpipe_smokers
PWY.5677
6000
4000
2000
Non_smokers (n=30)
Waterpipe_smokers (n=30)
Group

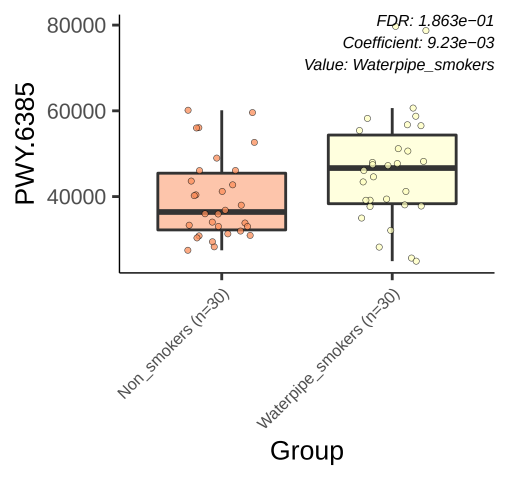

FDR: 1.863e−01
Coefficient: 9.23e−03
Value: Waterpipe_smokers
PWY.6385
80000
60000
40000
Non_smokers (n=30)
Waterpipe_smokers (n=30)
Group

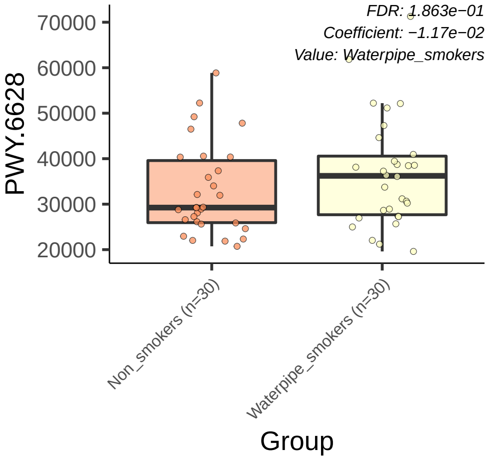

FDR: 1.863e−01
Coefficient: −1.17e−02
Value: Waterpipe_smokers
PWY.6628
70000
60000
50000
40000
30000
20000
Non_smokers (n=30)
Waterpipe_smokers (n=30)
Group

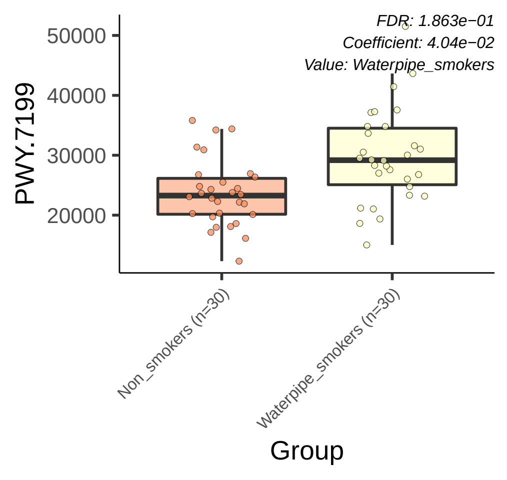
FDR: 1.863e−01
Coefficient: 4.04e−02
Value: Waterpipe_smokers
PWY.7199
50000
40000
30000
20000
Non_smokers (n=30)
Waterpipe_smokers (n=30)
Group

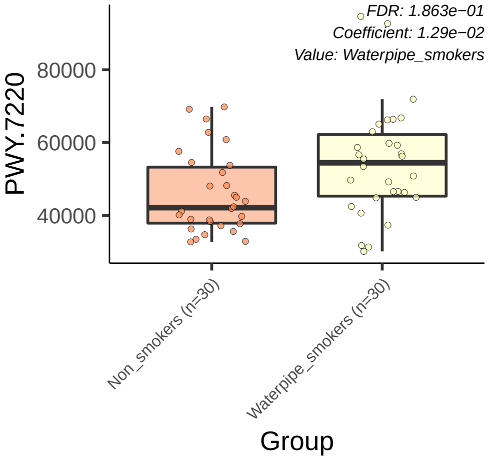

FDR: 1.863e−01
Coefficient: 1.29e−02
Value: Waterpipe_smokers
PWY.7220
80000
60000
40000
Non_smokers (n=30)
Waterpipe_smokers (n=30)
Group

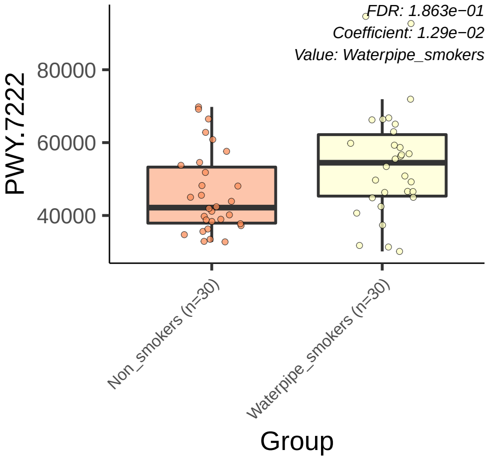

FDR: 1.863e−01
Coefficient: 1.29e−02
Value: Waterpipe_smokers
PWY.7222
80000
60000
40000
Non_smokers (n=30)
Waterpipe_smokers (n=30)
Group

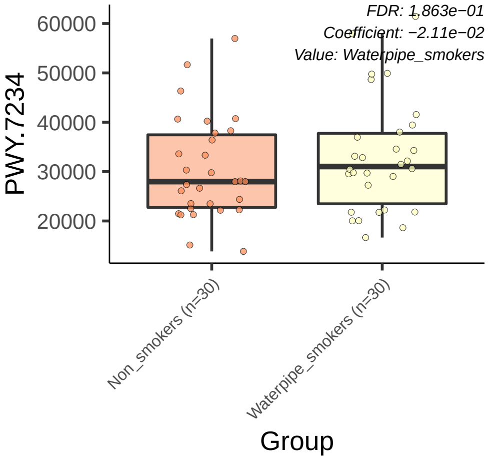
FDR: 1.863e−01
Coefficient: −2.11e−02
Value: Waterpipe_smokers
PWY.7234
60000
50000
40000
30000
20000
Non_smokers (n=30)
Waterpipe_smokers (n=30)
Group

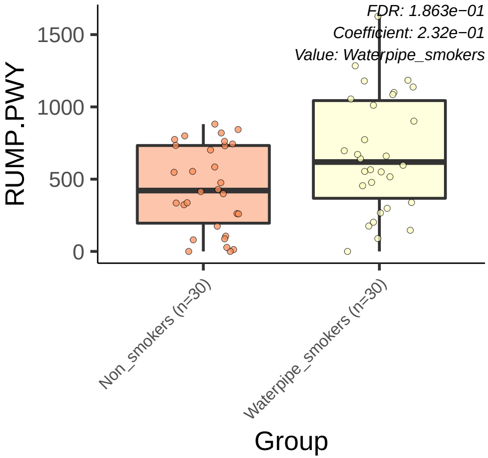

FDR: 1.863e−01
Coefficient: 2.32e−01
Value: Waterpipe_smokers
RUMP.PWY
1500
1000
500
0
Non_smokers (n=30)
Waterpipe_smokers (n=30)
Group

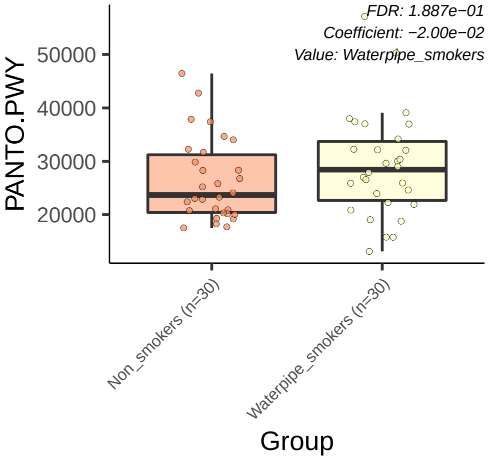
FDR: 1.887e−01
Coefficient: −2.00e−02
Value: Waterpipe_smokers
PANTO.PWY
50000
40000
30000
20000
Non_smokers (n=30)
Waterpipe_smokers (n=30)
Group

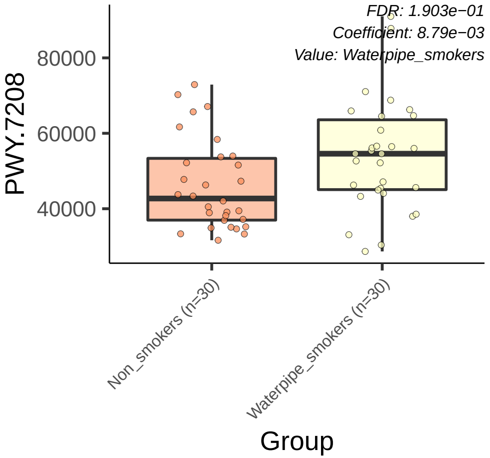
FDR: 1.903e−01
Coefficient: 8.79e−03
Value: Waterpipe_smokers
PWY.7208
80000
60000
40000
Non_smokers (n=30)
Waterpipe_smokers (n=30)
Group

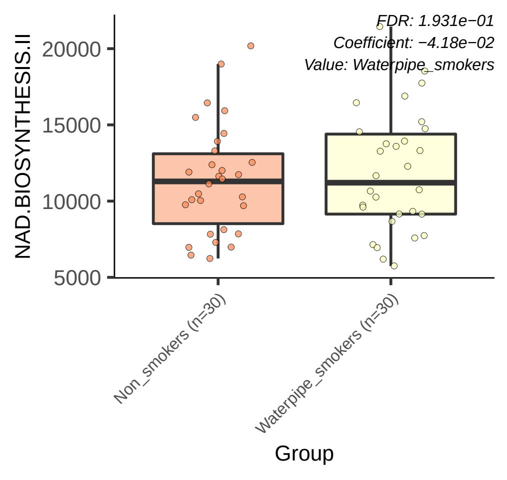
FDR: 1.931e−01
Coefficient: −4.18e−02
Value: Waterpipe_smokers
NAD.BIOSYNTHESIS.II
20000
15000
10000
5000
Non_smokers (n=30)
Waterpipe_smokers (n=30)
Group

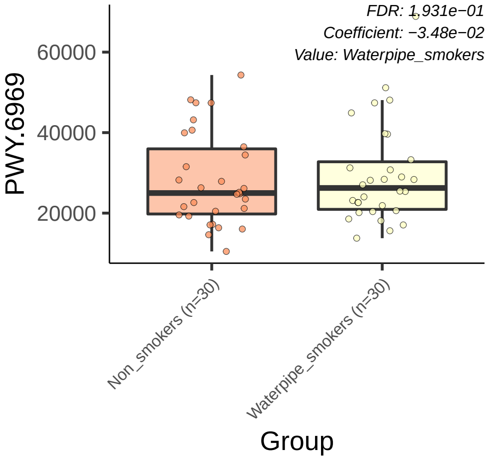

FDR: 1.931e−01
Coefficient: −3.48e−02
Value: Waterpipe_smokers
PWY.6969
60000
40000
20000
Non_smokers (n=30)
Waterpipe_smokers (n=30)
Group

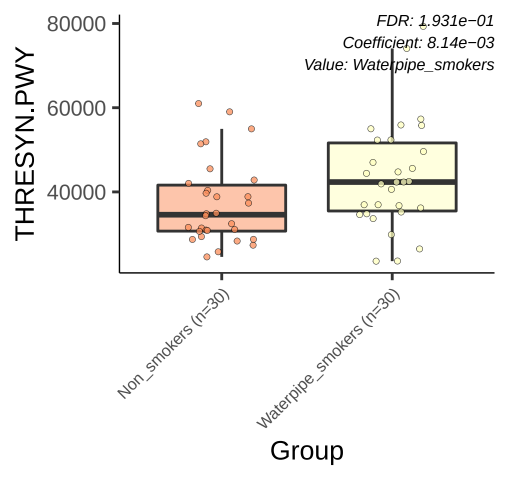

FDR: 1.931e−01
Coefficient: 8.14e−03
Value: Waterpipe_smokers
THRESYN.PWY
80000
60000
40000
Non_smokers (n=30)
Waterpipe_smokers (n=30)
Group

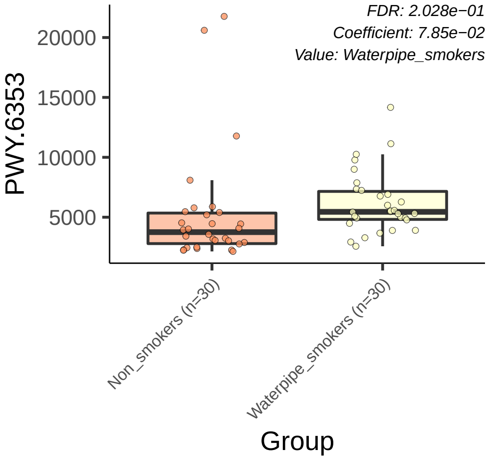

FDR: 2.028e−01
Coefficient: 7.85e−02
Value: Waterpipe_smokers
PWY.6353
20000
15000
10000
5000
Non_smokers (n=30)
Waterpipe_smokers (n=30)
Group

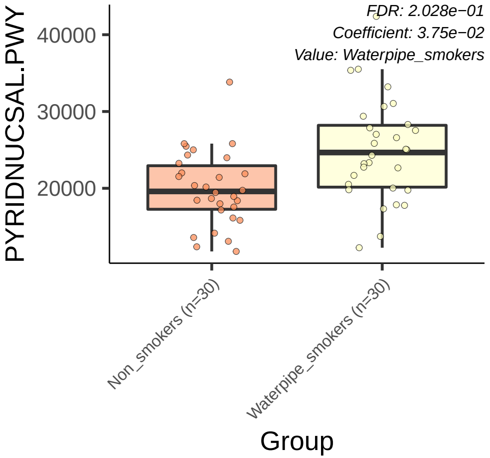
FDR: 2.028e−01
Coefficient: 3.75e−02
Value: Waterpipe_smokers
PYRIDNUCSAL.PWY
40000
30000
20000
Non_smokers (n=30)
Waterpipe_smokers (n=30)
Group

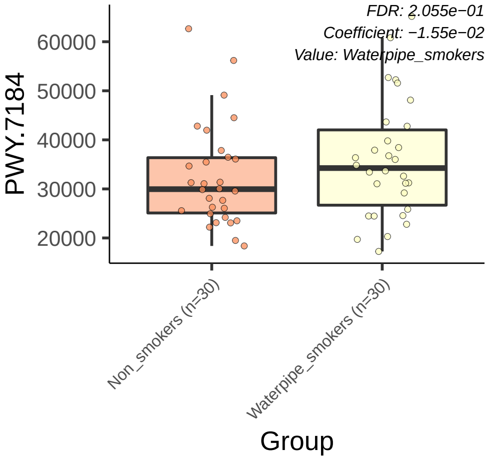
FDR: 2.055e−01
Coefficient: −1.55e−02
Value: Waterpipe_smokers
PWY.7184
60000
50000
40000
30000
20000
Non_smokers (n=30)
Waterpipe_smokers (n=30)
Group

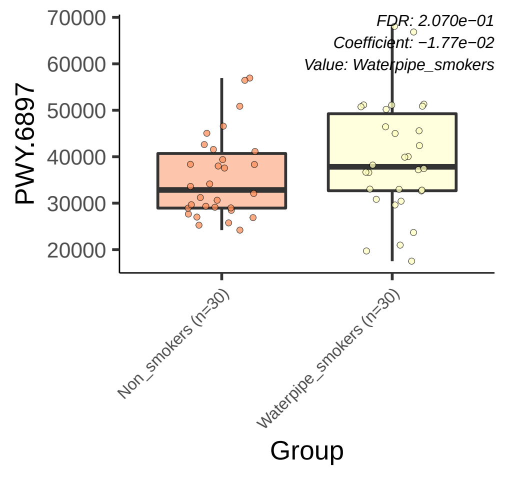

FDR: 2.070e−01
Coefficient: −1.77e−02
Value: Waterpipe_smokers
PWY.6897
70000
60000
50000
40000
30000
20000
Non_smokers (n=30)
Waterpipe_smokers (n=30)
Group

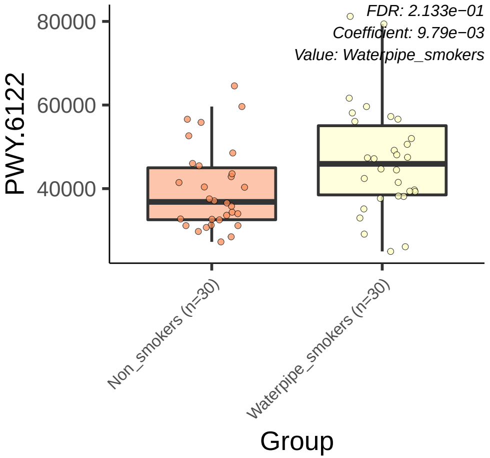
FDR: 2.133e−01
Coefficient: 9.79e−03
Value: Waterpipe_smokers
PWY.6122
80000
60000
40000
Non_smokers (n=30)
Waterpipe_smokers (n=30)
Group

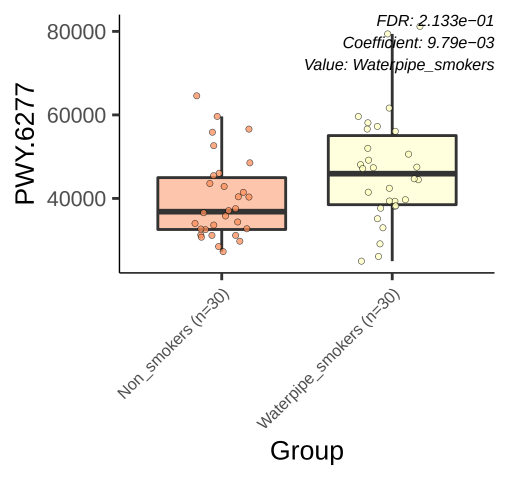

FDR: 2.133e−01
Coefficient: 9.79e−03
Value: Waterpipe_smokers
PWY.6277
80000
60000
40000
Non_smokers (n=30)
Waterpipe_smokers (n=30)
Group

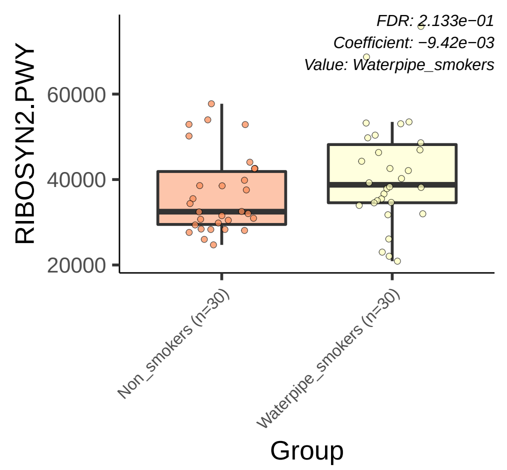
FDR: 2.133e−01
Coefficient: −9.42e−03
Value: Waterpipe_smokers
RIBOSYN2.PWY
60000
40000
20000
Non_smokers (n=30)
Waterpipe_smokers (n=30)
Group

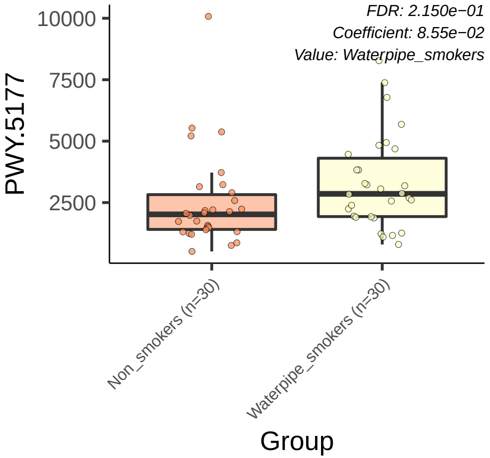
FDR: 2.150e−01
Coefficient: 8.55e−02
Value: Waterpipe_smokers
PWY.5177
10000
7500
5000
2500
Non_smokers (n=30)
Waterpipe_smokers (n=30)
Group

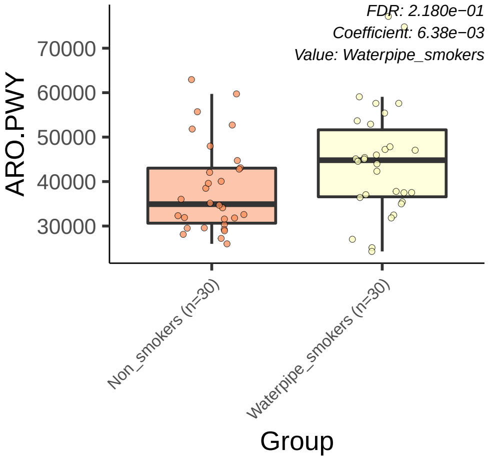
FDR: 2.180e−01
Coefficient: 6.38e−03
Value: Waterpipe_smokers
ARO.PWY
70000
60000
50000
40000
30000
Non_smokers (n=30)
Waterpipe_smokers (n=30)
Group

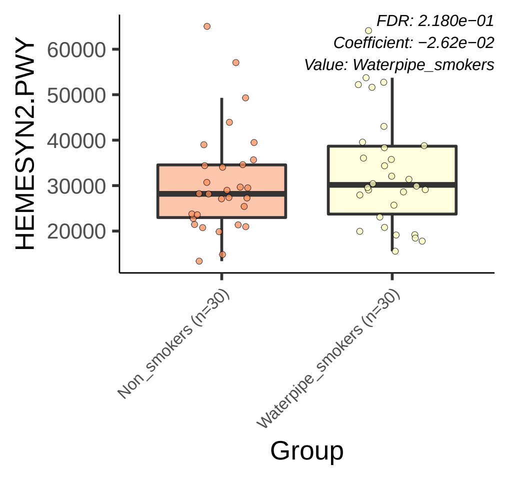

FDR: 2.180e−01
Coefficient: −2.62e−02
Value: Waterpipe_smokers
HEMESYN2.PWY
60000
50000
40000
30000
20000
Non_smokers (n=30)
Waterpipe_smokers (n=30)
Group

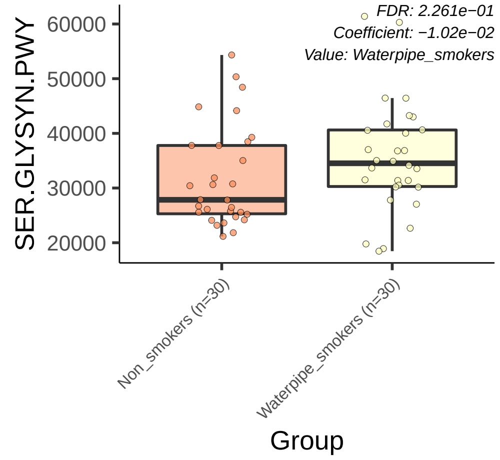
FDR: 2.261e−01
Coefficient: −1.02e−02
Value: Waterpipe_smokers
SER.GLYSYN.PWY
60000
50000
40000
30000
20000
Non_smokers (n=30)
Waterpipe_smokers (n=30)
Group

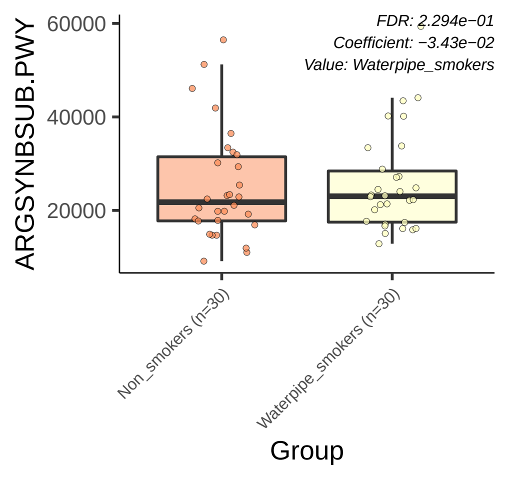

FDR: 2.294e−01
Coefficient: −3.43e−02
Value: Waterpipe_smokers
ARGSYNBSUB.PWY
60000
40000
20000
Non_smokers (n=30)
Waterpipe_smokers (n=30)
Group

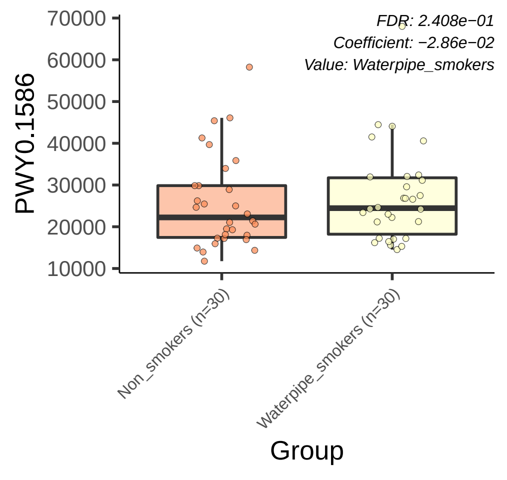
FDR: 2.408e−01
Coefficient: −2.86e−02
Value: Waterpipe_smokers
PWY0.1586
70000
60000
50000
40000
30000
20000
10000
Non_smokers (n=30)
Waterpipe_smokers (n=30)
Group

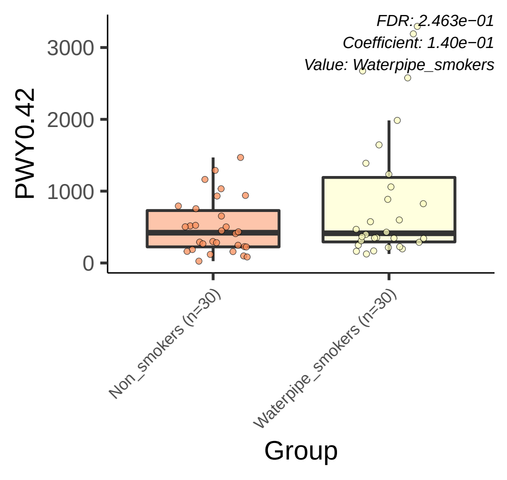
FDR: 2.463e−01
Coefficient: 1.40e−01
Value: Waterpipe_smokers
PWY0.42
3000
2000
1000
0
Non_smokers (n=30)
Waterpipe_smokers (n=30)
Group
